# The E_2_ state of FeMoco: Hydride Formation versus Fe Reduction and a Mechanism for H_2_ Evolution

**DOI:** 10.1002/chem.202102730

**Published:** 2021-10-15

**Authors:** Albert Th. Thorhallsson, Ragnar Bjornsson

**Affiliations:** ^1^ Science Institute University of Iceland Dunhagi 3 107 Reykjavik Iceland; ^2^ Department of Inorganic Spectroscopy Max-Planck-Institut für Chemische Energiekonversion Stiftstrasse 34–36 45470 Mülheim an der Ruhr Germany

**Keywords:** cofactors, density functional calculations, hydrides, nitrogenases, quantum chemistry

## Abstract

The iron‐molybdenum cofactor (FeMoco) is responsible for dinitrogen reduction in Mo nitrogenase. Unlike the resting state, E_0_, reduced states of FeMoco are much less well characterized. The E_2_ state has been proposed to contain a hydride but direct spectroscopic evidence is still lacking. The E_2_ state can, however, relax back the E_0_ state via a H_2_ side‐reaction, implying a hydride intermediate prior to H_2_ formation. This E_2_→E_0_ pathway is one of the primary mechanisms for H_2_ formation under low‐electron flux conditions. In this study we present an exploration of the energy surface of the E_2_ state. Utilizing both cluster‐continuum and QM/MM calculations, we explore various classes of E_2_ models: including terminal hydrides, bridging hydrides with a closed or open sulfide‐bridge, as well as models without. Importantly, we find the hemilability of a protonated belt‐sulfide to strongly influence the stability of hydrides. Surprisingly, non‐hydride models are found to be almost equally favorable as hydride models. While the cluster‐continuum calculations suggest multiple possibilities, QM/MM suggests only two models as contenders for the E_2_ state. These models feature either i) a bridging hydride between Fe_2_ and Fe_6_ and an open sulfide‐bridge with terminal SH on Fe_6_ (**E_2_‐hyd**) or ii) a double belt‐sulfide protonated, reduced cofactor without a hydride (**E_2_‐nonhyd**). We suggest both models as contenders for the E_2_ redox state and further calculate a mechanism for H_2_ evolution. The changes in electronic structure of FeMoco during the proposed redox‐state cycle, E_0_→E_1_→E_2_→E_0_, are discussed.

## Graphical abstract

**Hydride formation versus Fe reduction**: Hybrid quantum mechanics / molecular mechanics calculations were used to explore the the E_2_ redox state of the iron‐molybdenum cofactor of nitrogenase. Two stable isomers were found: a bridging hydride model (with an open belt sulfide‐bridge) and a non‐hydride model (with two belt sulfides protonated and reduced Fe). A path back to the resting state, by H_2_ evolution via a hydride‐proton reaction was found.

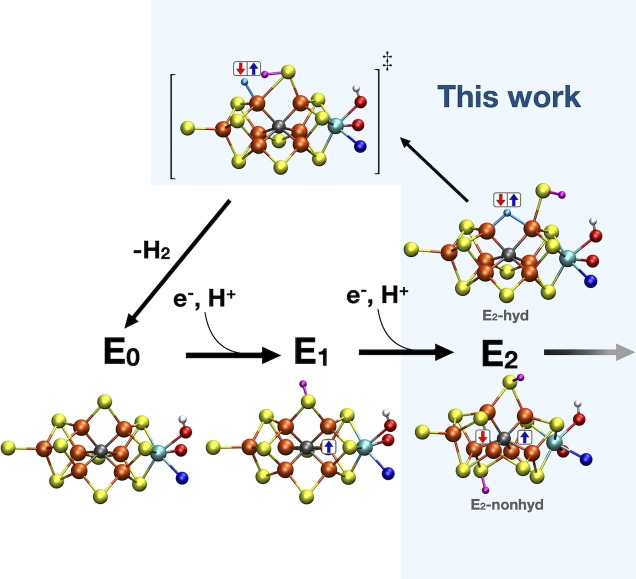

## Introduction

**Figure 1:**
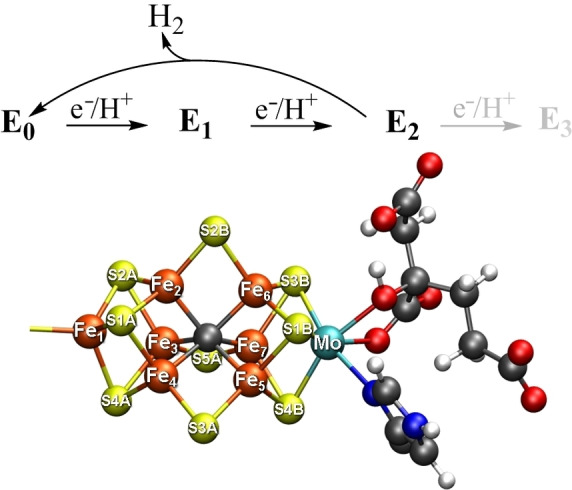
Top: A truncated Lowe–Thorneley diagram of the first reduced FeMoco states: from the resting state E_0_ (S=3/2), via the singly reduced E_1_ state (S=?), the doubly‐reduced E_2_ state (S=3/2) to the triply reduced E_3_ state (S=?). Bottom: The structure of FeMoco in the E_0_ state with atom labeling according to the X‐ray crystal structure.^[8]^

Nitrogenases are the only enzymes capable of reducing dinitrogen to ammonia. The most active Mo‐dependent variant accomplishes this via the use of a two‐protein system: Fe protein and the MoFe protein. The MoFe protein contains in its active site a complicated [MoFe_7_S_9_C] cofactor, FeMoco, responsible for the binding and reduction of dinitrogen to ammonia in an 8‐electron ATP‐dependent process: N_2_+8e^−^+8H^+^+16ATP→2NH_3_+H_2_+16ADP+16P_i_.[^1^, ^2^, ^3^, ^4^, ^5^] Unfortunately, a detailed mechanism for how the enzyme accomplishes this challenging reaction is still lacking. The Lowe‐Thorneley kinetic scheme^[6]^ connects the resting state E_0_ of the MoFe protein (and cofactor FeMoco) with reduced states E_1_‐E_7_, where N_2_ is assumed to not bind to the cofactor until after 3–4 reduction events (E_3_ or E_4_ states). Figure 1 shows a truncated diagram with a focus on the first reduced states, E_0_‐E_3_.

In addition to nitrogen reduction, nitrogenase also possesses hydrogenase activity, reducing protons and electrons to H_2_. This H_2_ evolution can either be a side‐reaction (relaxation from reduced E_n_ states, for example E_2_→E_0_ and E_4_→E_2_ ) when no substrate is present or part of the obligatory H_2_ formation during reductive elimination as N_2_ binds (E_4_+N_2_→E_4_‐N_2_+H_2_).^[7]^

An understanding of the molecular and electronic structure of each redox state of FeMoco is critical to any informed discussion of the mechanism. The E_0_ state is unfortunately the only state that is reasonably well understood and the only state that has been extensively characterized by both high‐resolution crystallography[^3^, ^8^] and multiple spectroscopic techniques.^[9]^ The redox state has been identified as [MoFe_7_S_9_C]^1−^ implying a formal oxidation state of Mo(III)3Fe(II)4Fe(III)[^10^, ^11^, ^12^, ^13^, ^14^] although mixed‐valence delocalization makes a clear physical oxidation state assignment difficult.

Due to difficulties in preparing samples containing pure redox states (this arises due to reduction being only controllable by Fe protein reductant concentration and the tendency of reduced samples to relax back to E_0_) there is only limited spectroscopic knowledge of the other states. Most of our knowledge of reduced FeMoco states has come from cryoannealing combined with spin‐selective spectroscopies such as EPR and ENDOR spectroscopy of even‐numbered redox states. Recent Mössbauer, EXAFS and QM/MM studies[^15^, ^16^] have established a likely model of the EPR‐silent E_1_ state as an Fe‐reduced, belt‐sulfide protonated FeMoco, in agreement with previous computational proposals.[^17^, ^18^, ^19^, ^20^] The most extensive spectroscopic studies have been performed on the S=1/2 E_4_ state with EPR and ENDOR that has established the state as containing two bridging hydrides and two protonated sulfide‐bridges. The precise structure of E_4_, however, is debated.[^21^, ^22^, ^23^, ^24^, ^25^]

Less is known about the E_2_ state which is the subject of this study. It is known that by annealing a trapped S=1/2 E_4_ state leads to the observation of two S=3/2 EPR signals labelled 1b (g=[4.21,3.76,?]) and 1c (g=[4.69,∼3.20,?]), the same signals that can be found in trace amounts under turnover conditions. This suggests the relaxation process:

E_4_ (S=1/2)→E_2_ (S=3/2)→E_0_ (S=3/2) (with a molecule of H_2_ evolved in each step) and kinetic isotope effects have been observed for both steps. Unfortunately, low populations of these E_2_ signals has prevented ENDOR spectroscopy from establishing whether a hydride is present in these states.^[26]^ Combined photolysis and annealing studies^[26]^ has, however, revealed 1b and 1c* (photolytically generated 1c) to be in the same redox state and two signals can be interconverted via photolysis (1b→1c*) or relaxation (1c*→1b) and a kinetic isotope effect of ∼3 suggests the involvement of a hydride in the conversion. Overall, these studies suggest that the E_2_ redox state of FeMoco possesses 2 low‐energy structural isomers (within ∼1–2 kcal/mol of each other), at least one of which contains a hydride. In 2017, Khadka et al.^[27]^ were able to study the H_2_ evolution reaction via mediated bioelectrocatalysis. They established that the rate‐limiting step for H_2_ formation under those conditions (when decoupled from the more complex Fe protein electron delivery process that is otherwise rate‐limiting) is neither electron nor proton delivery, but H−H bond formation. Furthermore, analysis of the catalytic currents with varying H_2_O/D_2_O ratios indicates that a single H/D is involved, which with the help of calculations was interpreted as a sulfide‐bound proton attacking an Fe hydride.

Previous computational studies have suggested various models for the E_2_ state of FeMoco. The most extensive study is the one by Ryde and co‐workers^[17]^ where several E_n_ states were studied. Models featuring bridging or terminal hydrides with a protonated belt sulfide‐bridge were found to be the most likely models based on relative energies when using the non‐hybrid TPSS level of theory while when the hybrid B3LYP level of theory was employed, hydride models were disfavored and carbide‐protonated models or non‐belt sulfide protonated models were more stable.

The present study proposes new structural models for the E_2_ state of FeMoco based on a QM/MM modelling approach that has previously been used in our group for modelling of E_0_,^[14]^ E_1_ ^[15]^ and E_4_ ^[21]^ redox states. Our protocol combines QM/MM with a broken‐symmetry DFT approach (using the TPSSh functional, large relativistically recontracted triple‐zeta basis sets, scalar relativistic (ZORA) and dispersion corrections) that has been validated on the well‐characterized E_0_ state, by comparison of calculated FeMoco geometries to the high‐resolution crystal structure. Our models are compared directly to previously proposed models for the E_2_ redox state. The electronic structures of the lowest energy models are discussed and we suggest a possible connection to the two distinct EPR signals attributed to the E_2_ redox state. Furthermore, we discuss a mechanism for H_2_ evolution from the most stable hydride‐based model.

## Computational details

The QM/MM modelling approach for E_2_ is based on our model for the E_0_ resting state model that has been previously described and used in other studies.[^14^, ^16^, ^21^] It is a spherical QM/MM model (42 Å radius) centered on the carbide of FeMoco that includes roughly half of the dimeric MoFe protein. In the QM/MM geometry optimizations the active region consists of 1001 atoms (centered around FeMoco) and a QM region of 134 atoms. All QM/MM calculations were performed in Chemshell[^28^, ^29^] using the built‐in MM code DL_POLY^[30]^ with the CHARMM36 forcefield^[31]^ and ORCA version 4.0^[32]^ as QM code. The QM region contains the FeMoco cofactor, singly protonated R‐homocitrate and the sidechains of residues α‐191^Gln^, α‐195^His^, α‐442^His^, α‐275^Cys^, α‐96^Arg^, α‐359^Arg^, α‐381^Phe^, α‐70^Val^, α‐380^Glu^, α‐192^Ser^, as well. This large QM‐region was used in all QM/MM calculations described in the manuscript and includes all protein residues bonded to the cofactor (α‐442^His^, α‐275^Cys^), all near‐by charged residues (α‐96^Arg^, α‐359^Arg^, α‐380^Glu^), residues with sidechains engaging in hydrogen‐bonds to the cofactor (α‐191^Gln^, α‐195^His^, α‐192^Ser^) and residues spatially close to the Fe2,3,6,7 face (α‐381^Phe^, α‐70^Val^). A larger QM‐region was briefly explored as discussed in the Supporting Information (Figure S9) where the QM‐region was expanded to also include the peptide backbone of residues surrounding S3A; the energies of hydride isomers changed by 0.1–0.7 kcal/mol. All QM/MM calculations used electrostatic embedding, and link atoms were used to terminate the QM−MM border together with the charge‐shift procedure as implemented in Chemshell. The QM calculations used the TPSSh hybrid density functional,[^33^, ^34^] ZORA scalar relativistic Hamiltonian[^35^, ^36^] the relativistically recontracted def2‐TZVP basis set,[^37^, ^38^] on all metal, sulfide, carbide and hydride/SH atoms (ZORA‐recontracted def2‐SVP on other atoms) and the D3 dispersion correction.[^39^, ^40^] The RIJCOSX approximation[^41^, ^42^] with a Coulomb auxiliary basis set by Weigend was used.^[43]^ Broken‐symmetry solutions of the E_2_ models were found by flipping spins on Fe atoms, starting from the ferromagnetic M_S_=35/2 solution and converging to a M_S_=3/2 solution for each spin‐flip. We have previously emphasized the importance of accounting for multiple broken‐symmetry solutions in studies of FeMoco. The three lowest energy BS solutions for E_0_ are of the BS7 class: BS7‐235, BS7‐346 and BS7‐247 that change the location of mixed‐valence pairs and localized ferrous/ferric sites.^[14]^ In a previous study of E_4_ we also found that the BS10‐147 solution could for some structures become the most stable. Hence these 4 BS solutions were calculated for different structural models as discussed in the Results and Discussion. This assumption was also challenged later in the study by a systematic comparison of nearly all BS solutions (representatives of all 10 BS classes) for the lowest energy models for each class (terminal, bridging‐hydride with closed‐bridge, bridging hydride with open‐bridge and non‐hydride) as shown in Tables S16‐S19 in the Supporting Information. Cluster‐continuum calculations were carried out in the same way but utilizing a CPCM continuum model^[44]^ instead of a QM/MM environment. The cluster‐continuum calculations utilized a minimal model for FeMoco consisting of the cluster, a thiomethyl group on Fe, a methylimidazole group on Mo and a triply protonated homocitrate (on alcohol group and the two distal carboxylate groups) in order to reduce the high negative charge. A dielectric constant of ϵ=4 was used, and the QM‐continuum interaction was described by a Gaussian‐pointcharge scheme^[45]^ and a scaled vdW cavity surface.^[46]^ Nudged elastic band[^47^, ^48^, ^49^] calculations were performed using the Chemshell implementation^[50]^ in order to locate approximate saddlepoints that were subsequently refined using the dimer method.[^51^, ^52^] QM/MM partial Hessian vibrational frequency calculations were carried out for selected models (see section C and E) using Chemshell utilizing the same QM and active region but with only a subset of atoms included in the numerical Hessian calculation. The Hessian “region” includes cofactor atoms (MoFe_7_S_9_CH_2_), the S‐atom of α‐275^Cys^, the N‐atom of α‐442^His^ and the Mo‐bound O‐atoms of homocitrate (as well as the alcohol proton). Localized‐orbital analysis of broken‐symmetry solutions was performed using the Pipek‐Mezey approach.^[53]^

## Results and Discussion

The article is organized as follows. Section A introduces the structural models that we explore in our study and the model labelling that will be used throughout. In Section B we discuss the results of a DFT minimal cluster modelling approach while Section C shows the results of QM/MM calculations and the influence of the protein environment on the stability of models. Section D features analysis of the electronic structure of the most favorable E_2_ models and finally in section E we discuss a possible mechanism of H_2_ evolution based on our models.

### A Classification of E_2_ models

Previous work from our group established a likely structural model for the E_1_ redox state,^[16]^ on the basis of QM/MM calculations constrained by Fe,Mo EXAFS. The QM/MM‐optimized structures were compared to the EXAFS structural data and calculated relative energies were used to further distinguish between structural models consistent with the EXAFS data. The study could discount hydride‐based models for E_1_ and suggested instead the E_1_ state to feature Fe‐based reduction (specifically in the MoFe_3_S_3_C sub‐cubane, according to the calculations) and a protonated bridging sulfide at either S2B (bridging Fe_2_ and Fe_6_) or S5A (bridging Fe_3_ and Fe_7_). The S2B‐protonated model might be considered a more likely model for E_1_ in view of crystal structures revealing that this particular sulfide can be replaced by alternative ligands such as: Se^2−^,^[54]^ CO,[^55^, ^56^] OH[^57^, ^58^] (FeVco). However, both S2B and S5A protonation sites have almost equal basicity^[16]^ (while S3A is in a sterically hindered environment that does not favor protonation, see Figure S13) and should thus be given equal consideration. Another QM/MM study found a preference for S2B.^[17]^ We also note that Se incorporation via selenocyanate under turnover has even revealed that all 3 belt sulfides can be exchanged.^[55]^

**Figure 2:**
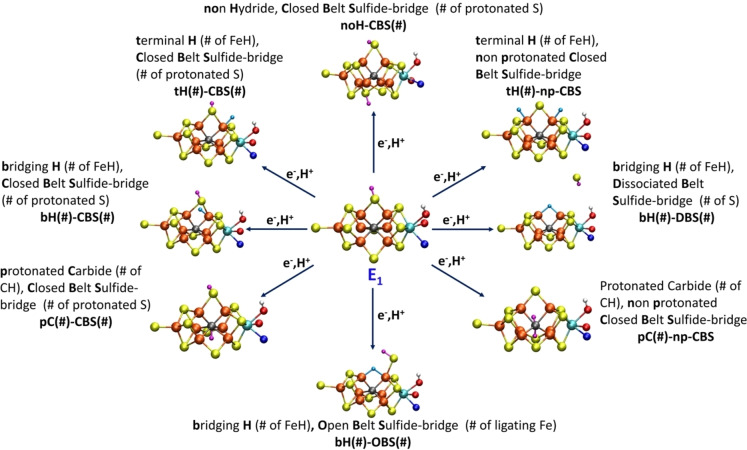
Different model classes for the E_2_ redox state investigated in this work. Structures shown are representative of the multiple structures of the same class. Hydrides (H‐atoms bound to Fe) are shown in aqua color, while S‐bound protons are shown as magenta and the proton on Mo‐bound alcohol group is shown as white. Fe atoms are shown as orange, S atoms as yellow, Mo as teal, O as red and N as blue.

The model for E_1_ offers a convenient starting point for a discussion of structural models for the E_2_ redox state as shown in Figure 2. From an E_1_ model featuring a reduced Fe‐part (in the MoFe_3_S_3_C sub‐cubane^[16]^) and a protonated bridging sulfide (either at S2B or S5A) we can imagine many different E_2_ models that can be grouped into several classes. Our classification scheme consists of: non‐hydride models with 2 protonated closed belt sulfide‐bridges (**noH‐CBS**) and two metal‐based reductions, terminal hydride models with 1 protonated closed belt sulfide‐bridge (**tH‐CBS**), bridging hydride models with 1 protonated closed belt sulfide‐bridge (**bH‐CBS**), models with 2 terminal hydrides without a protonated belt sulfide‐bridge (**tH‐npCBS**), models with 1 bridging hydride and a protonated open belt sulfide‐bridge (**bH‐OBS**), models with a bridging hydride and a dissociated belt‐sulfide (**bH‐DBS**) (belt sulfide dissociated in the form of either SH^−^ or H_2_S) and finally models featuring a protonated carbide (**pC‐CBS**). We assume an equal number of protons and electrons in all E_2_ models in this study. Furthermore, all terminal‐hydride models were limited to hydrides bound to belt Fe ions while **bH‐CBS** and **bH‐OBS** models were limited to structures where the hydride bridges the two sub‐cubanes of FeMoco. The orientations of the added protons when bound to the belt sulfides (in either CBS or OBS form) as well as the orientations of the hydrides, is also an important aspect to consider in these models; this is discussed separately in the Supporting Information.

Many of these classes of models have featured in previous computational work but there have been few systematic studies comparing multiple model classes. An exception to this is the work of Ryde and co‐workers^[17]^ which features a systematic study of multiple models for the E_0‐4_ redox states. The E_2_ models considered in that work included **noH‐CBS**, **tH‐CBS**, **pC‐CBS and bH‐CBS** classes and the authors suggested models of the **bH‐CBS**, **tH‐CBS** models as likely. Many of the models in the work by Ryde and coworkers are included here but models of the **bH‐OBS** type were not included in their work. Additionally, Dance has found **tH‐CBS**, **bH‐CBS**, **tH‐np‐CBS** and η^2^‐H_2_‐bound models to be favorable.^[19]^ Raugei, Hoffman, Seefeldt and coworkers have suggested a model of the **bH‐CBS** class as a likely model for E_2_.^[27]^ These model classes should be representative of most of the possible E_2_ models that can be imagined. We have only briefly explored protonated carbide models (two in this work), suggested by Adamo and coworkers^[59]^ for E_1_ and E_2_ redox states, with carbide‐protonated models also featuring prominently in the work of Siegbahn.^[60]^ Such protonated carbide models were explored by Ryde and coworkers in their work for E_1_ and E_2_ redox states and found to be only feasible when employing hybrid functionals with a medium‐to‐large amount of HF exchange like B3LYP. As discussed recently by Ryde^[61]^ and us,^[21]^ protonated carbide models for reduced FeMoco states appear to be only favored with computational protocols with HF exchange >20 %. In our recent study, we systematically studied the functional dependence on the structure of FeMoco in the E_0_ state with many common functionals utilizing our QM/MM model with large triple‐zeta basis set and the ZORA scalar relativistic Hamiltonian. Functionals with HF exchange >20 % systematically overestimate Fe−Fe, Mo−Fe, Fe−S, Fe−C and Mo−S distances, suggesting these functionals to be unsuitable to describe the complex electronic structure of FeMoco. Non‐hybrid functionals on the other hand appear to underestimate the same distances while the hybrid TPSSh functional (with 10 % HF exchange) appears to give a balanced description of the electronic structure as seen in the lower errors for these distances and a lack of systematic overestimation or underestimation. As shown in Figure S10 in the Supporting Information, our TPSSh‐QM/MM protocol finds protonated carbide models to be unstable by 19–31 kcal/mol compared to the most stable hydride model. Protonated carbide models will hence not be further discussed.

New in this work is the inclusion of E_2_ models of the **bH‐OBS** class where the belt sulfide‐bridge is open (but not dissociated). Here the protonated belt sulfide has reorganized into a terminal sulfhydryl group but these types of models have surprisingly never been previously discussed for the E_2_ redox state (including the extensive study by Ryde and coworkers). These **bH‐OBS** models imply a hemilability[^62^, ^63^] of the belt sulfides of FeMoco under turnover conditions while a **bH‐DBS** model would imply even greater lability. We note that X‐ray crystallography of MoFe protein have revealed CO‐inhibited FeMoco structures with a dissociated S2B belt sulfide[^55^, ^56^] while a recent VFe protein crystal structure^[57]^ exhibits a bridging OH ligand,^[58]^ the OH ligand having replaced the S2B belt sulfide in the Fe_2_/Fe_6_ position, with the dissociated sulfide present nearby (presumably in the form of SH^−^ or H_2_S). More recently an article describing an X‐ray structure showing the replacement of belt sulfides by N_2_ ligands was published^[64]^ though this claim is debated.[^65^, ^66^, ^67^]

Experimentally it is known that low electron‐flux conditions lead to new S=3/2 EPR signals (referred to as 1b and 1c) that have been assigned to the E_2_ state.^[9]^ Our broken‐symmetry state protocol for E_2_ will hence be limited to calculating broken‐symmetry states with M_S_=3/2. There are, however, many broken‐symmetry states to be potentially explored. As discussed originally by Noodleman^[68]^ and explored systematically in many recent computational studies[^21^, ^69^] there are 35 BS states for the E_0_ state, assuming collinear spin‐alignment of Fe local spins alone. This is a simplification as Mo is now known to be open‐shell as well,[^10^, ^11^] however, it is excluded from our spin‐flipping procedure as it is known to feature a non‐Hund electron configuration that is hard to control by the spin‐flip procedure. Calculations usually converge automatically to the non‐Hund configuration for Mo (seen by the slightly negative spin population); in our work we do not include the Mo ion in the spin‐flip procedure but rather monitor the Mo spin population and attempt spin‐flipping when needed. This study includes the generally favorable BS7‐type states (that maximize antiferromagnetic coupling for E_0_), as well as the BS10‐147 solution that has previously been found to be low in energy for hydride‐models of the S=1/2 E_4_ redox state.^[21]^ This approach is justified based on our previous work on E_0_ ^[14]^ and E_1_ ^[21]^ models (BS7‐235, BS7‐346 and BS7‐247 being the most stable BS states, within 0–2 kcal/mol of each other) and E_4_ (BS7‐235, BS7‐346, BS7‐247, and BS10‐147 most stable). Additional BS solutions were explored as discussed in section B.

### B Minimal cluster model calculations

A simple cluster‐continuum approach allows us to systematically assess the thermodynamic stability associated with the different classes of models described in section A, without effects of the protein environment. While a minimal 60‐atom cluster model consisting of only the FeMoco cluster (with α‐275^Cys^ described as a thiomethyl ligand and α‐442^His^ described as a methylimidazole ligand) cannot satisfactorily account for the electrostatic protein environment nor the conformational effects exerted on the cluster by the protein, it should nonetheless be able to reveal the most important trends in the stability of the electronic structure in terms of hydride coordination.

**Figure 3:**
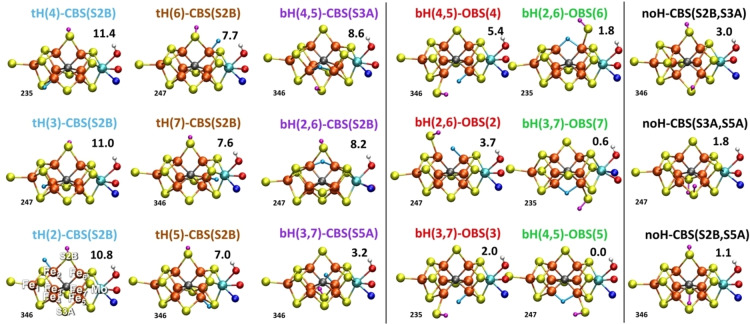
Relative energies (kcal/mol) of different E_2_ models (only most stable BS solution shown) calculated with a cluster‐continuum approach. The cluster model includes R‐homocitrate (with three added protons on the alcohol and the carboxylate groups), Mo‐ligated methylimidazole (α‐442^His^) and an Fe_1_‐ligated thiomethyl group (α‐275^Cys^). All models were calculated with 4 different broken‐symmetry solutions with the lowest‐energy one indicated to the left of each model (the 3 numbers indicating spin‐down Fe atoms). See Table S1 in the Supporting Information for information on each broken‐symmetry solution.

The results, shown in Figure 3, reveal notable trends among similar models. Terminal hydride models (**tH‐CBS**) are generally found to be unfavorable (7.0–11.4 kcal/mol, relative to the lowest energy model), and are particularly unfavorable when the hydride is present in the Fe_4_S_3_ sub‐cubane (this can be rationalized by the fact that in our E_1_ study^[16]^ the MoFe_3_S_3_C cubane was more easily reduced than the Fe_4_S_3_ sub‐cubane). Bridging hydride models with a closed belt sulfide‐bridge (**bH‐CBS**) are overall not much more favorable except for **bH(3,7)‐CBS(S5A)**, with a relative energy of 3.2 kcal/mol (compared to the lowest energy model). Bridging hydride models with an open belt sulfide (**bH‐OBS**), however, reveal a rather different trend, being overall more favorable than closed belt sulfide models. Models with a terminal sulfhydryl group in the MoFe_3_S_3_C‐subcubane are especially favorable: **bH(2,6)‐OBS(6)**, **bH(3,7)‐OBS(7)**, **bH(4,5)‐OBS(5)** have relative energies of 1.8, 0.6 and 0.0 kcal/mol. Finally, the nonhydride models **noH‐CBS(S2B,S3A)**, **noH‐CBS(S3A,S5A)**, **noH‐CBS(S2B,S5A)**, with two protonated belt sulfides instead of a hydrides, are found to be surprisingly stable as well (1.1, 1.8 and 3.0 kcal/mol).

In order to test whether our choice of exploring 4 broken‐symmetry solutions (BS7‐235, BS7‐346, BS7‐247 and BS10‐147) is sufficient to find the lowest energy electronic state for these models featuring different hydride and sulfide coordination, we separately tested more solutions for representative models of each class in Figure 3: **tH(5)‐CBS(S2B)**, **bH(2,6)‐CBS(S2B)**, **bH(4,5)‐OBS(5)** and **noH‐CBS(S2B,S5A)** by geometry optimizations for each BS state. The results, shown in Tables S16–S19 in the Supporting Information, reveal that the BS7 solutions are consistently the lowest in energy, when compared against solutions from the BS1‐10 class.

While the results reveal a strong dependence of the stability of E_2_‐hydride models on the Fe involved in hydride formation (MoFe_3_S_3_C‐subcubane vs. Fe_4_S_3_C‐subcubane), bridging vs. terminal hydride geometry and whether the protonated sulfhydryl group is open or bridging, the trends are not easily understood. Tables S4–S6 in the Supporting Information compare the Fe−H bond lengths, Mayer bond orders[^70^, ^71^, ^72^] and Hirshfeld charges^[73]^ of the hydride models in Figure 3. The distances, bond orders and atomic charges change with each E_2_‐hydride model and broken‐symmetry solution, but there is no obvious correlation present that explains the stability of the models. Most likely the stability of a given E_2_ model arises both from stability of the Fe‐hydride bond formed, its hydricity as well as the changing spin‐coupling within the cofactor.

### C QM/MM calculations of E_2_ models

The cluster‐continuum calculations implicate models featuring bridging hydrides with an open belt sulfide‐bridge (**bH‐OBS** models) as well as models without hydrides (**noH** models). However, the opening of a belt sulfide bridge as shown would be expected to be strongly dependent on the protein environment as a terminal sulfhydryl group may clash with protein residues. It thus becomes necessary to consider a more realistic model of FeMoco in its protein environment and hence we move to QM/MM calculations. Our QM/MM calculations feature enlarged QM‐regions (134 atoms) and most residues surrounding the cofactor are thus described quantum mechanically. As the α‐195^His^ residue makes a direct hydrogen‐bond to the S2B sulfide according to the crystal structure of the E_0_ state, we have paid special attention to this residue. Since this residue may be involved in protonation of the cofactor we have previously speculated[^16^, ^21^] that the protonation state of α‐195^His^ could be inversed in reduced FeMoco states; being possibly protonated in the N_δ_ position instead of the N_ϵ_ position (as in the E_0_ state) due to a Grotthuss‐type mechanism for proton‐transfer to the cofactor. Based on a simple energy comparison of E_0_, E_1_ and E_2_ models the N_ϵ_(H) protonation state is predicted to be ∼20 kcal/more stable. However, the stability of α‐195^His^ N_ϵ_ and N_δ_ proton isomers likely strongly depends on the hydrogen‐bonding network between between α‐195^His^ and α‐281^Tyr^ with water molecules that could rearrange depending on redox state; furthermore the QM/MM model is originally prepared based on the E_0_ X‐ray structure with α‐195^His^‐N_ϵ_(H), and hence has a strong bias towards that state. We have thus chosen to consider both α‐195^His^ protonation isomers in this study.

**Figure 4:**
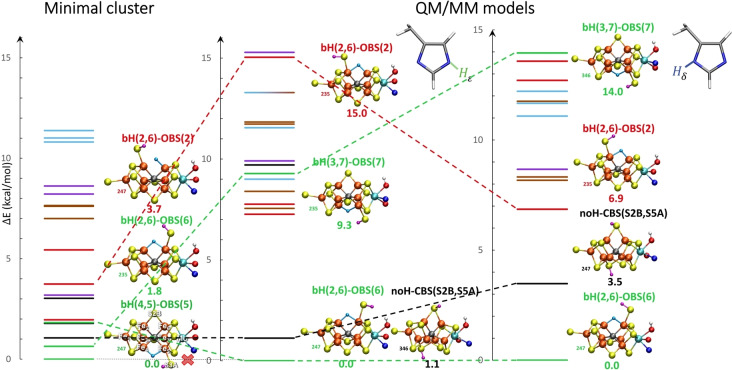
Relative energies of E_2_ models, calculated with a minimal cluster‐model approach level (left) and at the QM/MM level in either the α‐195^His^‐N_ϵ_(H) protonation state (middle) or the α‐195^His^‐N_δ_(H) protonation state (right). Only the lowest‐energy BS solution is shown (see Supporting Information for a table of all energies). The α‐195^His^‐N_ϵ_(H) state ladder is predicted to be more stable in energy than α‐195^His^‐N_δ_(H) (by ∼21 kcal/mol), though we note that the QM/MM model is originally modelled based on the E_0_ X‐ray structure with α‐195^His^‐N_ϵ_(H) and hence strongly biased towards that state.

In going from a minimal cluster to QM/MM environment we note a dramatic shift in the energy landscape as shown in Figure 4. The range of relative energies of **bH‐CBS** and **tH‐CBS** models at the QM/MM level is sparser compared to minimal cluster models and the protein environment stabilizes or destabilizes several models by several kcal/mol. The lowest energy hydride model with α‐195^His^‐N_ϵ_(H) protonation state remains an open belt sulfide model but unlike the minimal cluster approach the model with a bridging hydride between Fe_2_ and Fe_6_, **bH(2,6)‐OBS(6)**, is now favored. Curiously, the **bH(4,5)‐OBS(5)** model which is favored using the minimal cluster‐continuum approach, is not present on the QM/MM potential energy surface (turning into **tH(5)‐OBS(4)**). This effect clearly arises due to the protein backbone being close to the Fe_4_‐S3A‐Fe_5_ belt position (up to five weak NH⋯S3A hydrogen bonds as shown in Figure S13) and emphasizes the importance of accounting accurately for protein environmental effects in calculations of FeMoco. We separately explored including the peptide backbone surrounding S3A in the QM‐region (see Figure S9 in the Supporting Information) but this was found to have marginal effects on the energies while considerable increasing the computational cost. The protein environment also strongly influences the stability of **bH‐OBS** models with models being strongly preferred when the SH group is present on Fe_6_/Fe_5_/Fe_7_ atoms (i. e. in the MoFe_3_S_3_C sub‐cubane) instead of Fe_2_/Fe_3_/Fe_4_ (Fe sub‐cubane). We note especially the relative energy of the **bH(2,6)‐OBS(2)** and **bH(3,7)‐OBS(7)** models that are strongly affected by the presence and protonation state of α‐195^His^.

**bH‐DBS** models, where the sulfide has dissociated from the cofactor, which has been suggested to occur at the E_2_ level,[^3^, ^57^] were only partially investigated in this work (not included in Figures 3 and 4). A QM/MM model with a large extended QM region was used to test the feasibility of such a model where an SH^−^ ion has dissociated to occupy a similar position (near the α‐191^Gln^ residue) as the sulfide in the ligand‐bound FeVco structure.^[57]^ The results are shown in the Supporting Information but reveal that SH^−^ dissociation is strongly disfavored (by 56 kcal/mol) compared to the **bH(2,6)‐OBS(6)** model. While multiple crystal structures[^54^, ^55^, ^56^, ^57^] have revealed that the S2B belt sulfide bridging Fe ions 2 and 6, can be displaced and replaced by other ligands, it is not yet established whether sulfide dissociation takes place under physiological turnover conditions and if it does, in which redox state. Further computational studies of possible S2B sulfide dissociation would be desirable but are outside the scope of this study.

Overall, the QM/MM calculations strongly affect the energy landscape of the E_2_ hydride isomers, with even the position of a single proton at α‐195^His^ affecting the relative energies. Importantly, however, the QM/MM calculations for both α‐195^His^ protonation states are in good agreement regarding the stability of the lowest energy hydride model: **bH(2,6)‐OBS(6)**, hereafter labelled as **E_2_‐hyd**, being favored by at least ∼6 kcal/mol over all other hydride models. Interestingly, however, a model without a hydride, **E_2_‐nonhyd** (**noH‐CBS(S2B,S5A)**), is predicted to be an equally likely model, being slightly higher in energy by 1.1 kcal/mol than **E_2_‐hyd** in the regular α‐195^His^‐N_ϵ_(H) protonation state and close in energy for the α‐195^His^‐N_δ_(H) state (+3.5 kcal/mol higher than **E_2_‐hyd**). Both **E_2_‐nonhyd** and **E_2_‐hyd** models thus emerge as equally likely candidates for the E_2_ state of FeMoco while other models seem considerably less likely.

The accuracy of our results (that differ from previous studies) depend, in addition to how the protein environment is described, on the density functional approximation in use, here TPSSh as well as on the basis set used (here ZORA‐def2‐TZVP on the cofactor atoms). We have previously argued^[21]^ that the TPSSh functional describes the electronic structure of FeMoco better than other functionals on the basis of the much better agreement of calculated structural parameters of FeMoco in the E_0_ state compared to the high‐resolution X‐ray crystal structure, implying a more balanced treatment of the super‐exchange and delocalization effects present in this complex cofactor. We have tested systematically the effect of basis set (def2‐SVP vs. def2‐TZVP) and functional choice (TPSS vs. TPSSh) by comparison of energies of multiple E_2_ isomers (**bH‐CBS**, **bH‐OBS**, **bH‐CBS**, **tH‐CBS** and **noH‐CBS**) by full geometry optimizations using the cluster‐continuum model, as seen in Table S8 in the Supporting Information. The results reveal that the energetic ordering of isomers is strongly dependent on both the size of the basis set (def2‐SVP vs. def2‐TZVP) as well as whether using the non‐hybrid functional TPSS or the hybrid‐functional TPSSh. It is clear that using a large basis set is highly important for removing basis set errors in calculations of FeMoco isomers (ZORA‐def2‐TZVP on all cofactor atoms was generally used in this work) and that the functional choice has an especially large effect. This strong dependence on both basis set and functional is likely the reason why Cao and Ryde^[17]^ came to slightly different conclusions about the stability of **noH‐CBS** E_2_ isomers in their detailed QM/MM study than we have. In their work, a small def2‐SV(P) basis set was used for geometry optimizations (with subsequent def2‐TZVPD single‐point energy evaluations) using either a TPSS or B3LYP functional. Finally, **bH‐OBS** isomers were not included in their study. It is clear, however, that more work is needed to develop a better understanding of the density functional dependence on the electronic structure of FeMoco, especially reduced states, a current topic of discussion in the literature.[^21^, ^61^, ^74^] Finally, we note that zero‐point vibrational energy (ZPVE) contributions to the energy have in general not been included in this study. We tested the effects of including ZPVE via a QM/MM partial Hessian calculation of **E_2_‐nonhyd** and **E_2_‐hyd** models that resulted in a relatively small ΔZPVE contribution of 0.61 kcal/mol (further stabilizing **E_2_‐hyd**).

### D Electronic structure of E_2_‐hyd versus E_2_‐nonhyd

The **E_2_‐hyd** model features a bridging hydride, stabilized by the open belt sulfide‐bridge while the **E_2_‐nonhyd** model contains no hydride but instead two protonated closed belt sulfide bridges. Clearly these two models should feature very different electronic structures.

**Figure 5:**
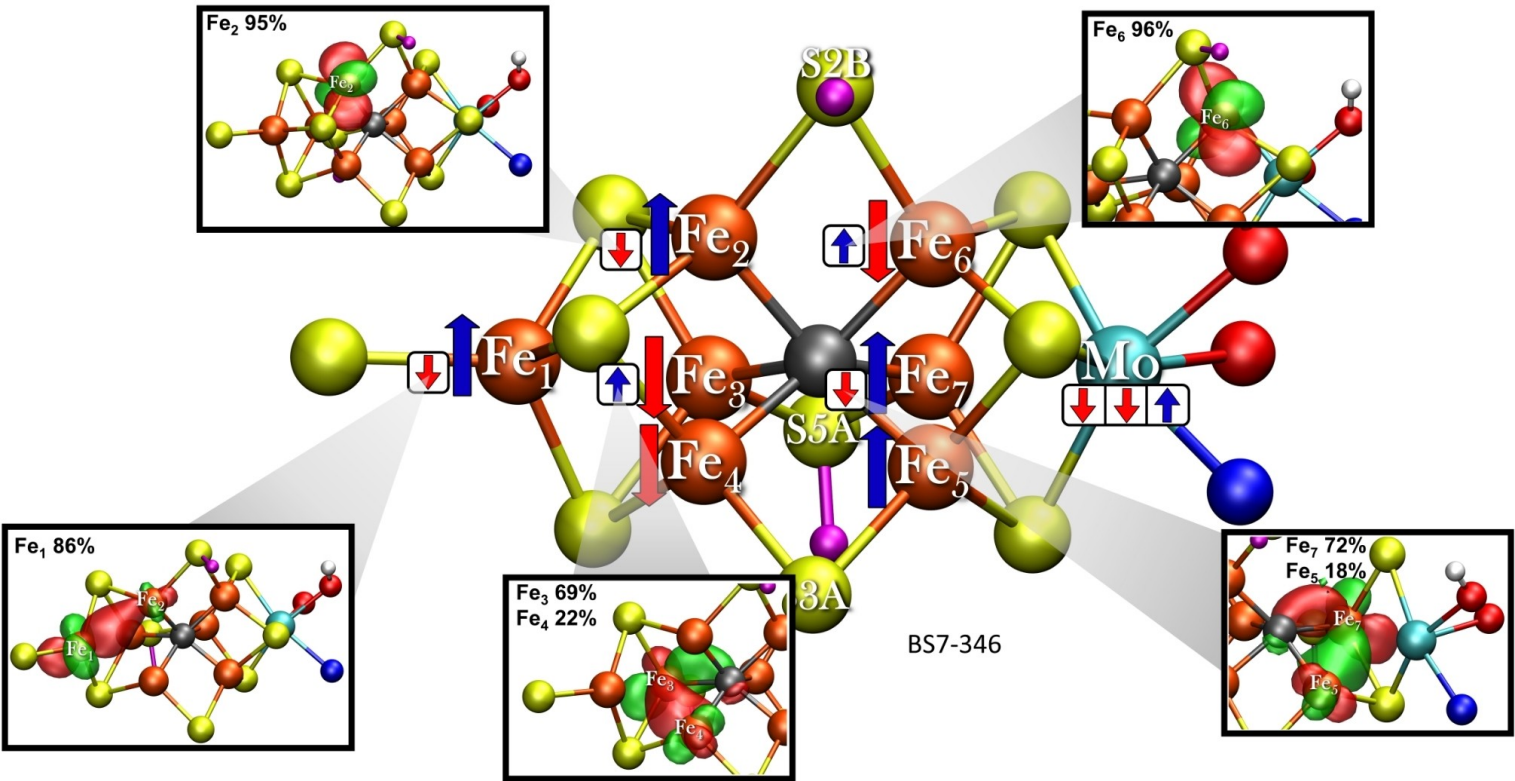
The electronic structure of the **E_2_‐nonhyd** model in the BS7‐346 solution as interpreted via localized orbital analysis and shown as Noodleman‐style majority/minority spin vectors. Localized orbital isosurfaces (0.05 isovalue) of the minority‐spin electrons are shown as insets. Large arrows indicate 5‐electron s=5/2 majority‐spin vectors, these 5 electrons are well localized (see Supporting Information) while small arrows indicate single s=1/2 electrons. The model shown has α‐195^His^ in the N_ϵ_ protonation state.

The **E_2_‐nonhyd** electronic structure features a second metal‐based reduction with respect to our model for the E_1_ redox state (where the added electron was found to be in the MoFe_3_S_3_C sub‐cubane).^[16]^ While a Mo‐based reduction is in principle possible, we found that an Fe‐based reduction occurs yet again according to a localized orbital analysis of the lowest‐energy broken‐symmetry state (BS7‐346) of the **E_2_‐nonhyd** model. This analysis is shown in Figure 5.

By utilizing localized orbital analysis on the BS7‐346 broken‐symmetry determinant the complex electronic structure of the 43 unpaired electrons of this [MoFe_7_S_9_CH_2_]^−^ cluster can be somewhat simplified as 5 electrons (majority‐spin) on each Fe ion (35 in total) are found to be well localized (90–100 % population for Fe ions in the Fe_4_S_3_C sub‐cubane and 80–100 % in the MoFe_3_S_3_C sub‐cubane) and can hence be portrayed as in Figure 5 with a single *up* or *down* majority spin vector. Such spin‐vector diagrams were popularized by Noodleman and coworkers to describe the electronic structure of iron‐sulfur clusters.^[75]^ The alignment of the majority‐spin vectors also define the label for the last 3 numbers of the broken‐symmetry solution BS7‐346: majority‐spin vectors on Fe_3_, Fe_4_ and Fe_6_ being spin‐down. The remaining 8 electrons deserve further attention. Three of them belong to the Mo(III) ion (although with some Fe contribution) and are curiously in an unusual non‐Hund configuration as previously described.^[10]^ As the Mo electron configuration remains mostly unperturbed in our E_2_ models compared to E_0_ and E_1_, we will not discuss the Mo electrons further here. The 5 remaining electrons are Fe‐based and are shown as minority‐spin vectors in Figure 5. These electrons can in principle be completely localized (resulting in a Fe(II) ion) or completely delocalized (resulting in a Fe(2.5)‐Fe(2.5) pair) or in between those two extremes. Three minority‐spin electrons were already present in the E_0_ redox state (two in the Fe_4_S_3_C subcubane and one in the MoFe_3_S_3_C subcubane) that also features BS7 class determinants as lowest in energy. Compared to E_0_ we have added two additional electrons to the cofactor; one additional electron into the MoFe_3_S_3_C subcubane (as previously described for E_1_ ^[16]^) and another electron into the Fe_4_S_3_C subcubane. Overall the electronic structure is found to be fairly localized, more so than E_0_ and slightly more than E_1_, with less mixed‐valence delocalization. The overall Fe redox state of the **E_2_‐nonhyd** BS7‐346 state is reasonably well described as 5Fe(II)2Fe(III) with the two Fe(III) ions located at Fe_4_ and Fe_5_ positions, being antiferromagnetically aligned. The position of the Fe(III)‐Fe(III) pair at Fe_4_ and Fe_5_ positions can be rationalized as the super‐exchange pathway should be stronger at this non‐protonated belt‐sulfide position leading to stronger antiferromagnetic coupling in the Fe(III)‐Fe(III) pair.

**Figure 6:**
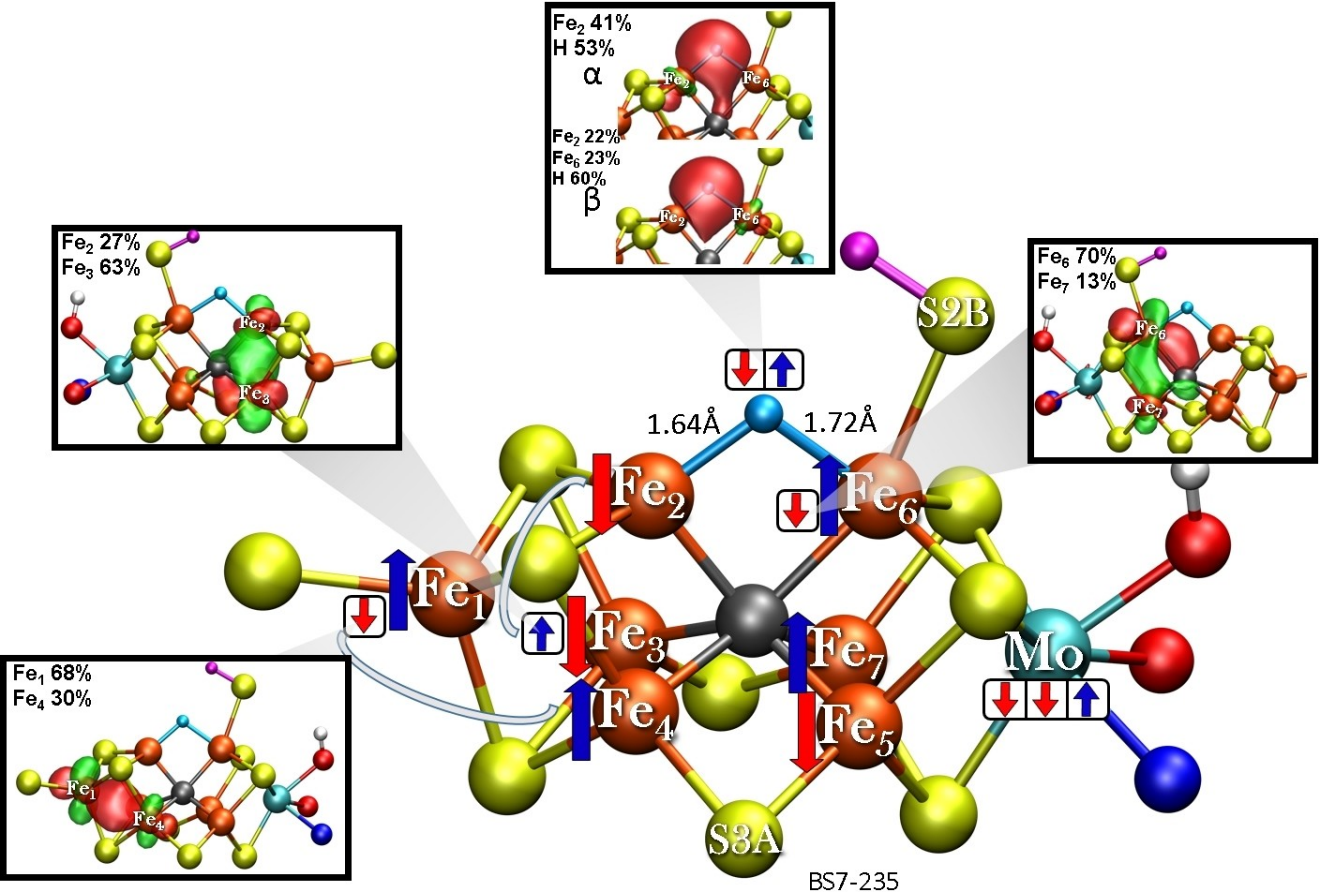
The electronic structure of **E_2_‐hyd** model in the BS7‐235 solution as interpreted via localized orbital analysis. Localized orbitals of the minority‐spin electrons are shown in inlays with contour value of 0.05. The model shown has α‐195^His^ in the N_ϵ_ protonation state.

The **E_2_‐hyd** model features a rather different electronic structure as shown in Figure 6. Instead of the two added electrons reducing the Fe part of FeMoco, the electrons instead go into the formation of the bridging hydride that then engages in a bridging σ‐donor interaction with Fe_2_ and Fe_6_. The rest of the electronic structure of **E_2_‐hyd** resembles more the E_0_ redox state, formally 3Fe(II)4Fe(III). Different to E_0_, however, the mixed‐valence pairs, Fe_2_‐Fe_3_ and Fe_6_‐Fe_7_, are not as delocalized as previously found[^12^, ^14^] with the minority‐spin electron in the Fe_6_‐Fe_7_ mixed‐valence pair localizing on the sulfhydryl‐bound Fe_6_ ion while the minority‐spin electron in the Fe_2_‐Fe_3_ pair localizes on Fe_3_. The bridging hydride in the **E_2_‐hyd** model shows some asymmetry, having a shorter Fe_2_‐H bond (1.64 Å) than an Fe_6_−H bond (1.72 Å).

### E A mechanism of H_2_ formation via hydride protonolysis

**Figure 7:**
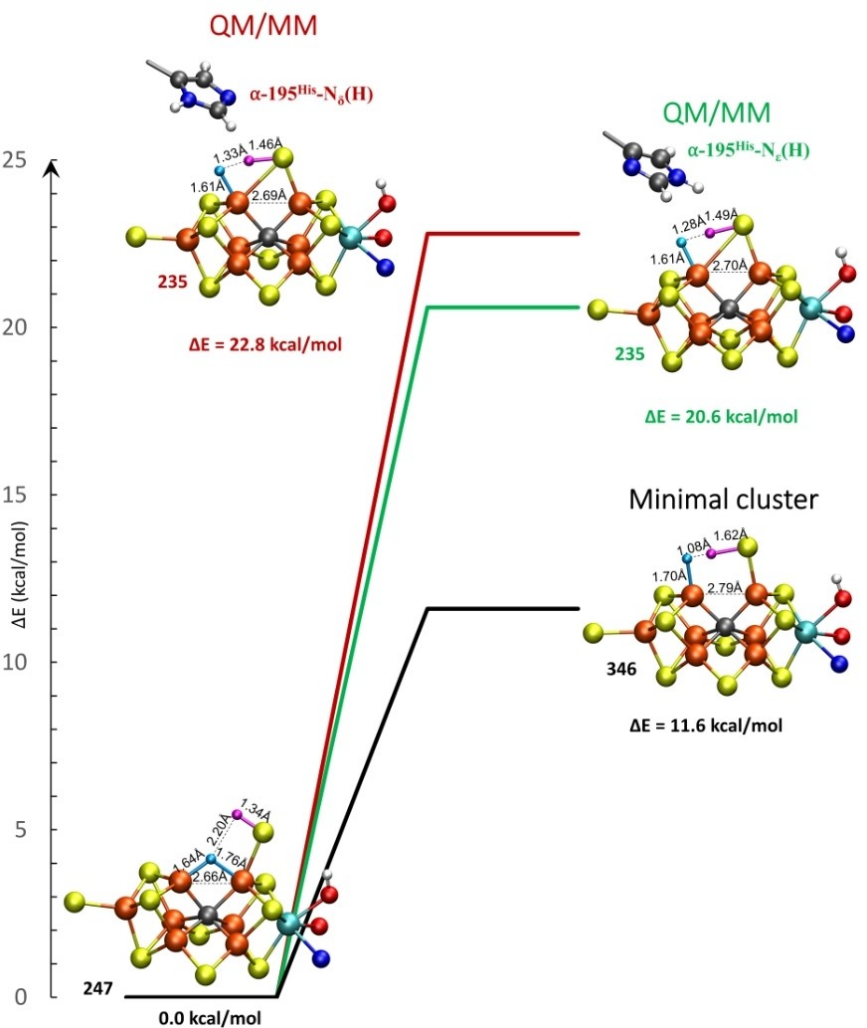
Saddle point geometries and activation barriers for H_2_ evolution from **E_2_‐hyd** (for each QM/MM and minimal‐cluster model, respectively) leading towards an **E_0_+H_2_** state. QM/MM models include α‐195^His^ in either the N_δ_(H) or N_ϵ_(H) protonation state while the residue is not included in the minimal cluster model. The **E_2_‐hyd** structure shown is the QM/MM α‐195^His^‐N_ϵ_(H), see Supporting Information for structural parameters for QM/MM α‐195^His^‐N_δ_(H) and the minimal cluster model. The BS7‐235 solution was used for all models.

The hydride model, **E_2_‐hyd**, features a bridging hydride between Fe_2_ and Fe_6_ and a proton on the terminal S2B in close proximity. Such a structure immediately suggests a possible mechanism for H_2_ evolution, via direct hydride and proton combination, leading to H_2_ formation and the reformation of the S2B sulfide bridge between Fe_2_ and Fe_6_, resulting in an E_0_ redox state of FeMoco. We note that we tried stabilizing a possible η^2^‐H_2_ intermediate that might form prior to H_2_ elimination but this was found not to occur. Instead, a saddle point on the minimum energy path towards H_2_ formation was located starting from the **E_2_‐hyd** model. These calculations were performed using the BS7‐235 solution with the α‐195^His^ residue in either the N_δ_ or N_ϵ_ protonation states for the QM/MM models or with the minimal cluster‐continuum model. The resulting saddle point geometries and activation barriers are compared in Figure 7.

The QM/MM calculations predict relatively high activation barriers of either 20.6 kcal/mol (α‐195^His^‐N_ϵ_(H)) or 22.8 kcal/mol (α‐195^His^‐N_δ_(H)). The slightly lower energy barrier for the α‐195^His^‐N_ϵ_(H) model is likely due to a stabilizing α‐195^His^‐N_ϵ_‐H⋯S2B hydrogen‐bond. Interestingly, the barrier is much lower, 11.6 kcal/mol, when calculated using the minimal cluster model, where the protein environment (including α‐195^His^) is absent. This lower barrier seemingly arises due to a more favorable saddle point geometry in the minimal cluster model where the hydride is closer to the sulfide‐proton, resulting in a ‘later’ transition state. This may be enabled by increased flexibility in the minimal cluster model, allowing an increased Fe_2_‐Fe_6_ distance, seemingly not possible in the QM/MM model, though we note that it's also possible that the protein environment might be capable of rearrangement to further reduce the barrier, something not captured by our current computational model (which is biased towards the resting state E_0_ structure). Zero‐point vibrational corrections to the barrier were calculated (via QM/MM partial Hessians) for the α‐195^His^‐N_ϵ_(H) form. This reduces the activation barrier from 20.6 kcal/mol to 18.5 kcal/mol. The zero‐point vibrational energy also allows one to calculate the kinetic isotope effect (KIE) for H/D substitution for H_2_ formation. The KIE was calculated to be 3.21 which is in good agreement with the experimental KIE estimates (2.7 at 298 K, ∼3 at 243 K).^[27]^

The saddle point geometries reveal partial H−H bond formation between the hydride (in a more terminal geometry) and the sulfide‐proton at the same time as the sulfide bridge reforms between Fe_2_ and Fe_6_. While the restoration of the sulfide bridge should increase the driving force of the reaction, we speculate that such a complex saddle point requiring simultaneous H−H bond, Fe−S bond formation and sulfide deprotonation is likely rather unfavorable and increases the barrier height for this otherwise simple hydride‐proton combination reaction. Furthermore, the lowered barrier height in the calculation without an explicit protein environment suggests that the protein environment even disfavors H_2_ formation. As the H_2_ evolution of nitrogenase via the E_2_→E_0_ pathway is a non‐productive side‐reaction (resulting in the loss of reducing equivalents for dinitrogen reduction), the stability of a bridging‐hydride structure with an open sulfide bridge which in turn results in a higher activation barrier for H_2_ formation may thus be beneficial for the enzyme in slowing down the H_2_ relaxation and allowing build‐up of more reduced states E_3_ and E_4_, to which N_2_ will eventually bind.

We note that in the study by Khadka et al. the authors also located a saddle point for H_2_ formation, starting from a **bH(2,6)CBS(S2B)** model, using a minimal cluster‐continuum model approach (where the homocitrate was modelled as glycolate) and the BP86 functional. Such a closed sulfide‐bridge hydride model is predicted to be rather high in energy (∼9 kcal/mol) compared to our favored **E_2_‐hyd** model (**bH(2,6)‐OBS(6)**) at our TPSSh‐QM/MM level of theory. Attempts to locate a similar closed‐bridge saddle point were not successful (calculations always converged to open sulfide‐bridge structures), likely due to the different representation of the protein environment and the different theory level.

**Figure 8:**
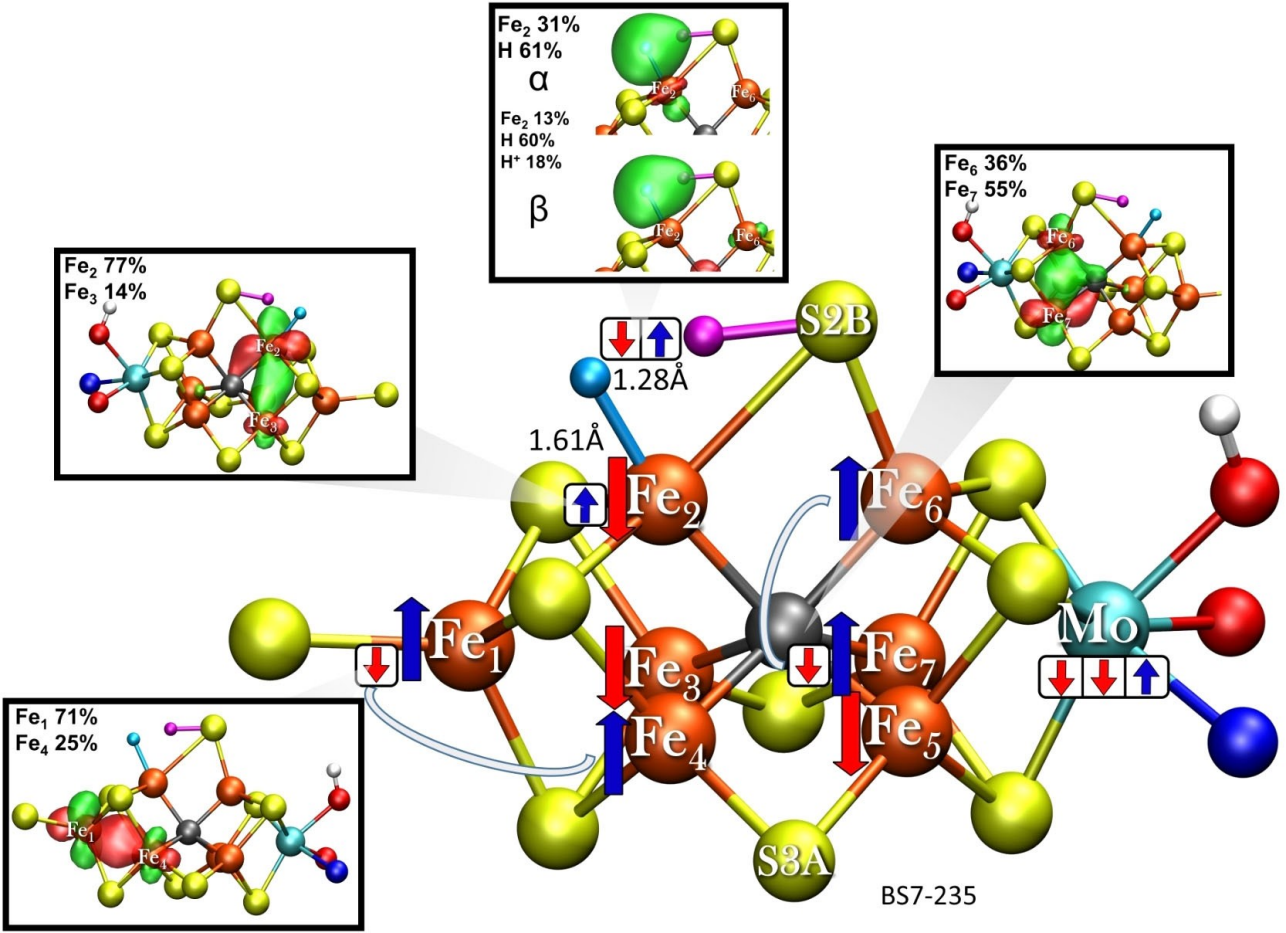
The electronic structure of the saddle point structure (**E_2_‐hyd‐TS**) for H_2_ formation. The model shown has α‐195^His^ in the N_ϵ_ protonation state.

A localized orbital analysis of the H_2_ formation saddle point in shown in Figure 8. The Fe electronic structure of the cofactor is overall rather localized compared to the E_0_ state and interestingly the minority‐spin electron between Fe_2_ and Fe_3_ is localized on Fe_2_ instead of Fe_3_ as in the **E_2_‐hyd** structure in Figure 6 (in the analogous E_0_ BS‐state the electron is more delocalized^[14]^). Similarly for the Fe_6_‐Fe_7_ pair, the minority‐spin electron instead shows more localization on Fe_7_. The localization of the electron at Fe_2_ likely contributes to H−H bond formation by increasing the hydricity. The ability of the minority‐spin electrons in the mixed‐valence pairs of FeMoco to delocalize or localize depending on the situation as seen here, may be behind some of the interesting reactivity that this cofactor exhibits.

## Conclusions

We have presented a detailed exploration of the potential energy surface of the E_2_ redox state of FeMoco in terms of hydride and non‐hydride isomers while accounting for multiple broken‐symmetry states for each model with a detailed explicit treatment of the protein environment. We demonstrate that for hydride models there is a strong thermodynamic preference for opening up the protonated belt sulfide bridge, that is, to convert a bridging, μ‐SH group to a terminal SH group on one of the Fe atoms. This appears to stabilize the bridging hydride, similarly to what we previously found for bridging hydrides of E_4_ models.^[21]^ Furthermore, the protein environment stabilizes one particular hydride isomer, where the hydride bridges atoms Fe_2_ and Fe_6_ and with a terminal sulfhydryl group residing on Fe_6_.

Surprisingly, however, it is as energetically favorable to not form a hydride at all, as in the **E_2_‐nonhyd** model where 2 electrons have reduced the Fe ions of the cofactor and 2 H^+^ are present on sulfides S2B and S5A. We note in this context that EPR studies under turnover conditions have revealed two distinct S=3/2 signals (known as 1b and 1c) with notably different g‐tensors (1b g=[4.21,3.76,?], 1c (g=[4.69,∼3.20,?]) and with an estimated energy difference of ∼1–2 kcal/mol (1b being more stable).^[9]^ The 1c state has furthermore been found to resemble a low‐pH protonated (presumably belt sulfide protonated) resting state signal E_0_(H^+^) state (g=[4.71,3.30,2.01]).^[76]^ It seems not inconceivable that these two distinct EPR signals, 1b and 1c, could correspond to the **E_2_‐hyd** and **E_2_‐nonhyd** models suggested in this work. The study by Lukoyanov et al.^[26]^ demonstrated that the state with the 1b EPR signal likely corresponds to a hydride state at the E_2_ redox level based on the known photoreactivity of metal hydrides. It was furthermore shown by photolysis of 1b that a photogenerated version of 1c (1c*) can form that was suggested to be a different hydride isomer. While a non‐hydride isomer for 1c was deemed unlikely by the authors of the study since a non‐hydride E_2_ isomer had been previously captured by photolysis of the E_4_ state (S=1/2, g=[2.098, 2.000, 1.956]), this may simply mean that multiple E_2_ photoproducts may form in these experiments.

This attempted connection between our models to the results of the EPR and photolysis experiments is of course rather speculative and a clearer connection between spectroscopy and theory is needed to establish the precise nature of the E_2_ redox state under turnover conditions. Future calculations of EPR g‐tensors of QM/MM models may be one possibility; however, this is highly challenging, even for mononuclear complexes.[^77^, ^78^] We note that the models we suggest have distinct molecular and electronic structures that should be spectroscopically distinguishable, if experimental problems associated with low accumulation of reduced FeMoco states can be overcome or if belt Fe‐selective ^57^Fe isotope labelling of FeMoco becomes possible.^[79]^ Apart from ENDOR, direct detection of a hydride (or possibly the terminal SH group) through, for example, ^57^Fe nuclear resonance vibrational spectroscopy (as has recently been successfully applied to hydrogenase enzymes[^80^, ^81^, ^82^, ^83^, ^84^]) could become possible and a spectroscopic experiment sensitive to Fe oxidation state (e. g., Mössbauer or Fe X‐ray absorption) should in principle allow a clear distinction between **E_2_‐hyd** and **E_2_‐nonhyd** models.

**Figure 9:**
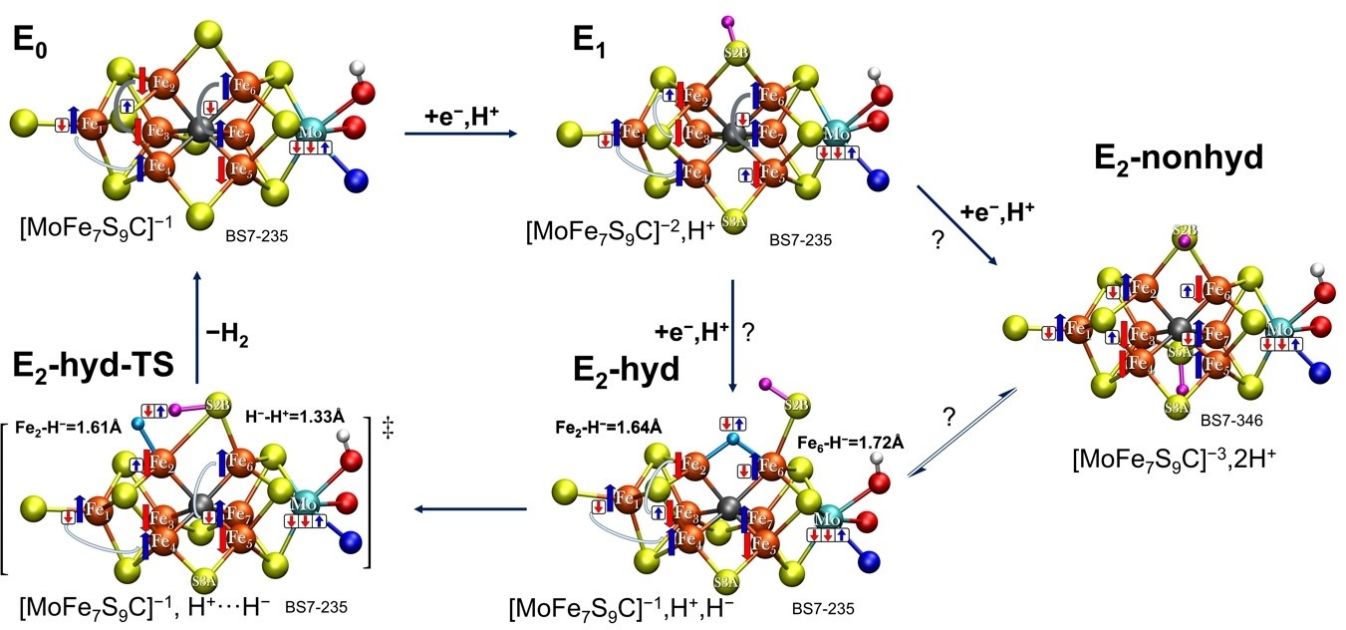
A preliminary **E_0_** →**E_1_** →**E_2_** →**E_0_** redox cycle proposal of FeMoco based on the QM/MM models from this work and previous work.[^14^, ^16^, ^21^] The BS7‐235 solution (Fe_2_,Fe_3_,Fe_5_ spin‐down) is shown for simplicity for all models (except **E_2_‐nonhyd** where BS7‐346 is more strongly preferred) as this solution is consistently one of the lowest BS determinants found for **E_0_**, **E_1_**, **E_2_‐hyd** and **E_2_‐hyd‐TS**. It is not clear whether **E_2_‐nonhyd** or **E_2_‐hyd** would form first upon e^−^/H^+^ transfer to the **E_1_** state, though we note that the **E_2_‐nonhyd** model is structurally more similar to the model for the **E_1_** state.

Our preliminary proposal for an **E_0_** →**E_1_** →**E_2_** →**E_0_** redox cycle is shown in Figure 9. This redox cycle relates our electronic structure interpretation of **E_0_** (based on localized orbitals of the BS7‐235 solution) with a fairly delocalized electronic structure, to our favored model of the 1‐electron reduced **E_1_** state (with a reduced Fe in the MoFe_3_S_3_C sub‐cubane and a protonated S2B belt sulfide) that exhibits a slightly more localized electronic structure. From the Fe‐reduced, S2B‐protonated **E_1_** model one can reduce the Fe ions again, while protonating S5A, which leads to the **E_2_‐nonhyd** model with an even more localized electronic structure, probably best described as 5Fe(II)2Fe(III). Alternatively, the **E_2_‐hyd** state can form where the electrons are instead stored as part of the bridging hydride with the rest of the cofactor remaining rather similar to the E_0_ state (albeit showing less delocalization). From the **E_2_‐hyd** model a direct path towards H_2_ evolution is easily imagined and we were able to locate a saddlepoint (**E_2_‐hyd‐TS**) for H_2_ formation and relaxation of the cofactor back to the **E_0_** state.

The **E_2_‐nonhyd** model is geometrically much closer to the proposed **E_1_(S2B)** model (requiring only Fe reduction and proton transfer to S5A) and hence it seems possible that upon reduction of the E_1_ state, this non‐hydridic E_2_ state could form first, before relaxing to the slightly more stable hydride species, **E_2_‐hyd** (and relaxing back to E_0_ via H_2_ evolution). Details about the nature of electron/proton transfer pathways in nitrogenase are scarce, however. With these two E_2_ models being close in energy this further begs the question whether an E_3_ state might initially form from a hydridic E_2_ state or a non‐hydridic E_2_ state. Formation of E_3_ from the non‐hydridic state, however, seems unlikely as both the Fe sub‐cubane and the Mo sub‐cubane are in their reduced forms. With the two electrons being primarily localized on the hydride in **E_2_‐hyd** (likely to apply to other hydride models as well) this should make reduction of the Mo sub‐cubane a more likely reduction to reach the EPR‐silent E_3_ state.

## Supporting Information Available:

Relative energies and Mulliken spin populations of all BS states calculated for each E_2_ model using the QM/MM or minimal‐cluster model approach. Bond lengths, bond orders and Hirshfeld charges of selected E_2_ models. Localized orbital analysis for E_0_, E_1_ and E_2_ models. Information on hydrogen orientations for E_2_ models. Cartesian coordinates for all optimized structures.

## Conflict of interest

The authors declare no conflict of interest.

## Supporting information

As a service to our authors and readers, this journal provides supporting information supplied by the authors. Such materials are peer reviewed and may be re‐organized for online delivery, but are not copy‐edited or typeset. Technical support issues arising from supporting information (other than missing files) should be addressed to the authors.

Supporting Information

Supporting Information
