# Supplementary material for: The E2 state of FeMoco: Hydride Formation versus Fe Reduction and a Mechanism for H2 Evolution
Source: Chemistry. 2021 Oct 15;27(67):16788–800. doi: 10.1002/chem.202102730 (PMC9293435; doi:10.1002/chem.202102730)
Supplement: Supplementary file 1 — Supporting Information [file CHEM-27-16788-s002.pdf]

Chemistry–A European Journal

Supporting Information

# The $E_2$ state of FeMoco: Hydride Formation versus Fe Reduction and a Mechanism for $H_2$ Evolution

Albert Th. Thorhallsson and Ragnar Bjornsson*

## Coordinates of $E_2$ models

Cartesian coordinates of the QM region for all QM/MM-optimized $E_2$ models are available as XYZ files in a compressed archive.

**Table S1.** Calculated relative energies in kcal/mol (TPSSh level of theory using the minimal cluster model) and Mulliken spin populations on the metal ions for all 4 broken-symmetry solutions (235, 346, 247, 147—if found) of the $E_2$ models shown in Fig. 3 of article.

| Model | QM | Mo | $Fe_1$ | $Fe_2$ | $Fe_3$ | $Fe_4$ | $Fe_5$ | $Fe_6$ | $Fe_7$ |
|---|---|---|---|---|---|---|---|---|---|
| **noH-CBS(S2B,S3A)-147** | 21.31 | -0.05 | -3.25 | 3.31 | 3.28 | -0.54 | 2.61 | 0.95 | -3.13 |
| **noH-CBS(S2B,S3A)-235** | 6.15 | -0.83 | 3.51 | -3.20 | -3.32 | 3.32 | -2.54 | 2.52 | 3.07 |
| **noH-CBS(S2B,S3A)-247** | 5.73 | -0.43 | 3.46 | -3.07 | 3.40 | -2.97 | 2.79 | 2.56 | -3.13 |
| **noH-CBS(S2B,S3A)-346** | 3.03 | -0.32 | 3.45 | 3.02 | -3.37 | -3.28 | 2.93 | -2.64 | 3.05 |
| **noH-CBS(S2B,S5A)-147** | 26.43 | 0.39 | -1.84 | 3.31 | 3.36 | -2.90 | 3.12 | -1.92 | -1.39 |
| **noH-CBS(S2B,S5A)-235** | 6.24 | -0.59 | 3.47 | -3.22 | -3.29 | 3.41 | -2.69 | 2.59 | 2.93 |
| **noH-CBS(S2B,S5A)-247** | 1.77 | -0.40 | 3.44 | -3.29 | 3.05 | -3.36 | 3.06 | 2.95 | -2.61 |
| **noH-CBS(S2B,S5A)-346** | 1.06 | -0.32 | 3.47 | 3.06 | -3.34 | -3.35 | 3.01 | -2.64 | 3.00 |
| **noH-CBS(S3A,S5A)-147** | 10.29 | -0.36 | -3.50 | 3.28 | 3.29 | -3.27 | 2.93 | 3.13 | -2.55 |
| **noH-CBS(S3A,S5A)-235** | 3.20 | -0.63 | 3.46 | -3.34 | -3.32 | 2.99 | -2.52 | 3.08 | 3.00 |
| **noH-CBS(S3A,S5A)-247** | 1.78 | -0.43 | 3.44 | -3.35 | 3.05 | -3.30 | 2.99 | 3.04 | -2.61 |
| **noH-CBS(S3A,S5A)-346** | 6.79 | -0.40 | 3.48 | 3.39 | -3.09 | -3.00 | 2.79 | -3.10 | 2.58 |
| **bH(4,5)-OBS(5)-147** | 20.64 | -0.21 | -3.39 | 3.20 | 3.24 | -1.65 | 2.86 | 2.28 | -3.13 |
| **bH(4,5)-OBS(5)-235** | 12.30 | 0.71 | -3.27 | 3.38 | 3.22 | 3.02 | 2.50 | -3.10 | -3.14 |
| **bH(4,5)-OBS(5)-247** | 0.00 | -0.39 | 3.48 | -3.26 | 3.36 | -3.23 | 2.94 | 2.94 | -3.11 |
| **bH(4,5)-OBS(5)-247** | 33.70 | 0.32 | 3.62 | -2.37 | 3.63 | -1.85 | -2.56 | -2.27 | 3.05 |
| **bH(4,5)-OBS(5)-346** | 0.38 | -0.39 | 3.49 | 3.34 | -3.28 | -3.23 | 2.94 | -3.12 | 2.95 |
| **bH(2,6)-OBS(6)-147** | 4.85 | -0.46 | -3.46 | 3.47 | 3.35 | -3.22 | 3.12 | 3.26 | -3.05 |
| **bH(2,6)-OBS(6)-235** | 1.82 | -0.60 | 3.51 | -3.24 | -3.28 | 3.35 | -3.06 | 3.01 | 2.96 |
| **bH(2,6)-OBS(6)-346** | 3.52 | -0.49 | 3.49 | 3.34 | -3.29 | -3.26 | 3.00 | -3.28 | 3.02 |
| **bH(3,7)-OBS(7)-147** | 9.28 | -0.41 | -3.51 | 3.44 | 3.26 | -3.31 | 3.08 | 3.18 | -2.97 |
| **bH(3,7)-OBS(7)-235** | 0.64 | -0.63 | 3.54 | -3.29 | -3.27 | 3.37 | -3.05 | 2.98 | 2.98 |
| **bH(3,7)-OBS(7)-247** | 3.92 | -0.46 | 3.49 | -3.27 | 3.38 | -3.27 | 3.01 | 2.97 | -3.24 |
| **bH(3,7)-OBS(7)-346** | 1.42 | -0.36 | 3.54 | 3.37 | -3.27 | -3.28 | 2.93 | -3.11 | 2.96 |
| **bH(2,6)-OBS(2)-147** | 19.79 | -0.50 | -2.19 | 2.87 | 3.34 | -3.14 | 2.97 | 2.75 | -3.09 |
| **bH(2,6)-OBS(2)-235** | 4.07 | -0.59 | 3.50 | -3.23 | -3.31 | 3.35 | -3.05 | 2.96 | 2.99 |
| **bH(2,6)-OBS(2)-247** | 3.74 | -0.43 | 3.48 | -3.23 | 3.37 | -3.29 | 2.99 | 2.89 | -3.09 |
| **bH(2,6)-OBS(2)-346** | 6.05 | -0.40 | 3.52 | 3.40 | -3.28 | -3.28 | 2.92 | -3.11 | 2.93 |
| **bH(3,7)-OBS(3)-147** | 8.19 | -0.54 | -3.52 | 3.49 | 3.32 | -3.38 | 3.08 | 3.19 | -2.79 |
| **bH(3,7)-OBS(3)-235** | 1.96 | -0.56 | 3.53 | -3.30 | -3.25 | 3.38 | -3.06 | 2.99 | 2.91 |
| **bH(3,7)-OBS(3)-247** | 3.68 | -0.45 | 3.57 | -3.31 | 3.38 | -3.30 | 2.96 | 2.92 | -3.10 |
| **bH(3,7)-OBS(3)-346** | 2.42 | -0.35 | 3.53 | 3.38 | -3.25 | -3.30 | 2.96 | -3.11 | 2.89 |
| **bH(4,5)-OBS(4)-147** | 11.19 | -0.50 | -3.50 | 3.45 | 3.35 | -3.31 | 3.05 | 3.18 | -2.88 |
| **bH(4,5)-OBS(4)-235** | 6.07 | -0.63 | 3.51 | -3.29 | -3.28 | 3.39 | -3.06 | 2.97 | 2.97 |
| **bH(4,5)-OBS(4)-247** | 20.00 | 0.45 | 3.40 | 3.22 | -3.41 | 2.32 | -3.05 | -3.02 | 3.01 |
| **bH(4,5)-OBS(4)-346** | 5.44 | -0.38 | 3.49 | 3.35 | -3.31 | -3.21 | 2.90 | -3.10 | 2.99 |
| **bH(2,6)-CBS(S2B)-147** | 20.20 | -0.43 | -2.14 | 3.02 | 3.35 | -3.16 | 3.07 | 2.25 | -2.94 |
| **bH(2,6)-CBS(S2B)-247** | 8.22 | -0.52 | 3.49 | -3.15 | 3.38 | -3.26 | 2.96 | 2.83 | -3.15 |
| **bH(2,6)-CBS(S2B)-346** | 9.83 | -0.30 | 3.49 | 3.21 | -3.30 | -3.27 | 2.95 | -3.03 | 2.97 |
| **bH(4,5)-CBS(S3A)-235** | 8.75 | -0.54 | 3.48 | -3.28 | -3.29 | 3.23 | -2.96 | 2.99 | 3.00 |
| **bH(4,5)-CBS(S3A)-247** | 8.62 | -0.42 | 3.50 | 3.37 | -3.29 | -3.15 | 2.76 | -3.16 | 2.96 |
| **bH(3,7)-CBS(S5A)-235** | 6.90 | -0.47 | 3.52 | -3.30 | -3.29 | 3.41 | -2.57 | 3.14 | 2.12 |
| **bH(3,7)-CBS(S5A)-247** | 8.68 | -0.67 | 3.48 | -3.52 | 3.04 | -3.51 | 2.89 | 2.91 | -1.59 |
| **bH(3,7)-CBS(S5A)-247** | 3.19 | -0.14 | 3.52 | 3.46 | -3.25 | -3.24 | 3.03 | -2.73 | 1.86 |
| **bH(4,5)-CBS(S3A)-235** | 8.75 | -0.54 | 3.48 | -3.28 | -3.29 | 3.23 | -2.96 | 2.99 | 3.00 |
| **bH(4,5)-CBS(S3A)-346** | 8.62 | -0.42 | 3.50 | 3.37 | -3.29 | -3.15 | 2.76 | -3.16 | 2.96 |
| **bH(3,7)-CBS(S2B)-235** | 6.90 | -0.47 | 3.52 | -3.30 | -3.29 | 3.41 | -2.57 | 3.14 | 2.12 |
| **bH(3,7)-CBS(S2B)-247** | 8.68 | -0.67 | 3.48 | -3.52 | 3.04 | -3.51 | 2.89 | 2.91 | -1.59 |
| **bH(3,7)-CBS(S2B)-346** | 3.19 | -0.14 | 3.52 | 3.46 | -3.25 | -3.24 | 3.03 | -2.73 | 1.86 |

| | | | | | | | | | |
|---|---|---|---|---|---|---|---|---|---|
| **tH(2)-CBS(S2B)-235** | 17.72 | -0.55 | 3.19 | -2.81 | -3.32 | 3.26 | -3.01 | 3.05 | 3.03 |
| **tH(2)-CBS(S2B)-247** | 12.72 | -0.37 | 3.43 | -2.72 | 3.07 | -3.34 | 2.94 | 2.94 | -3.08 |
| **tH(2)-CBS(S2B)-346** | 10.81 | -0.37 | 3.54 | 2.88 | -3.27 | -3.23 | 2.96 | -2.67 | 2.97 |
| **tH(3)-CBS(S2B)-147** | 16.99 | -0.48 | -3.44 | 3.42 | 2.83 | -3.16 | 3.08 | 3.14 | -2.45 |
| **tH(3)-CBS(S2B)-235** | 17.54 | -0.52 | 3.16 | -3.28 | -2.86 | 3.28 | -2.95 | 2.98 | 3.05 |
| **tH(3)-CBS(S2B)-247** | 11.01 | -0.41 | 3.48 | -3.26 | 3.02 | -3.27 | 3.01 | 2.89 | -2.63 |
| **tH(3)-CBS(S2B)-346** | 11.86 | -0.39 | 3.47 | 2.90 | -2.80 | -3.32 | 2.89 | -2.65 | 2.68 |
| **tH(4)-CBS(S2B)-147** | 17.11 | -0.45 | -3.50 | 3.12 | 3.34 | -2.88 | 3.07 | 3.00 | -2.89 |
| **tH(4)-CBS(S2B)-235** | 11.38 | -0.57 | 3.50 | -3.24 | -3.29 | 2.99 | -2.53 | 2.90 | 3.03 |
| **tH(4)-CBS(S2B)-247** | 11.38 | -0.44 | 3.46 | -3.26 | 3.41 | -2.73 | 2.91 | 2.39 | -3.10 |
| **tH(4)-CBS(S2B)-346** | 12.22 | -0.39 | 3.46 | 2.93 | -3.36 | -2.77 | 2.62 | -2.64 | 2.94 |
| **tH(5)-CBS(S2B)-147** | 12.65 | -0.27 | -3.49 | 3.41 | 3.41 | -3.06 | 2.43 | 3.11 | -2.72 |
| **tH(5)-CBS(S2B)-235** | 14.66 | -0.61 | 3.45 | -3.20 | -3.29 | 2.92 | -2.59 | 2.99 | 3.01 |
| **tH(5)-CBS(S2B)-247** | 9.65 | -0.26 | 3.45 | -3.24 | 3.37 | -3.27 | 2.44 | 2.99 | -2.73 |
| **tH(5)-CBS(S2B)-346** | 7.00 | -0.34 | 3.50 | 3.25 | -3.29 | -3.27 | 2.31 | -2.60 | 3.08 |
| **tH(6)-CBS(S2B)-147** | 13.50 | -0.26 | -3.42 | 3.39 | 3.37 | -3.04 | 3.12 | 2.32 | -2.72 |
| **tH(6)-CBS(S2B)-235** | 8.93 | -0.43 | 3.47 | -3.26 | -3.26 | 3.37 | -2.60 | 2.26 | 3.07 |
| **tH(6)-CBS(S2B)-247** | 7.65 | -0.28 | 3.45 | -3.25 | 3.38 | -3.25 | 3.06 | 2.23 | -2.65 |
| **tH(6)-CBS(S2B)-346** | 10.27 | -0.69 | 3.50 | 2.95 | -3.49 | -3.46 | 2.91 | -1.75 | 2.94 |
| **tH(7)-CBS(S2B)-147** | 10.90 | -0.44 | 3.47 | -3.23 | -3.30 | 3.35 | -2.62 | 3.00 | 2.48 |
| **tH(7)-CBS(S2B)-235** | 10.92 | -0.44 | 3.47 | -3.23 | -3.30 | 3.35 | -2.62 | 2.99 | 2.48 |
| **tH(7)-CBS(S2B)-247** | 14.93 | -0.45 | 3.44 | -3.22 | 2.93 | -3.27 | 2.99 | 2.96 | -2.61 |
| **tH(7)-CBS(S2B)-346** | 7.61 | -0.29 | 3.50 | 3.25 | -3.31 | -3.25 | 3.02 | -2.61 | 2.34 |
| **tH(3)endo-CBS(S2B)-147**[a] | 17.00 | -0.49 | -3.44 | 3.42 | 2.83 | -3.17 | 3.08 | 3.14 | -2.44 |
| **tH(3)endo-CBS(S2B)-235**[a] | 14.27 | -0.49 | 3.43 | -3.28 | -2.82 | 3.08 | -2.98 | 2.92 | 2.96 |
| **tH(3)endo-CBS(S2B)-247**[a] | 11.03 | -0.41 | 3.48 | -3.25 | 3.01 | -3.27 | 3.02 | 2.89 | -2.63 |
| **tH(3)endo-CBS(S2B)-346**[a] | 18.46 | -0.32 | -3.41 | 3.28 | -2.69 | 3.25 | 2.13 | -2.58 | 2.73 |
| **tH(4)endo-CBS(S2B)-247**[a] | 32.31 | 0.22 | 3.43 | -1.76 | 3.50 | -2.79 | -2.39 | -1.79 | 3.23 |
| **tH(5)endo-CBS(S2B)-346**[a] | 13.68 | -0.26 | 3.52 | 3.36 | -3.27 | -3.26 | 2.18 | -2.65 | 3.05 |
| **tH(7)endo-CBS(S2B)-147**[a] | 19.03 | -0.55 | -3.51 | 2.99 | 3.30 | -3.41 | 2.98 | 3.04 | -1.87 |
| **tH(7)endo-CBS(S2B)-235**[a] | 16.13 | -0.50 | 3.51 | -3.24 | -3.30 | 3.42 | -2.57 | 3.02 | 2.47 |
| **tH(7)endo-CBS(S2B)-247**[a] | 18.93 | -0.38 | 3.44 | -3.29 | 3.10 | -3.35 | 2.95 | 2.88 | -2.52 |
| **tH(7)endo-CBS(S2B)-346**[a] | 23.81 | -0.49 | -3.45 | 3.44 | -3.19 | 3.38 | -2.48 | 3.21 | 2.58 |

[a]Endo refers to hydrides with an acute angle with respect to the central carbide while not bridging

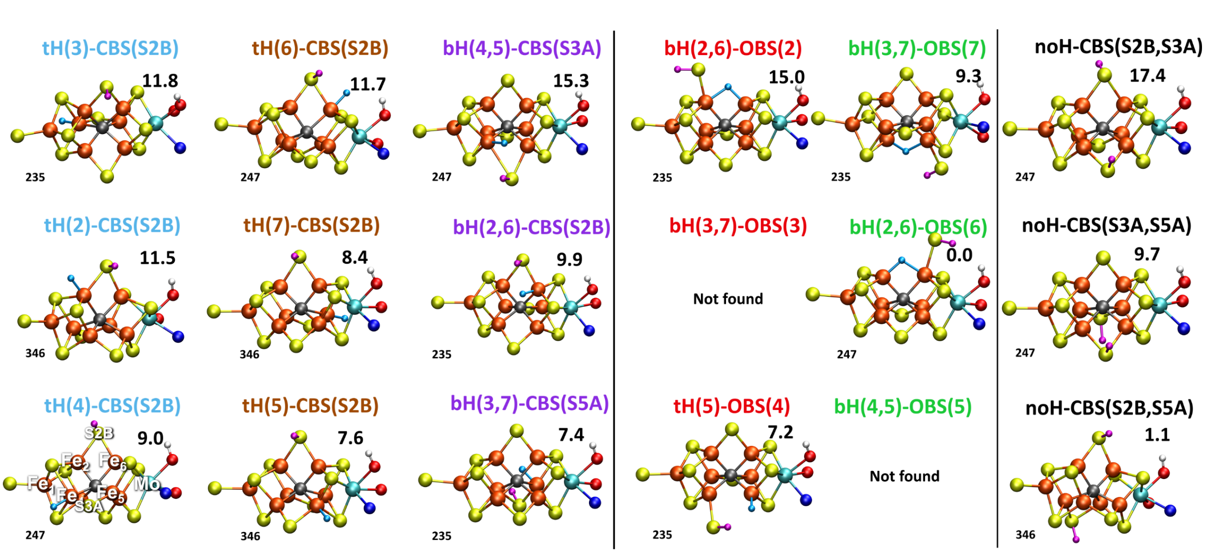


**Figure S1**. Calculated relative energies (polarized QM energy from QM/MM calculation) of different $E_2$ models in the $\alpha$-195$^{His}$-$N_\varepsilon$(H) protonation state. All models were calculated with 4 different broken-symmetry solutions with the lowest-energy one indicated. See Table S2 for other BS solutions.

**Table S2.** Calculated relative energies in kcal/mol (TPSSh, both polarized QM and QM/MM energies are shown) and Mulliken spin populations on the metal ions for all 4 broken-symmetry solutions (235, 346, 247, 147—if available) of $E_2$ models in the α-$195^{His}$-$N_\varepsilon$(H) protonation state in Fig. S1 using the QM/MM approach.

| Model | QM | QM/MM | Mo | $Fe_1$ | $Fe_2$ | $Fe_3$ | $Fe_4$ | $Fe_5$ | $Fe_6$ | $Fe_7$ |
|---|---|---|---|---|---|---|---|---|---|---|
| **noH-CBS(S3A,S5A)-247** | 9.70 | 11.51 | -0.34 | 3.36 | -3.27 | 2.99 | -3.16 | 2.91 | 3.00 | -2.58 |
| **noH-CBS(S2B,S3A)-247** | 17.44 | 16.35 | -0.31 | 3.35 | -3.23 | 3.13 | -3.14 | 2.91 | 2.97 | -2.65 |
| **noH-CBS(S2B,S5A)-247** | 3.85 | 3.32 | -0.41 | 3.36 | -3.21 | 2.95 | -3.28 | 3.10 | 2.91 | -2.51 |
| **noH-CBS(S2Bt3,S5A)-235**[a] | 8.12 | 6.90 | -0.33 | 3.11 | -3.23 | -3.19 | 3.26 | -2.78 | 3.02 | 2.97 |
| **noH-CBS(S2Bt3,S5A)-247**[a] | 2.18 | 1.28 | -0.37 | 3.36 | -3.20 | 2.95 | -3.27 | 3.08 | 2.87 | -2.52 |
| **noH-CBS(S2Bt3,S5A)-346**[a] | 1.12 | -0.06 | -0.24 | 3.36 | 2.98 | -3.21 | -3.29 | 3.07 | -2.55 | 2.86 |
| **bH(2,6)-OBS(6)-235** | 2.52 | 0.27 | -0.57 | 3.43 | -3.14 | -3.19 | 3.29 | -3.11 | 2.89 | 2.87 |
| **bH(2,6)-OBS(6)-247** | 1.42 | 1.36 | -0.46 | 3.43 | -3.15 | 3.32 | -3.16 | 2.98 | 2.86 | -3.02 |
| **bH(2,6)-OBS(6)-247**[b] | 0.00 | 0.00 | -0.48 | 3.42 | -3.15 | 3.31 | -3.16 | 2.98 | 2.90 | -3.02 |
| **bH(3,7)-OBS(7)-147** | 16.55 | 10.46 | -0.54 | -3.42 | 3.35 | 3.13 | -3.26 | 3.12 | 3.18 | -2.72 |
| **bH(3,7)-OBS(7)-235** | 9.28 | 3.26 | -0.47 | 3.45 | -3.21 | -3.15 | 3.32 | -3.09 | 2.98 | 2.74 |
| **bH(3,7)-OBS(7)-247** | 12.75 | 8.71 | -0.56 | 3.40 | -3.22 | 3.26 | -3.19 | 3.04 | 2.99 | -3.06 |
| **bH(3,7)-OBS(7)-346** | 10.11 | 2.84 | -0.21 | 3.42 | 3.34 | -3.15 | -3.19 | 2.97 | -3.10 | 2.68 |
| **bH(2,6)-OBS(2)-235** | 15.04 | 8.29 | -0.43 | 3.43 | -2.96 | -3.20 | 3.27 | -3.14 | 2.76 | 2.91 |
| **bH(2,6)-OBS(2)-247** | 18.41 | 12.79 | -0.45 | 3.43 | -2.92 | 3.25 | -3.22 | 3.01 | 2.78 | -3.01 |
| **bH(4,5)-OBS(4)-147** | 14.10 | 10.40 | -0.36 | -3.46 | 3.41 | 3.29 | -3.24 | 3.11 | 3.17 | -2.93 |
| **bH(4,5)-OBS(4)-235** | 7.26 | 3.76 | -0.73 | 3.48 | -3.22 | -3.21 | 3.29 | -3.18 | 3.01 | 2.92 |
| **bH(4,5)-OBS(4)-247** | 10.87 | 7.87 | -0.32 | 3.42 | -3.26 | 3.33 | -3.23 | 2.90 | 2.97 | -3.04 |
| **bH(4,5)-OBS(4)-346** | 8.86 | 5.09 | 0.02 | 3.41 | 3.35 | -3.29 | -3.19 | 2.93 | -3.14 | 2.86 |
| **bH(2,6)-CBS(S2B)-235** | 15.60 | 8.90 | -0.36 | 3.43 | -3.16 | -3.20 | 3.36 | -2.69 | 2.07 | 3.05 |
| **bH(2,6)-CBS(S2Bt3)-147**[a] | 12.39 | 6.61 | -0.40 | -3.45 | 3.38 | 3.43 | -3.12 | 3.17 | 2.36 | -2.51 |
| **bH(2,6)-CBS(S2Bt3)-235**[a] | 9.91 | 4.74 | -0.35 | 3.43 | -3.16 | -3.21 | 3.35 | -2.68 | 2.03 | 3.04 |
| **bH(2,6t3)-CBS(S2Bt3)-235**[a] | 9.9 | 4.57 | -0.24 | 3.43 | -3.12 | -3.16 | 3.40 | -2.73 | 1.73 | 3.00 |
| **bH(4,5)-CBS(S3A)-247** | 15.29 | 11.48 | -0.46 | 3.43 | -3.26 | 3.31 | -3.16 | 2.97 | 2.94 | -3.03 |
| **bH(3,7)-CBS(S5A)-147** | 15.51 | 11.41 | -0.46 | -3.44 | 3.39 | 3.15 | -3.30 | 3.09 | 3.17 | -2.55 |
| **bH(3,7)-CBS(S5A)-235** | 7.76 | 3.22 | -0.63 | 3.45 | -3.22 | -3.18 | 3.28 | -3.11 | 3.00 | 2.86 |
| **bH(3,7)-CBS(S5A)-247** | 9.65 | 6.05 | -0.38 | 3.42 | -3.21 | 3.16 | -3.18 | 2.99 | 2.94 | -2.94 |
| **bH(3,7)-CBS(S5A)-346** | 8.28 | 4.03 | -0.33 | 3.43 | 3.34 | -3.08 | -3.20 | 2.98 | -3.16 | 2.75 |
| **tH(2)-CBS(S2B)-147** | 14.87 | 8.48 | -0.50 | -3.41 | 2.73 | 3.34 | -3.13 | 3.14 | 3.14 | -2.42 |
| **tH(2)-CBS(S2B)-235** | 15.69 | 9.47 | -0.60 | 3.42 | -2.61 | -3.28 | 3.30 | -3.07 | 2.62 | 2.80 |
| **tH(2)-CBS(S2B)-247** | 15.30 | 10.12 | -0.51 | 3.41 | -2.60 | 3.33 | -3.24 | 2.93 | 2.48 | -3.01 |
| **tH(2)-CBS(S2B)-346** | 13.74 | 7.82 | -0.19 | 3.46 | 2.86 | -3.21 | -3.15 | 2.98 | -2.72 | 2.86 |
| **tH(2)-CBS(S2Bt3)-147**[a] | 13.37 | 7.36 | -0.47 | -3.41 | 2.76 | 3.35 | -3.12 | 3.13 | 3.14 | -2.43 |
| **tH(2)-CBS(S2Bt3)-235**[a] | 12.37 | 6.47 | -0.56 | 3.43 | -2.58 | -3.28 | 3.31 | -3.10 | 2.48 | 2.85 |
| **tH(2)-CBS(S2Bt3)-346**[a] | 11.55 | 5.91 | -0.24 | 3.45 | 2.83 | -3.19 | -3.18 | 2.98 | -2.64 | 2.87 |
| **tH(3)-CBS(S2B)-147** | 18.06 | 13.06 | -0.65 | -3.41 | 3.28 | 2.70 | -3.17 | 3.12 | 3.12 | -2.00 |
| **tH(3)-CBS(S2B)-235** | 13.79 | 8.61 | -0.54 | 3.43 | -3.23 | -2.61 | 3.34 | -3.07 | 2.41 | 2.80 |
| **tH(3)-CBS(S2B)-247** | 14.36 | 9.66 | -0.43 | 3.43 | -3.22 | 2.94 | -3.17 | 3.01 | 2.87 | -2.49 |
| **tH(3)-CBS(S2B)-346** | 13.89 | 8.41 | -0.27 | 3.40 | 2.89 | -2.69 | -3.27 | 2.94 | -2.67 | 2.54 |
| **tH(3)-CBS(S2Bt3)-235**[a] | 11.83 | 7.07 | -0.53 | 3.43 | -3.25 | -2.60 | 3.34 | -3.07 | 2.39 | 2.82 |
| **tH(4)-CBS(S2B)-247** | 12.50 | 7.11 | -0.46 | 3.41 | -3.22 | 3.35 | -2.64 | 2.92 | 2.31 | -3.02 |
| **tH(4)-CBS(S2Bt3)-247**[a] | 9.00 | 4.58 | -0.41 | 3.41 | -3.19 | 3.37 | -2.62 | 2.90 | 2.19 | -3.01 |
| **tH(5)-CBS(S2B)-346** | 9.62 | 3.17 | -0.14 | 3.43 | 3.24 | -3.20 | -3.21 | 2.33 | -2.65 | 2.94 |
| **tH(5)-CBS(S2Bt3)-346**[a] | 7.55 | 1.35 | -0.19 | 3.43 | 3.24 | -3.20 | -3.21 | 2.33 | -2.58 | 2.94 |
| **tH(6)-CBS(S2B)-247** | 13.40 | 8.37 | -0.29 | 3.39 | -3.19 | 3.33 | -3.16 | 3.07 | 2.16 | -2.55 |
| **tH(6)-CBS(S2B)-247** | 11.73 | 6.67 | -0.30 | 3.39 | -3.19 | 3.34 | -3.16 | 3.05 | 2.12 | -2.52 |
| **tH(7)-CBS(S2B)-346** | 9.67 | 3.09 | -0.11 | 3.44 | 3.24 | -3.23 | -3.18 | 3.10 | -2.63 | 2.13 |
| **tH(7)-CBS(S2Bt3)-346**[a] | 8.40 | 1.88 | -0.14 | 3.43 | 3.25 | -3.24 | -3.18 | 3.10 | -2.57 | 2.14 |
| **tH(2,6)-np-CBS-247** | 13.29 | 8.85 | -0.30 | 3.40 | -2.68 | 3.24 | -3.20 | 3.01 | 2.26 | -2.92 |
| **noH-CBS(S2B,S3A)-247** | 16.31 | 16.41 | -0.31 | 3.35 | -3.23 | 3.13 | -3.14 | 2.91 | 2.97 | -2.65 |
| **noH-CBS(S2B,S5A)-247** | 2.72 | 3.38 | -0.41 | 3.36 | -3.21 | 2.95 | -3.28 | 3.10 | 2.91 | -2.51 |
| **noH-CBS(S2Bt3,S5A)-235**[a] | 7.00 | 6.96 | -0.33 | 3.11 | -3.23 | -3.19 | 3.26 | -2.78 | 3.02 | 2.97 |
| **noH-CBS(S2Bt3,S5A)-247**[a] | 1.06 | 1.34 | -0.37 | 3.36 | -3.20 | 2.95 | -3.27 | 3.08 | 2.87 | -2.52 |
| **noH-CBS(S2Bt3,S5A)-346**[a] | 0.00 | 0.00 | -0.24 | 3.36 | 2.98 | -3.21 | -3.29 | 3.07 | -2.55 | 2.86 |
| **bH(2,6)-OBS(6)-235** | 1.40 | 0.33 | -0.57 | 3.43 | -3.14 | -3.19 | 3.29 | -3.11 | 2.89 | 2.87 |
| **bH(2,6)-OBS(6)-247** | 0.30 | 1.42 | -0.46 | 3.43 | -3.15 | 3.32 | -3.16 | 2.98 | 2.86 | -3.02 |
| **bH(3,7)-OBS(7)-147** | 15.43 | 10.52 | -0.54 | -3.42 | 3.35 | 3.13 | -3.26 | 3.12 | 3.18 | -2.72 |

| | | | | | | | | | | |
|---|---|---|---|---|---|---|---|---|---|---|
| **bH(3,7)-OBS(7)-235** | 8.16 | 3.32 | -0.47 | 3.45 | -3.21 | -3.15 | 3.32 | -3.09 | 2.98 | 2.74 |
| **bH(3,7)-OBS(7)-247** | 11.63 | 8.77 | -0.56 | 3.40 | -3.22 | 3.26 | -3.19 | 3.04 | 2.99 | -3.06 |
| **bH(3,7)-OBS(7)-346** | 8.99 | 2.90 | -0.21 | 3.42 | 3.34 | -3.15 | -3.19 | 2.97 | -3.10 | 2.68 |
| **bH(2,6)-OBS(2)-235** | 13.92 | 8.36 | -0.43 | 3.43 | -2.96 | -3.20 | 3.27 | -3.14 | 2.76 | 2.91 |
| **bH(2,6)-OBS(2)-247** | 17.29 | 12.85 | -0.45 | 3.43 | -2.92 | 3.25 | -3.22 | 3.01 | 2.78 | -3.01 |
| **bH(4,5)-OBS(4)-147** | 12.98 | 10.46 | -0.36 | -3.46 | 3.41 | 3.29 | -3.24 | 3.11 | 3.17 | -2.93 |
| **bH(4,5)-OBS(4)-235** | 6.14 | 3.82 | -0.73 | 3.48 | -3.22 | -3.21 | 3.29 | -3.18 | 3.01 | 2.92 |
| **bH(4,5)-OBS(4)-247** | 9.75 | 7.93 | -0.32 | 3.42 | -3.26 | 3.33 | -3.23 | 2.90 | 2.97 | -3.04 |
| **bH(4,5)-OBS(4)-346** | 7.74 | 5.15 | 0.02 | 3.41 | 3.35 | -3.29 | -3.19 | 2.93 | -3.14 | 2.86 |
| **bH(2,6)-CBS(S2B)-235** | 14.48 | 8.96 | -0.36 | 3.43 | -3.16 | -3.20 | 3.36 | -2.69 | 2.07 | 3.05 |
| **bH(2,6)-CBS(S2Bt3)-147[a]** | 11.27 | 6.67 | -0.40 | -3.45 | 3.38 | 3.43 | -3.12 | 3.17 | 2.36 | -2.51 |
| **bH(2,6)-CBS(S2Bt3)-235[a]** | 8.78 | 4.80 | -0.35 | 3.43 | -3.16 | -3.21 | 3.35 | -2.68 | 2.03 | 3.04 |
| **bH(4,5)-CBS(S3A)-247** | 14.16 | 11.54 | -0.46 | 3.43 | -3.26 | 3.31 | -3.16 | 2.97 | 2.94 | -3.03 |
| **bH(3,7)-CBS(S5A)-147** | 14.39 | 11.47 | -0.46 | -3.44 | 3.39 | 3.15 | -3.30 | 3.09 | 3.17 | -2.55 |
| **bH(3,7)-CBS(S5A)-235** | 6.64 | 3.28 | -0.63 | 3.45 | -3.22 | -3.18 | 3.28 | -3.11 | 3.00 | 2.86 |
| **bH(3,7)-CBS(S5A)-247** | 8.53 | 6.11 | -0.38 | 3.42 | -3.21 | 3.16 | -3.18 | 2.99 | 2.94 | -2.94 |
| **bH(3,7)-CBS(S5A)-346** | 7.16 | 4.09 | -0.33 | 3.43 | 3.34 | -3.08 | -3.20 | 2.98 | -3.16 | 2.75 |
| **tH(2)-CBS(S2B)-147** | 13.75 | 8.54 | -0.50 | -3.41 | 2.73 | 3.34 | -3.13 | 3.14 | 3.14 | -2.42 |
| **tH(2)-CBS(S2B)-235** | 14.57 | 9.53 | -0.60 | 3.42 | -2.61 | -3.28 | 3.30 | -3.07 | 2.62 | 2.80 |
| **tH(2)-CBS(S2B)-247** | 14.18 | 10.18 | -0.51 | 3.41 | -2.60 | 3.33 | -3.24 | 2.93 | 2.48 | -3.01 |
| **tH(2)-CBS(S2B)-346** | 12.62 | 7.88 | -0.19 | 3.46 | 2.86 | -3.21 | -3.15 | 2.98 | -2.72 | 2.86 |
| **tH(2)-CBS(S2Bt3)-147[a]** | 12.25 | 7.42 | -0.47 | -3.41 | 2.76 | 3.35 | -3.12 | 3.13 | 3.14 | -2.43 |
| **tH(2)-CBS(S2Bt3)-235[a]** | 11.25 | 6.53 | -0.56 | 3.43 | -2.58 | -3.28 | 3.31 | -3.10 | 2.48 | 2.85 |
| **tH(2)-CBS(S2Bt3)-346[a]** | 10.43 | 5.97 | -0.24 | 3.45 | 2.83 | -3.19 | -3.18 | 2.98 | -2.64 | 2.87 |
| **tH(3)-CBS(S2B)-147** | 16.94 | 13.12 | -0.65 | -3.41 | 3.28 | 2.70 | -3.17 | 3.12 | 3.12 | -2.00 |
| **tH(3)-CBS(S2B)-235** | 12.67 | 8.67 | -0.54 | 3.43 | -3.23 | -2.61 | 3.34 | -3.07 | 2.41 | 2.80 |
| **tH(3)-CBS(S2B)-247** | 13.24 | 9.72 | -0.43 | 3.43 | -3.22 | 2.94 | -3.17 | 3.01 | 2.87 | -2.49 |
| **tH(3)-CBS(S2B)-346** | 12.77 | 8.47 | -0.27 | 3.40 | 2.89 | -2.69 | -3.27 | 2.94 | -2.67 | 2.54 |
| **tH(3)-CBS(S2Bt3)-235[a]** | 10.71 | 7.13 | -0.53 | 3.43 | -3.25 | -2.60 | 3.34 | -3.07 | 2.39 | 2.82 |
| **tH(4)-CBS(S2B)-247** | 11.38 | 7.17 | -0.46 | 3.41 | -3.22 | 3.35 | -2.64 | 2.92 | 2.31 | -3.02 |
| **tH(4)-CBS(S2Bt3)-247[a]** | 7.88 | 4.64 | -0.41 | 3.41 | -3.19 | 3.37 | -2.62 | 2.90 | 2.19 | -3.01 |
| **tH(5)-CBS(S2B)-346** | 8.49 | 3.24 | -0.14 | 3.43 | 3.24 | -3.20 | -3.21 | 2.33 | -2.65 | 2.94 |
| **tH(5)-CBS(S2Bt3)-346[a]** | 6.43 | 1.41 | -0.19 | 3.43 | 3.24 | -3.20 | -3.21 | 2.33 | -2.58 | 2.94 |
| **tH(6)-CBS(S2B)-247** | 12.28 | 8.43 | -0.29 | 3.39 | -3.19 | 3.33 | -3.16 | 3.07 | 2.16 | -2.55 |
| **tH(6)-CBS(S2Bt3)-247[a]** | 10.61 | 6.73 | -0.30 | 3.39 | -3.19 | 3.34 | -3.16 | 3.05 | 2.12 | -2.52 |
| **tH(7)-CBS(S2B)-346** | 8.55 | 3.15 | -0.11 | 3.44 | 3.24 | -3.23 | -3.18 | 3.10 | -2.63 | 2.13 |
| **tH(7)-CBS(S2Bt3)-346[a]** | 7.27 | 1.94 | -0.14 | 3.43 | 3.25 | -3.24 | -3.18 | 3.10 | -2.57 | 2.14 |
| **tH(2,6)-np-CBS-247** | 12.17 | 8.91 | -0.30 | 3.40 | -2.68 | 3.24 | -3.20 | 3.01 | 2.26 | -2.92 |

[a]t3 denotes a proton or a hydride that points to S3A, e.g. S2Bt3 is a protonated S2B where the proton points in the general direction of S3A (instead in direction of S5A)
[b]SH proton points toward homocitrate

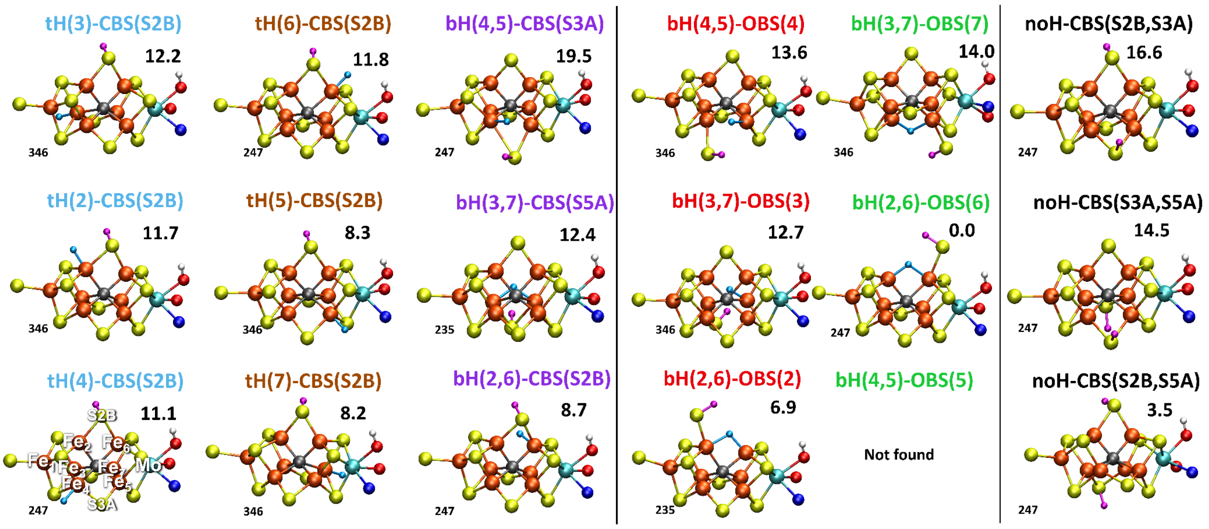

**Figure S2**. Calculated relative energies (polarized QM energy from the QM/MM calculation) of different $E_2$ models in the $\alpha$-$195^{His}$-$N_\delta$(H) protonation state. All models were calculated with 4 different broken-symmetry solutions with the lowest-energy one indicated. See Table S3 for additional information.

**Table S3.** Calculated relative energies in kcal/mol (TPSSh, both polarized QM and QM/MM energies are shown) and Mulliken spin populations on the metal ions for all 4 broken-symmetry solutions (235, 346, 247, 147—if available) of $E_2$ models in the $\alpha$-$195^{His}$-$N_\delta$ (H) protonation state in Fig. S2, using the QM/MM approach.

| Model | QM | QM/MM | Mo | $Fe_1$ | $Fe_2$ | $Fe_3$ | $Fe_4$ | $Fe_5$ | $Fe_6$ | $Fe_7$ |
|---|---|---|---|---|---|---|---|---|---|---|
| **noH-CBS(S2B,S3A)-247** | 16.60 | 16.21 | -0.29 | 3.34 | -3.24 | 3.15 | -3.13 | 2.90 | 2.95 | -2.66 |
| **noH-CBS(S2B,S5A)-147** | 9.75 | 7.24 | -0.55 | -3.42 | 3.13 | 3.17 | -3.28 | 3.14 | 3.13 | -2.25 |
| **noH-CBS(S2B,S5A)-235** | 9.98 | 8.49 | -0.32 | 3.11 | -3.24 | -3.18 | 3.26 | -2.79 | 3.04 | 2.96 |
| **noH-CBS(S2B,S5A)-247** | 3.48 | 1.99 | -0.38 | 3.35 | -3.22 | 2.96 | -3.27 | 3.08 | 2.90 | -2.52 |
| **noH-CBS(S2B,S5A)-346** | 3.52 | 1.94 | -0.24 | 3.36 | 3.01 | -3.19 | -3.30 | 3.07 | -2.58 | 2.83 |
| **noH-CBS(S3A,S5A)-247** | 14.51 | 17.62 | -0.31 | 3.36 | -3.26 | 3.00 | -3.16 | 2.91 | 2.94 | -2.58 |
| **tH(4)-OBS(5)-235** | 21.71 | 18.28 | -0.59 | 3.45 | -3.20 | -3.18 | 3.25 | -3.07 | 2.96 | 2.88 |
| **tH(4)-OBS(5)-247** | 19.28 | 16.36 | -0.41 | 3.43 | -3.26 | 3.32 | -3.20 | 2.99 | 2.85 | -3.04 |
| **bH(2,6)-OBS(6)-235** | 0.23 | -0.83 | -0.53 | 3.44 | -3.16 | -3.18 | 3.30 | -3.09 | 2.85 | 2.85 |
| **bH(2,6)-OBS(6)-247** | 0.00 | 0.00 | -0.48 | 3.43 | -3.15 | 3.32 | -3.15 | 2.97 | 2.81 | -2.99 |
| **bH(2,6)-OBS(6)-346** | 2.02 | 0.45 | -0.30 | 3.41 | 3.32 | -3.20 | -3.19 | 3.03 | -3.22 | 2.89 |
| **bH(3,7)-OBS(7)-147** | 21.56 | 15.55 | -0.48 | -3.42 | 3.35 | 3.14 | -3.24 | 3.11 | 3.11 | -2.74 |
| **bH(3,7)-OBS(7)-235** | 14.16 | 7.83 | -0.42 | 3.44 | -3.18 | -3.15 | 3.32 | -3.11 | 2.91 | 2.74 |
| **bH(3,7)-OBS(7)-247** | 17.02 | 12.81 | -0.54 | 3.40 | -3.20 | 3.27 | -3.19 | 3.05 | 2.91 | -3.05 |
| **bH(3,7)-OBS(7)-346** | 13.95 | 6.85 | -0.24 | 3.41 | 3.34 | -3.15 | -3.19 | 2.98 | -3.04 | 2.68 |
| **bH(2,6)-OBS(2)-147** | 13.02 | 8.28 | -0.54 | -3.42 | 2.94 | 3.34 | -3.19 | 3.12 | 3.19 | -2.42 |
| **bH(2,6)-OBS(2)-235** | 6.86 | 3.65 | -0.49 | 3.42 | -2.94 | -3.22 | 3.27 | -3.13 | 2.82 | 2.89 |
| **bH(2,6)-OBS(2)-247** | 9.95 | 8.06 | -0.50 | 3.43 | -2.93 | 3.26 | -3.22 | 3.01 | 2.84 | -3.00 |
| **bH(2,6)-OBS(2)-346** | 11.21 | 7.80 | -0.21 | 3.45 | 3.26 | -3.22 | -3.16 | 2.97 | -3.09 | 2.80 |
| **bH(3,7)-OBS(3)-147** | 21.13 | 17.72 | -0.57 | -3.44 | 3.39 | 3.29 | -3.29 | 3.09 | 3.11 | -2.65 |
| **bH(3,7)-OBS(3)-247** | 13.73 | 10.98 | -0.51 | 3.46 | -3.21 | 3.21 | -3.16 | 2.99 | 2.85 | -2.94 |
| **bH(3,7)-OBS(3)-346** | 12.71 | 8.65 | -0.23 | 3.41 | 3.35 | -3.11 | -3.20 | 3.00 | -3.08 | 2.78 |
| **bH(4,5)-OBS(4)-147** | 21.97 | 16.01 | -0.49 | -3.46 | 3.42 | 3.28 | -3.17 | 3.00 | 3.19 | -2.84 |
| **bH(4,5)-OBS(4)-247** | 15.25 | 10.47 | -0.47 | 3.45 | -3.22 | 3.31 | -3.17 | 2.87 | 2.97 | -3.02 |
| **bH(4,5)-OBS(4)-346** | 13.58 | 8.93 | -0.15 | 3.42 | 3.36 | -3.22 | -3.11 | 2.82 | -3.10 | 2.84 |
| **bH(2,6)-CBS(S2B)-147** | 13.17 | 7.96 | -0.38 | -3.44 | 3.41 | 3.42 | -3.12 | 3.17 | 2.35 | -2.51 |
| **bH(2,6)-CBS(S2B)-235** | 16.28 | 11.28 | -0.45 | 3.44 | -2.99 | -3.10 | 3.35 | -3.00 | 2.09 | 2.99 |
| **bH(2,6)-CBS(S2B)-247** | 8.67 | 4.16 | -0.26 | 3.41 | -3.17 | 3.43 | -3.13 | 3.07 | 1.75 | -2.58 |
| **bH(2,6)-CBS(S2B)-346** | 15.42 | 10.66 | -0.40 | 3.38 | 2.91 | -3.33 | -3.28 | 2.95 | -2.04 | 2.78 |
| **bH(2,6)-CBS(S2Bt3)-235[a]** | 14.87 | 11.92 | -0.53 | 3.46 | -3.07 | -3.20 | 3.27 | -3.09 | 2.65 | 2.94 |
| **bH(2,6)-CBS(S2Bt3)-346[a]** | 15.36 | 12.25 | -0.26 | 3.40 | 3.09 | -3.22 | -3.21 | 2.98 | -2.79 | 2.85 |
| **tH(5)-OBS(4)-235** | 12.79 | 8.88 | -0.69 | 3.48 | -3.20 | -3.20 | 3.29 | -3.18 | 2.94 | 2.92 |
| **bH(3,7)-CBS(S5A)-235** | 12.41 | 8.09 | -0.60 | 3.45 | -3.20 | -3.18 | 3.29 | -3.12 | 2.93 | 2.87 |
| **bH(3,7-t2)-CBS(S5A)-235[a]** | 20.05 | 17.29 | -0.56 | 3.44 | -3.18 | -2.93 | 3.32 | -3.16 | 2.88 | 2.67 |
| **tH(2)-CBS(S2B)-147** | 12.69 | 7.41 | -0.47 | -3.40 | 2.74 | 3.34 | -3.12 | 3.15 | 3.12 | -2.44 |
| **bH(4,5)-CBS(S3A)-247** | 19.45 | 16.27 | -0.42 | 3.43 | -3.26 | 3.32 | -3.18 | 2.98 | 2.85 | -3.03 |
| **tH(2)-CBS(S2B)-235** | 13.88 | 8.51 | -0.54 | 3.42 | -2.62 | -3.28 | 3.29 | -3.07 | 2.58 | 2.79 |
| **tH(2)-CBS(S2B)-247** | 13.03 | 8.70 | -0.47 | 3.40 | -2.59 | 3.35 | -3.23 | 2.94 | 2.39 | -3.01 |
| **tH(2)-CBS(S2B)-346** | 11.67 | 6.74 | -0.24 | 3.45 | 2.87 | -3.22 | -3.16 | 2.99 | -2.63 | 2.85 |
| **tH(2)-CBS(S2Bt3)-247[a]** | 14.73 | 10.87 | -0.47 | 3.40 | -2.62 | 3.31 | -3.24 | 2.90 | 2.50 | -2.98 |
| **tH(2)-CBS(S2Bt3)-346[a]** | 12.48 | 7.63 | -0.26 | 3.44 | 2.86 | -3.19 | -3.20 | 2.98 | -2.61 | 2.87 |
| **tH(3)-CBS(S2B)-147** | 16.14 | 12.80 | -0.55 | -3.40 | 3.39 | 2.66 | -3.14 | 3.10 | 3.11 | -2.18 |
| **tH(3)-CBS(S2B)-235** | 12.37 | 8.12 | -0.50 | 3.43 | -3.24 | -2.58 | 3.34 | -3.07 | 2.35 | 2.79 |
| **tH(3)-CBS(S2B)-247** | 12.75 | 8.87 | -0.41 | 3.43 | -3.22 | 2.96 | -3.16 | 3.00 | 2.86 | -2.51 |
| **tH(3)-CBS(S2B)-346** | 12.21 | 7.92 | -0.30 | 3.39 | 2.89 | -2.68 | -3.27 | 2.94 | -2.68 | 2.57 |
| **tH(3)-CBS(S2Bt3)-346[a]** | 13.12 | 9.28 | -0.32 | 3.39 | 2.93 | -2.66 | -3.27 | 2.96 | -2.81 | 2.64 |
| **tH(4)-CBS(S2B)-147** | 17.81 | 12.33 | -0.55 | -3.42 | 3.01 | 3.21 | -2.80 | 3.16 | 3.02 | -2.79 |
| **tH(4)-CBS(S2B)-235** | 11.95 | 6.76 | -0.38 | 3.43 | -3.15 | -3.18 | 2.97 | -2.70 | 2.91 | 2.91 |
| **tH(4)-CBS(S2B)-247** | 11.09 | 6.35 | -0.43 | 3.41 | -3.23 | 3.36 | -2.63 | 2.91 | 2.27 | -3.01 |

| | | | | | | | | | | |
|---|---|---|---|---|---|---|---|---|---|---|
| **tH(4)-CBS(S2B)-346** | 12.45 | 7.48 | -0.31 | 3.41 | 2.91 | -3.31 | -2.68 | 2.93 | -2.99 | 2.80 |
| **tH(4)-CBS(S2Bt3)-247**[a] | 11.95 | 7.37 | -0.41 | 3.41 | -3.22 | 3.36 | -2.62 | 2.90 | 2.20 | -2.99 |
| **tH(5)-CBS(S2B)-147** | 16.17 | 10.09 | -0.40 | -3.41 | 3.39 | 3.34 | -3.07 | 2.51 | 3.11 | -2.52 |
| **tH(5)-CBS(S2B)-235** | 15.27 | 10.47 | -0.49 | 3.40 | -3.19 | -3.18 | 2.93 | -2.65 | 2.95 | 2.87 |
| **tH(5)-CBS(S2B)-247** | 11.68 | 6.51 | -0.31 | 3.38 | -3.20 | 3.32 | -3.18 | 2.42 | 2.95 | -2.58 |
| **tH(5)-CBS(S2B)-346** | 8.32 | 2.71 | -0.19 | 3.42 | 3.26 | -3.20 | -3.22 | 2.32 | -2.58 | 2.94 |
| **tH(5)-CBS(S2Bt3)-346**[a] | 8.92 | 3.25 | -0.18 | 3.42 | 3.27 | -3.20 | -3.22 | 2.32 | -2.57 | 2.93 |
| **tH(6)-CBS(S2B)-147** | 15.78 | 11.94 | -0.33 | -3.40 | 3.41 | 3.29 | -3.10 | 3.15 | 2.33 | -2.46 |
| **tH(6)-CBS(S2B)-235** | 12.11 | 7.61 | -0.27 | 3.40 | -3.18 | -3.17 | 3.31 | -2.72 | 2.24 | 2.95 |
| **tH(6)-CBS(S2B)-247** | 11.76 | 7.50 | -0.27 | 3.39 | -3.18 | 3.34 | -3.16 | 3.07 | 2.15 | -2.57 |
| **tH(6)-CBS(S2B)-346** | 15.00 | 10.50 | -0.57 | 3.38 | 2.82 | -3.39 | -3.38 | 2.95 | -1.59 | 2.76 |
| **tH(6)-CBS(S2Bt3)-247**[a] | 11.86 | 8.45 | -0.35 | 3.39 | -3.20 | 3.34 | -3.15 | 3.05 | 2.14 | -2.52 |
| **tH(7)-CBS(S2B)-147** | 16.49 | 9.59 | -0.66 | -3.39 | 2.76 | 3.11 | -3.22 | 3.07 | 3.02 | -1.77 |
| **tH(7)-CBS(S2B)-235** | 11.97 | 5.97 | -0.25 | 3.41 | -3.18 | -3.18 | 3.30 | -2.75 | 3.00 | 2.28 |
| **tH(7)-CBS(S2B)-247** | 19.85 | 14.07 | -0.48 | 3.34 | -3.38 | 2.95 | -3.32 | 2.96 | 2.79 | -1.84 |
| **tH(7)-CBS(S2B)-346** | 8.17 | 2.40 | -0.15 | 3.42 | 3.26 | -3.23 | -3.19 | 3.10 | -2.56 | 2.13 |
| **tH(7)-CBS(S2Bt3)-346**[a] | 9.37 | 3.74 | -0.14 | 3.42 | 3.27 | -3.23 | -3.19 | 3.10 | -2.57 | 2.12 |
| **tH(3)endo-CBS(S2B)-235**[b] | 20.43 | 15.90 | -0.54 | 3.45 | -3.21 | -2.70 | 3.32 | -3.16 | 2.34 | 2.82 |
| **tH(4)endo-CBS(S2B)-235**[b] | 20.05 | 12.96 | -0.35 | 3.41 | -3.18 | -3.16 | 2.70 | -2.72 | 3.02 | 2.96 |
| **tH(5)endo-CBS(S2B)-235**[b] | 22.88 | 15.57 | -0.49 | 3.39 | -3.28 | -3.27 | 3.02 | -2.46 | 2.87 | 2.87 |
| **tH(7)endo-CBS(S2B)-235**[b] | 19.89 | 13.29 | -0.32 | 3.43 | -3.18 | -3.19 | 3.35 | -2.71 | 3.03 | 2.22 |
| **tH(2,6)-np-CBS-346** | 15.64 | 11.78 | -0.31 | 3.40 | -2.68 | 3.23 | -3.19 | 3.00 | 2.25 | -2.91 |

[a]t3 denotes a proton or a hydride that points to S3A, e.g. S2Bt3 is a protonated S2B where the proton points in the general direction of S3A (instead in direction of S5A)
[b]Endo refers to hydrides with an acute angle with respect to the central carbide while not bridging

**Table S4.** Calculated relative energies in kcal/mol (TPSSh level) and Mayer bond orders (MBO) of the hydrides in the hydride $E_2$ models from Fig. 3.

| Model | QM | MBO | Fe-H (Å) | MBO | Fe-H (Å) | Fe-Fe (Å) | Hirshfeld charge on H |
|---|---|---|---|---|---|---|---|
| **bH(4,5)-OBS(5)-247** | 0.00 | ( 4-Fe, 54-H ) : 0.6112 | 1.65815 | ( 5-Fe, 54-H ) : 0.4098 | 1.76747 | 2.57798 | -0.113134 |
| **bH(2,6)-OBS(6)-235** | 1.82 | ( 2-Fe, 55-H ) : 0.6071 | 1.65509 | ( 6-Fe, 55-H ) : 0.4289 | 1.77685 | 2.6946 | -0.128346 |
| **bH(3,7)-OBS(7)-235** | 0.64 | ( 3-Fe, 55-H ) : 0.6146 | 1.653 | ( 7-Fe, 55-H ) : 0.4097 | 1.77164 | 2.70705 | -0.12508 |
| **bH(2,6)-OBS(2)-247** | 3.74 | ( 2-Fe, 55-H ) : 0.3070 | 1.82473 | ( 6-Fe, 55-H ) : 0.7355 | 1.62903 | 2.67204 | -0.119744 |
| **bH(3,7)-OBS(3)-235** | 1.96 | ( 3-Fe, 55-H ) : 0.3106 | 1.82427 | ( 7-Fe, 55-H ) : 0.7271 | 1.62969 | 2.67328 | -0.126498 |
| **bH(4,5)-OBS(4)-346** | 5.44 | ( 4-Fe, 54-H ) : 0.3148 | 1.80545 | ( 5-Fe, 54-H ) : 0.7320 | 1.63264 | 2.65368 | -0.13025 |
| **bH(2,6)-CBS(S2B)-247** | 8.22 | ( 2-Fe, 55-H ) : 0.4760 | 1.74151 | ( 6-Fe, 55-H ) : 0.5196 | 1.74101 | 2.55666 | -0.119744 |
| **bH(3,7)-CBS(S5A)-247** | 3.19 | ( 7-Fe, 55-H ) : 0.8052 | 1.50855 | | | 2.52743 | -0.073414 |
| **bH(4,5)-CBS(S3A)-346** | 8.62 | ( 4-Fe, 54-H ) : 0.4738 | 1.73556 | ( 5-Fe, 54-H ) : 0.5230 | 1.73109 | 2.54091 | -0.119548 |
| **tH(2)-CBS(S2B)-346** | 10.81 | ( 2-Fe, 55-H ) : 0.7095 | 1.54495 | | | 2.70404 | -0.111611 |
| **tH(3)-CBS(S2B)-247** | 11.01 | ( 3-Fe, 55-H ) : 0.6727 | 1.5422 | | | 2.66257 | -0.100082 |
| **tH(4)-CBS(S2B)-235** | 11.38 | ( 4-Fe, 55-H ) : 0.6836 | 1.53844 | | | 2.63125 | -0.100148 |
| **tH(5)-CBS(S2B)-346** | 7.00 | ( 5-Fe, 55-H ) : 0.7181 | 1.51212 | | | 2.68099 | -0.112229 |
| **tH(6)-CBS(S2B)-247** | 7.65 | ( 6-Fe, 55-H ) : 0.7468 | 1.52313 | | | 2.76812 | -0.128192 |
| **tH(7)-CBS(S2B)-346** | 7.61 | ( 7-Fe, 55-H ) : 0.7515 | 1.51048 | | | 2.75019 | -0.109952 |

**Table S5.** Calculated relative energies in kcal/mol (TPSSh , polarized QM energy) and Mayer bond orders (MBO) of the hydrides in the hydride $E_2$ models in the $\alpha$-195$^{His}$-$N_\varepsilon$(H) protonation state in Fig. S1.

| Model | QM | MBO | Fe-H (Å) | MBO | Fe-H (Å) | Fe-Fe (Å) | Hirshfeld charge on H |
|---|---|---|---|---|---|---|---|
| **bH(2,6)-OBS(6)-247** | 0.00 | ( 95-Fe,132-H ) : 0.5438 | 1.63837 | ( 99-Fe,132-H ) : 0.3548 | 1.75816 | 2.65912 | -0.11504 |
| **bH(3,7)-OBS(7)-235** | 9.28 | ( 96-Fe,125-H ) : 0.6042 | 1.64379 | (100-Fe,125-H ) : 0.3819 | 1.76045 | 2.62454 | |
| **bH(2,6)-OBS(2)-235** | 15.04 | ( 95-Fe,132-H ) : 0.2870 | 1.78113 | ( 99-Fe,132-H ) : 0.6468 | 1.59699 | 2.59276 | -0.10548 |
| **tH(5)-OBS(4)-235** | 6.96 | ( 98-Fe,125-H ) : 0.8117 | 1.60108 | | | 2.73235 | |
| **bH(2,6)-CBS(S2B)-235** | 9.90 | ( 99-Fe,133-H ) : 0.7350 | 1.48827 | | | 2.48538 | |
| **bH(3,7)-CBS(S5A)-235** | 7.76 | ( 96-Fe,125-H ) : 0.4051 | 1.74864 | (100-Fe,125-H ) : 0.4518 | 1.71719 | 2.55478 | |
| **bH(4,5)-CBS(S3A)-247** | 15.28 | ( 97-Fe,125-H ) : 0.6724 | 1.6503 | ( 98-Fe,125-H ) : 0.2481 | 2.05858 | 2.60539 | |
| **tH(2)-CBS(S2B)-346** | 11.55 | ( 95-Fe,133-H ) : 0.7335 | 1.53741 | | | 2.64723 | |
| **tH(3)-CBS(S2B)-235** | 11.83 | ( 96-Fe,133-H ) : 0.7309 | 1.52078 | | | 2.63737 | |
| **tH(4)-CBS(S2B)-247** | 9.00 | ( 97-Fe,133-H ) : 0.7009 | 1.54982 | | | 2.72231 | |
| **tH(5)-CBS(S2B)-346** | 7.55 | ( 98-Fe,133-H ) : 0.7289 | 1.51765 | | | 2.70805 | |
| **tH(6)-CBS(S2B)-247** | 11.73 | ( 99-Fe,133-H ) : 0.8064 | 1.50492 | | | 2.68183 | |
| **tH(7)-CBS(S2B)-346** | 8.39 | (100-Fe,133-H ) : 0.8061 | 1.5156 | | | 2.64989 | |

**Table S6.** Calculated relative energies (TPSSh, polarized QM energy) and Mayer bond orders (MBO) of the hydrides in the hydride $E_2$ models in the α-195$^{His}$-$N_\delta$(H) protonation state in Fig. S2.

| Model | QM | MBO | Fe-H (Å) | MBO | Fe-H (Å) | Fe-Fe (Å) | Hirshfeld charge on H |
|---|---|---|---|---|---|---|---|
| **bH(2,6)-OBS(6)-247** | 0.00 | B( 95-Fe,132-H ) :0.5473 | 1.63471 | B( 99-Fe,132-H ): 0.3915 | 1.72551 | 2.655 | -0.104133 |
| **bH(3,7)-OBS(7)-346** | 13.95 | ( 96-Fe,125-H ) : 0.6095 | 1.64502 | (100-Fe,125-H ) : 0.4275 | 1.75147 | 2.62204 | |
| **bH(2,6)-OBS(2)-235** | 6.86 | ( 95-Fe,132-H ) : 0.3624 | 1.73446 | ( 99-Fe,132-H ) : 0.6646 | 1.63661 | 2.61733 | |
| **bH(3,7)-OBS(3)-346** | 12.71 | ( 96-Fe,125-H ) : 0.2694 | 1.86167 | (100-Fe,125-H ) : 0.5839 | 1.60592 | 2.54829 | |
| **bH(4,5)-OBS(4)-346** | 13.58 | ( 97-Fe,125-H ) : 0.2872 | 1.90039 | ( 98-Fe,125-H ) : 0.7133 | 1.6213 | 2.58988 | |
| **bH(2,6)-CBS(S2B)-247** | 8.67 | ( 95-Fe,133-H ) : 0.1147 | 2.13093 | ( 99-Fe,133-H ) : 0.7326 | 1.50692 | 2.50553 | -0.049903 |
| **bH(3,7)-CBS(S5A)-235** | 12.41 | ( 96-Fe,125-H ) : 0.4055 | 1.75046 | (100-Fe,125-H ) : 0.4534 | 1.71871 | 2.55956 | |
| **bH(4,5)-CBS(S3A)-247** | 19.45 | ( 97-Fe,125-H ) : 0.6803 | 1.64927 | ( 98-Fe,125-H ) : 0.2250 | 2.09041 | 2.62 | |
| **tH(2)-CBS(S2B)-346** | 11.67 | ( 95-Fe,133-H ) : 0.7081 | 1.54029 | | | 2.64195 | |
| **tH(3)-CBS(S2B)-346** | 12.21 | ( 96-Fe,133-H ) : 0.7417 | 1.51271 | | | 2.58934 | |
| **tH(4)-CBS(S2B)-247** | 11.09 | ( 97-Fe,133-H ) : 0.7035 | 1.5506 | | | 2.72853 | |
| **tH(5)-CBS(S2B)-346** | 8.32 | ( 98-Fe,133-H ) : 0.7271 | 1.5173 | | | 2.71378 | |
| **tH(6)-CBS(S2B)-247** | 11.76 | ( 99-Fe,133-H ) : 0.7742 | 1.5049 | | | 2.65481 | |
| **tH(7)-CBS(S2B)-346** | 8.17 | (100-Fe,133-H ) : 0.8016 | 1.51886 | | | 2.65791 | |

**Table S7.** Calculated relative energies in kcal/mol (TPSSh, both polarized QM and QM/MM energies are shown) and Mulliken spin populations on the metal ions of $E_0$, $E_1$, $E_2$ models in the α-195$^{His}$-$N_\varepsilon$(H) protonation state, using the QM/MM approach. BS solution is also indicated.

| Model | QM | QM/MM | Mo | $Fe_1$ | $Fe_2$ | $Fe_3$ | $Fe_4$ | $Fe_5$ | $Fe_6$ | $Fe_7$ |
|---|---|---|---|---|---|---|---|---|---|---|
| **$E_0$-235** | - | - | -0.54 | 3.43 | -3.21 | -3.21 | 3.30 | -3.12 | 2.98 | 2.90 |
| **$E_1$-S2B(H)-235** | 1.2 | 1.3 | -0.36 | 3.41 | -3.14 | -3.21 | 3.37 | -2.77 | 2.99 | 3.00 |
| **$E_1$-S2B(H)-346** | 0.4 | 0 | -0.31 | 3.46 | 3.30 | -3.21 | -3.19 | 3.06 | -2.62 | 2.94 |
| **E2-noHyd-247** | 2.2 | 1.3 | -0.37 | 3.36 | -3.20 | 2.95 | -3.27 | 3.08 | 2.87 | -2.52 |
| **E2-noHyd-346** | 1.1 | -0.1 | -0.24 | 3.36 | 2.98 | -3.21 | -3.29 | 3.07 | -2.55 | 2.86 |
| **E2-hyd-247** | 0.0 | 0.0 | -0.48 | 3.42 | -3.15 | 3.31 | -3.16 | 2.98 | 2.90 | -3.02 |
| **E2-hyd-235** | 2.5 | 0.3 | -0.57 | 3.43 | -3.14 | -3.19 | 3.29 | -3.11 | 2.89 | 2.87 |

| | | | | | | | | | | |
|---|---|---|---|---|---|---|---|---|---|---|
| **E2-hydTS-235** | 22.1 | 17.1 | -0.46 | 3.40 | -2.98 | -3.28 | 3.21 | -3.11 | 2.99 | 2.83 |

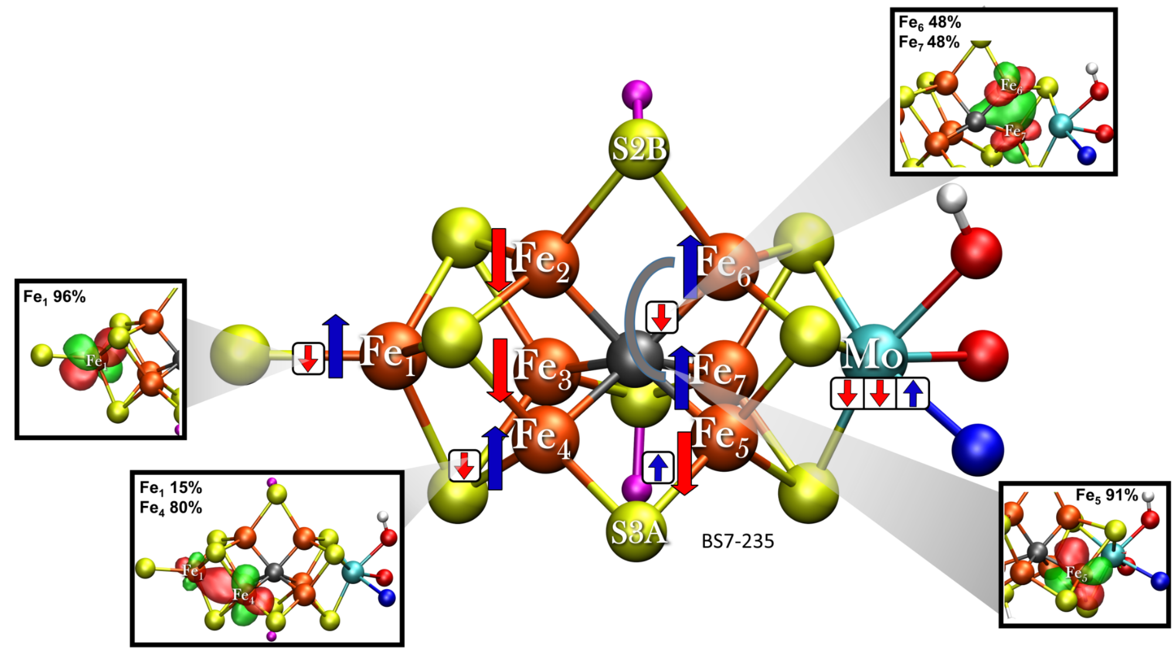


**Figure S3**. The electronic structure of the **$E_2$-nonhyd** model in the BS7-235 solution as interpreted via localized orbital analysis (Pipek-Mezey) and shown as Noodleman-style majority/minority spin vectors. Localized orbital isosurfaces (isovalue=0.05) of the minority-spin electrons are shown as insets. Large arrows indicate 5-electron s=5/2 majority-spin vectors, these 5 electrons are well localized. Small arrows indicate minority-spin single s=1/2 electrons that can be more delocalized.

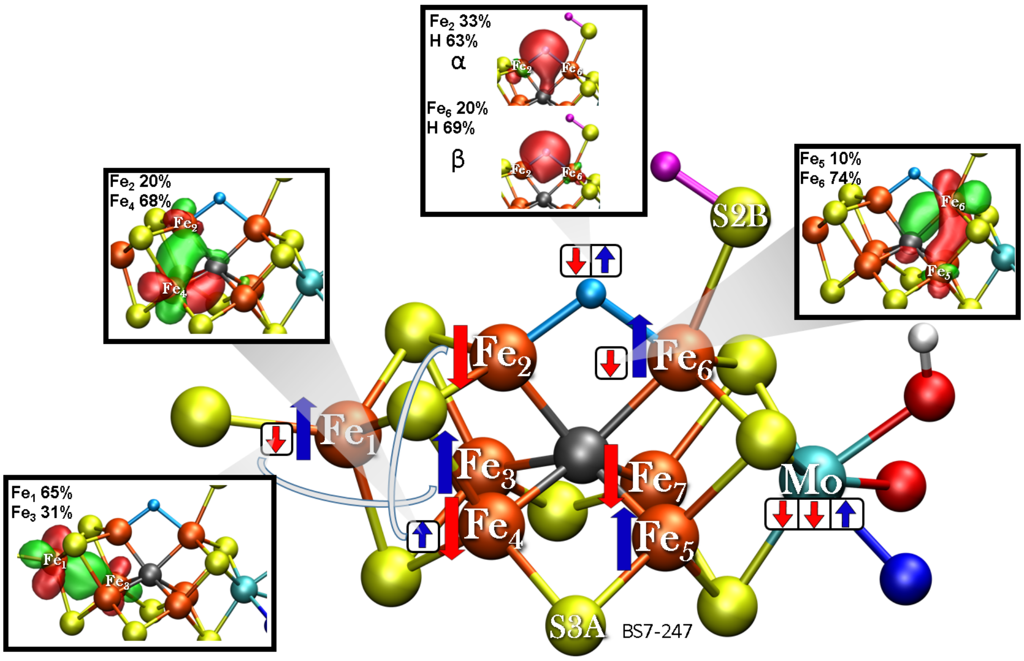


**Figure S4**. The electronic structure of the **$E_2$-hyd** model in the BS7-247 solution as interpreted via localized orbital analysis (Pipek-Mezey) and shown as Noodleman-

style majority/minority spin vectors. Localized orbital isosurfaces (isovalue=0.05) of the minority-spin electrons are shown as insets. Large arrows indicate 5-electron s=5/2 majority-spin vectors, these 5 electrons are well localized. Small arrows indicate minority-spin single s=1/2 electrons that can be more delocalized.

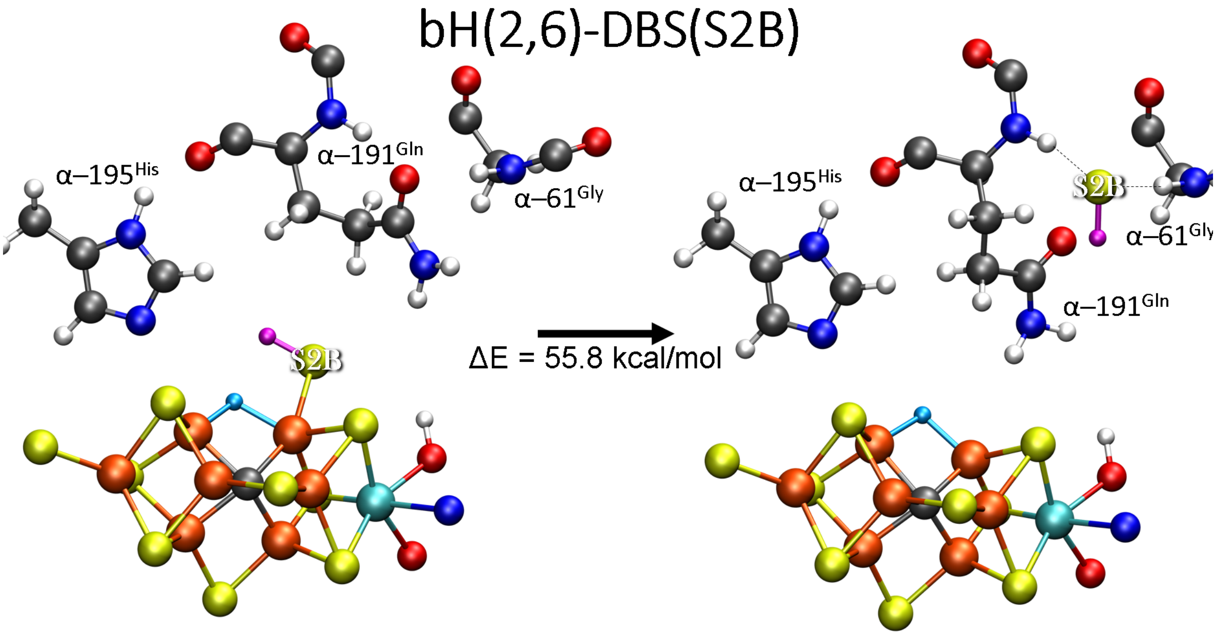


**Figure S5**. Structures of a **bH(2,6)-OBS(6)** model and a **bH(2,6)-DBS(S2B)** model (using BS7-235) where the S2B sulfide has dissociated in a protein pocket behind $\alpha$-191$^{Gln}$ and the relative energy between them (**bH(2,6)-OBS(6)** more stable). Calculations use an extended QM-region (143 atoms) that include residues: $\alpha$-61$^{Gln}$, $\alpha$-191$^{Gln}$, $\alpha$-195$^{His}$, $\alpha$-442$^{His}$, $\alpha$-275$^{Cys}$, $\alpha$-96$^{Arg}$, $\alpha$-339$^{Arg}$, $\alpha$-381$^{Phe}$ and $\alpha$-70$^{Val}$. $\alpha$-195$^{His}$ is in the $N_\delta$(H) protonation state.

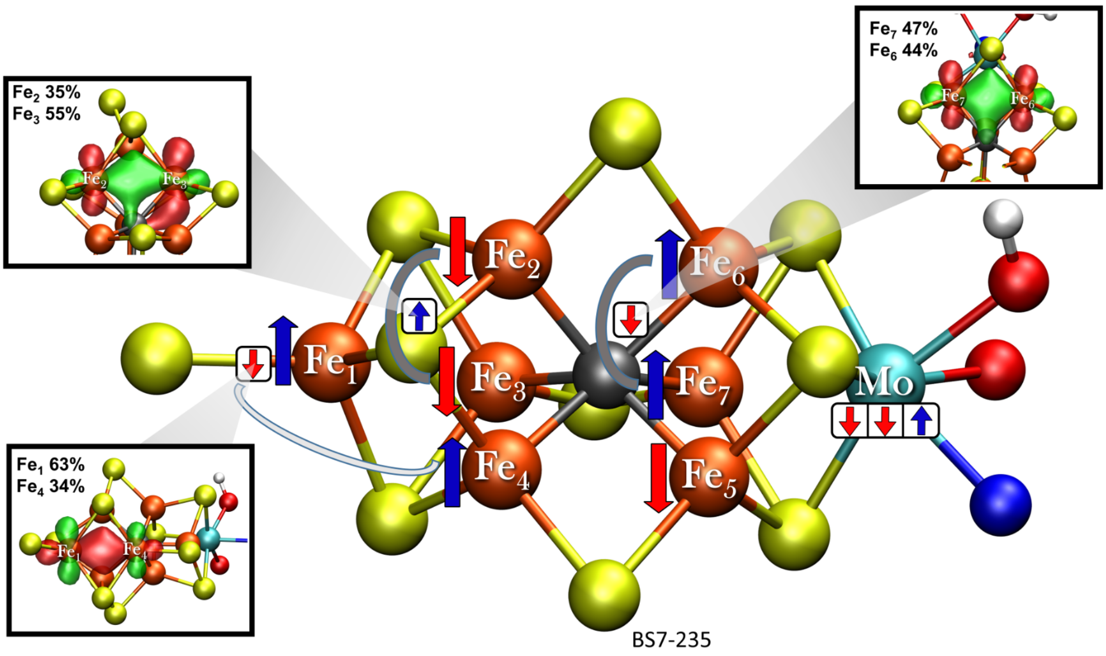


**Figure S6**. The electronic structure of the $\mathbf{E_0}$ model in the BS7-235 solution (QM/MM, $\alpha$-195$^{His}$-$N_\varepsilon$(H)) as interpreted via localized orbital analysis (Pipek-Mezey) and shown

as Noodleman-style majority/minority spin vectors. Localized orbital isosurfaces (0.05 isovalue) of the minority-spin electrons are shown as insets. Large arrows indicate 5-electron s=5/2 majority-spin vectors, these 5 electrons are well localized. Small arrows indicate minority-spin single s=1/2 electrons that can be more delocalized.

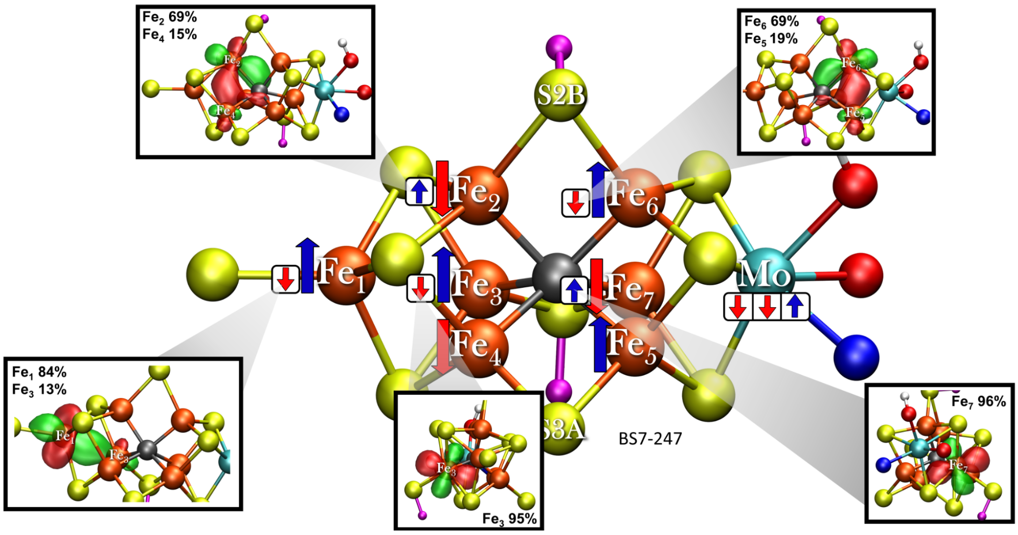


**Figure S7**. The electronic structure of the **$E_2$-nonhyd** model in the BS7-247 solution as interpreted via localized orbital analysis (Pipek-Mezey) and shown as Noodleman-style majority/minority spin vectors. Localized orbital isosurfaces (0.05 isovalue) of the minority-spin electrons are shown as insets. Large arrows indicate 5-electron s=5/2 majority-spin vectors, these 5 electrons are well localized. Small arrows indicate minority-spin single s=1/2 electrons that can be more delocalized.

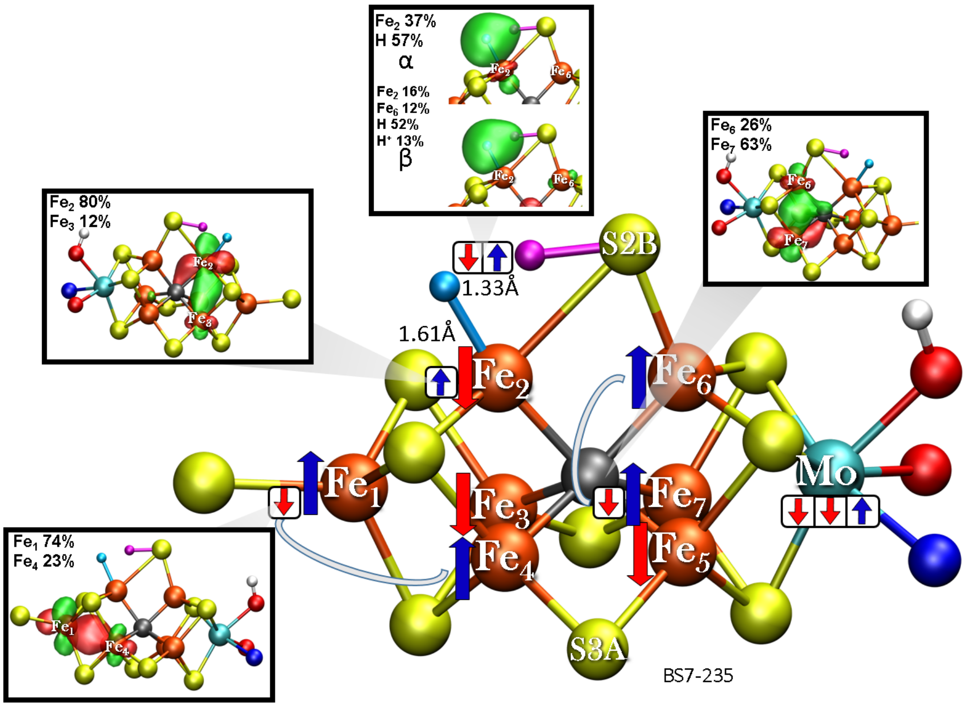


**Figure S8.** The electronic structure of the saddlepoint structure (**$E_2$-hyd-TS**) for $H_2$ formation. The model shown has $\alpha$-195$^{His}$ in the $N_\delta$ protonation state.

### Effect of QM-region expansion near sulfide S3A

Figure S9 shows the effect of increasing the QM-region from 134 atoms to 214 atoms by including the peptide backbone surrounding S3A (featuring weak NH...S hydrogen bonds) on the relative energies of the most stable $E_2$ isomers.

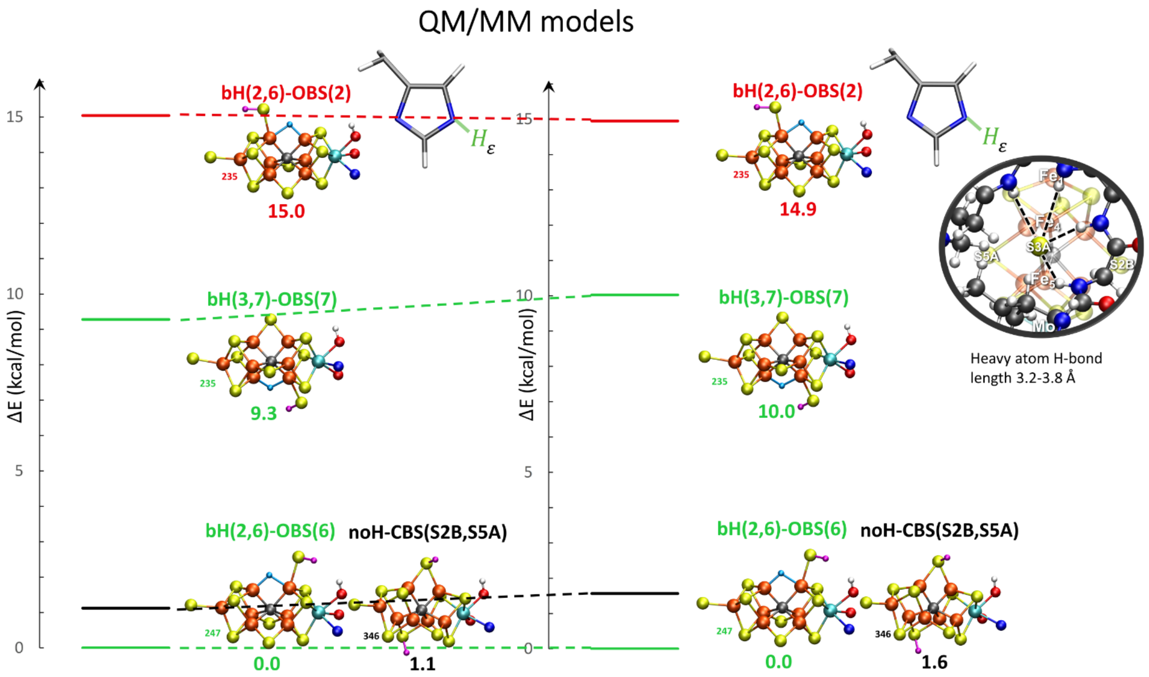


**Figure S9.** The change in reaction energies of the QM/MM models of selected $E_2$ models with $\alpha$-195$^{His}$-$N_\varepsilon$(H) state when increasing the QM-region from 134 atoms to 214 atoms, by adding the residues $\alpha$-355$^{Ile}$, $\alpha$-356$^{Gly}$, $\alpha$-357$^{Gly}$, $\alpha$-358$^{Leu}$ and $\alpha$-359$^{Arg}$ surrounding sulfide S3A.

### The stability of a protonated carbide model

Two different protonated carbide models (see Figure S10) were tested and compared to the most stable hydride isomer (**bH(2,6)-OBS(6)** in the BS7-247 state); these were **pC(1)-CBS(S2B)** in the BS7-346 state and **pC(2)-np-CBS** in the BS7-346 . Their relative energies reveal that carbide protonation is quite uphill in energy compared to forming a hydride formation.

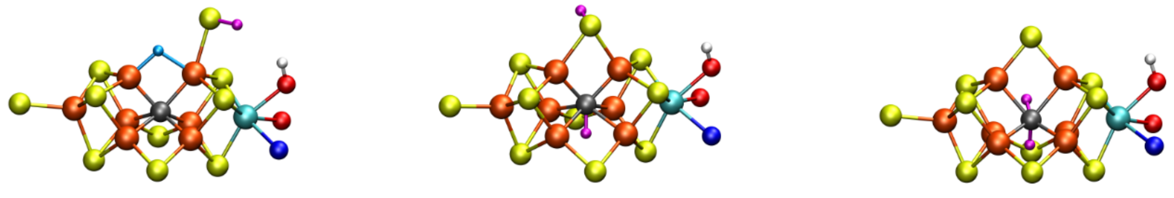

| | bH(2,6)-OBS(6)-247 | pC(1)-CBS(S2B)-346 | pC(2)-np-CBS-346 |
|---|---|---|---|
| QM(kcal/mol) | 0.0 | 20.1 | 32.2 |
| QM/MM(kcal/mol) | 0.0 | 15.5 | 28.2 |

Figure S10. Geometries of protonated carbide models (middle and right) compared to the most stable hydride model (left) as well as polarized QM energies and full QM/MM energies (kcal/mol).

## The orientations of hydrogens in $E_2$ isomers

Previous investigations on reduced states of FeMoco, by us and Ryde and coworkers, have found that the direction of protonated sulfides of the FeMoco cluster is affected by a fair amount of the protein environment. The scope of the present study was partially based on previous studies[1,2,3] that investigated proton orientation aspects in various $E_n$ states.

Ryde *et al.* found that for $E_1$ (using TPSS), S2B protonation is preferred to be oriented towards S3A, the other direction being uphill by ~1.7 kcal/mol, while for S5A the preferred direction is towards S3A, the other direction being uphill by ~5.0 kcal/mol.[3] Protonation of S3A was high in energy, ~13 kcal/mol uphill, with little difference in direction of the proton, towards S5A being ~0.5 kcal/mol uphill.

Our own result for $E_1$ (with TPSSh) agree with this result as we find an energy difference of ~1.4 kcal/mol for different orientations of S2B protonation, however, this is affected by $\alpha$-195$^{His}$ protonation scheme.

For $E_2$ models, with the $\alpha$-195$^{His}$-$N_\varepsilon$(H) scheme, the S2B protonation is preferred to orient towards S3A (5.7 kcal/mol for **$E_2$-bH(2,6)-CBS(S2B)**), similar to that found by Ryde and coworkers, but with the $\alpha$-195$^{His}$-$N_\delta$(H) scheme the S5A direction becomes favoured instead, with the S3A direction 1.3 kcal/mol less favorable for **$E_2$-bH(2,6)-CBS(S2B)**. This has to do with favourable hydrogen bonds to $\alpha$-195$^{His}$, as shown in Figure S11. Similarly, the S5A protonation is also found to be preferred towards S3A, ~6.3 kcal/mol uphill for $E_4$ models. This is due to the hydrogen bonds that are formed with $\alpha$-359$^{Arg}$ and $\alpha$-96$^{Arg}$, though the hydrogen bond with $\alpha$-96$^{Arg}$ is more important in this context, see Figure S12. Protonating S3A is always ~10-20 kcal/mol uphill in energy due to the steric effects of the nearby protein backbone as shown in Figure S13.

As hydrides are also present in the $E_2$ models, we also tried different directions for hydrides, terminal hydrides that are trans to the interstitial carbide vs. endo hydrides (with an acute angle with respect to the central carbide while not bridging two Fe ions), bridging hydrides that point towards different sides of the dihedral plane formed by the central carbide, two binding Fe and $\mu^2$-S. Terminal hydrides trans to the carbide were generally favoured, from 1.2-8.2 kcal/mol depending on what Fe forms a hydride and what sulfide gets protonated, though terminal hydrides are generally unfavored. With the bridging hydrides, as in the case of protonated belt-sulfides, the direction preferred was dependent on the $\alpha$-195$^{His}$ protonation scheme. For the $\alpha$-195$^{His}$-$N_\varepsilon$(H) scheme the different directions are approximately equal in energy. For the $\alpha$-195$^{His}$-$N_\delta$(H) protonation scheme the hydride is preferred to be oriented towards S5A by ~4.3 kcal/mol (**$E_2$-bH(2,6)-**

[1] Van Stappen, C.; Thorhallsson, A. T.; Decamps, L.; Bjornsson, R.; DeBeer, S. Resolving the Structure of the E 1 State of Mo Nitrogenase through Mo and Fe K-Edge EXAFS and QM/MM Calculations. *Chem. Sci.* **2019**, *10* (42), 9807–9821.

[2] Thorhallsson, A. T.; Benediktsson, B.; Bjornsson, R. A Model for Dinitrogen Binding in the E 4 State of Nitrogenase. *Chemical Science* **2019**, *10* (48), 11110–11124. https://doi.org/10.1039/C9SC03610E.

[3] Cao, L.; Caldararu, O.; Ryde, U. Protonation and Reduction of the FeMo Cluster in Nitrogenase Studied by Quantum Mechanics/Molecular Mechanics (QM/MM) Calculations. *J. Chem. Theory Comput.* **2018**, *14* (12), 6653–6678.

**CBS(S2B)**). Lastly, we investigated rotations of the open protonated belt sulfides and found a preference for orienting towards the $\alpha$-195$^{His}$ amino acid residue for $\alpha$-195$^{His}$-$N_{\varepsilon}$(H) scheme and towards the homocitrate ligand for the $\alpha$-195$^{His}$-$N_{\varepsilon}$(H) scheme in the case of the lowest hydride structure (**$E_2$-hyd**). As the dihedral angle of the hydrides for the **OBS** models is close to planar, there is no preference involved for the hydrides.

While we find these hydrogen orientations to be non-negligible and we tried to probe the most relevant orientation in each case, it was not possible to try every conceivable permutation of protons/hydride on the cofactor. In our experience this orientation dependence has a small effect on the electronic and molecular structure of the cofactor but rather reflects the H-bonding network surrounding the cofactor. Future work may involve a dynamics-based approach to further study this hydrogen orientation dependence.

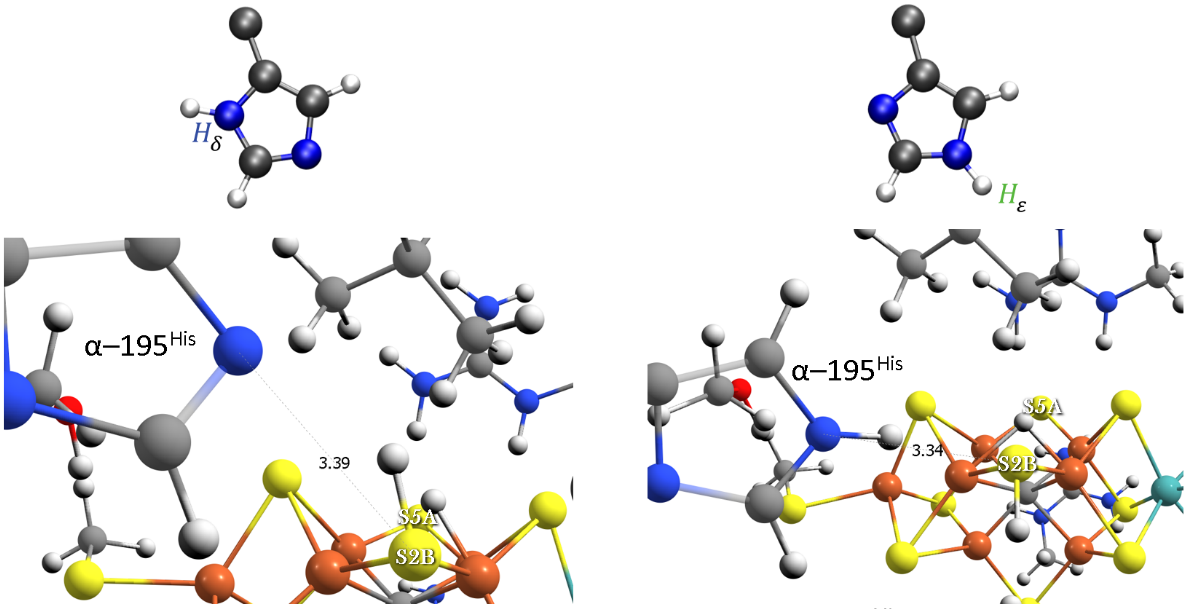


Figure S11. Orientation of the S2B proton depending on $\alpha$-195$^{His}$ protonation state. For the $\alpha$-195$^{His}$-$N_{\varepsilon}$(H) protonation state, orientation of the S2B proton in the S3A direction is favoured while for $\alpha$-195$^{His}$-$N_{\delta}$(H) the orientation towards S5A is lower in energy. N…S H-bond distances are shown in Å.

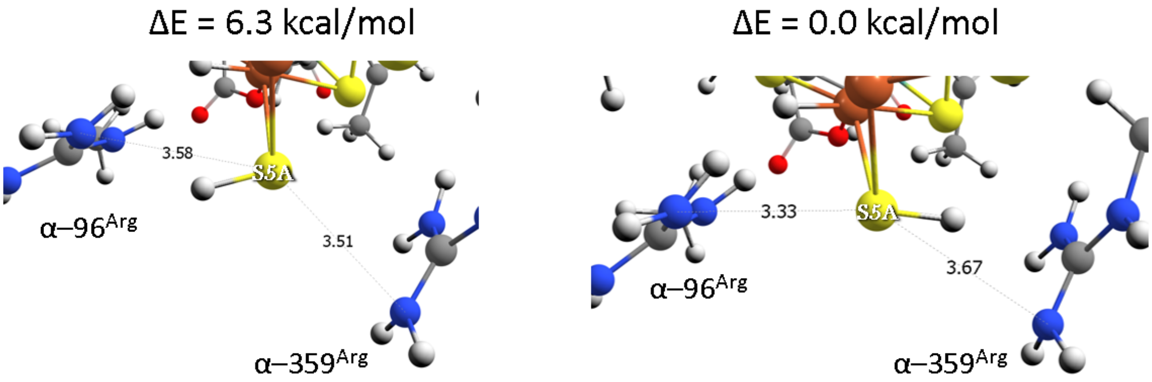


Figure S12. Orientation of the S5A proton. A strong H-bond is formed between S5A and $\alpha$-96$^{Arg}$ if the proton is oriented towards S3A. N…S H-bond distances are shown in Å.

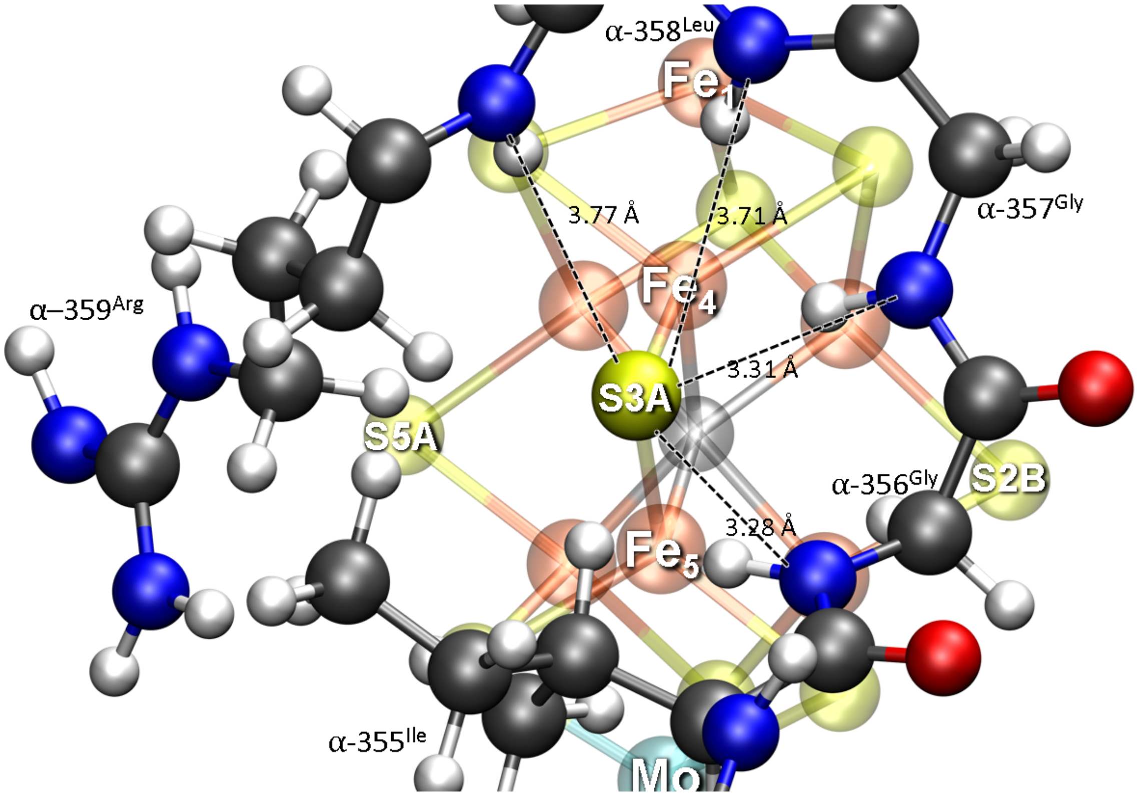


**Figure S13.** A closeup of the protein backbone surrounding S3A. N…S hydrogen bond distances are indicated.

## The effect of basis set and density functional choice on stability of models

In order to test the sensitivity of our computational protocol with respect to basis set size and functional choice we recalculated the energies (full geometry optimizations with the cluster-continuum model) with either a non-hybrid functional TPSS or the hybrid-functional TPSSh (results were calculated without ZORA ) and either a double-zeta basis set def2-SVP or a triple-zeta basis set def2-TZVP (should be reasonably close to the basis set limit). The results are seen in Table S8. They reveal that there is a basis set dependency present that can affect the relative energies by a few kcal/mol that in turns affects slightly the energetic ordering of isomers. A larger effect is seen by changing the functional from TPSSh to TPSS, which reveal that together with basis set effects, different computational protocols can lead to complete disagreement about what the most stable isomer for $E_2$ is. As found in our previous work (Thorhallsson et al. *Chem. Sci.* **2019**, *10*, 11110-11124), the TPSSh functional (using a triple-zeta basis set) leads to much better agreement with the high-resolution crystal structure than TPSS.

**Table S8.** Calculated relative energies (kcal/mol) various hydride and non-hydride $E_2$ models using the cluster-continuum models with different functionals (ZORA was not included in this comparison).

| | bH(3,7)-CBS(S5A)-247 | bH(3,7)-CBS(S5A)-346 | bH(4,5)-OBS(5)-247 | bH(4,5)-OBS(5)-346 | noH-CBS(S2B,S5A)-247 | noH-CBS(S2B,S5A)-346 | tH(5)-CBS(S2B)-247 | tH(5)-CBS(S2B)-346 |
|---|---|---|---|---|---|---|---|---|

| | | | | | | | | | |
|---|---|---|---|---|---|---|---|---|---|
| TPSSh/def2-SVP | 13.69 | 7.52 | 2.43 | 5.25 | 0.00 | 1.29 | 14.58 | 12.25 |
| TPSSh/def2-TZVP | 12.72 | 11.85 | 2.36 | 6.91 | 0.00 | 0.01 | 12.90 | 10.71 |
| TPSS/def2-SVP | 0.94 | 0.00 | 0.98 | 1.51 | 5.74 | 6.14 | 7.49 | 3.45 |
| TPSS/def2-TZVP | 0.01 | 0.00 | 1.70 | 2.72 | 2.10 | 2.82 | 6.45 | 2.90 |

## Localized orbital populations

Tables S9-S15 show populations of the Pipek-Mezey localized orbitals for the indicated BS-state models.

**Table S9.** Localized orbital populations of the **$E_0$** BS7-235 state.

| | $d^1$ | $d^2$ | $d^3$ | $d^4$ | $d^5$ | $d^6$ |
|---|---|---|---|---|---|---|
| Mo | MO 344β: Fe6 24% & Mo 72% | MO 345β: Fe7 31% & Mo 67% | MO 347α: Fe5 48% & Mo 50% | | | |
| $Fe_1$ | MO 173α: Fe1 99% | MO 174α: Fe1 95% | MO 175α: Fe1 99% | MO 176α: Fe1 99% | MO 177α: Fe1 96% | MO 342β: Fe4 34% & Fe1 63% |
| $Fe_2$ | MO 177β: Fe2 99% | MO 181β: Fe2 95% | MO 182β: Fe2 93% | MO 183β: Fe2 93% | MO 184β: Fe2 97% | MO 346α: Fe3 55% & Fe2 35% |
| $Fe_3$ | MO 186β: Fe3 99% | MO 190β: Fe3 97% | MO 191β: Fe3 95% | MO 192β: Fe3 93% | MO 193β: Fe3 94% | MO 346α: Fe3 55% & Fe2 35% |
| $Fe_4$ | MO 190α: Fe4 99% | MO 191α: Fe4 99% | MO 192α: Fe4 93% | MO 196α: Fe4 97% | MO 197α: Fe4 96% | |
| $Fe_5$ | MO 199β: Fe5 99% | MO 202β: Fe5 97% | MO 204β: Fe5 94% | MO 205β: Fe5 95% | MO 343β: Fe5 90% & Mo 7% | |
| $Fe_6$ | MO 203α: Fe6 99% | MO 207α: Fe6 95% | MO 208α: Fe6 93% | MO 209α: Fe6 96% | MO 348α: Fe6 86% & Mo 11% | MO 346β: Fe7 47% & Fe6 44% |
| $Fe_7$ | MO 212α: Fe7 99% | MO 213α: Fe7 97% | MO 216α: Fe7 95% | MO 217α: Fe7 93% | MO 349α: Fe7 85% & Mo 13% | MO 346β: Fe7 47% & Fe6 44% |

**Table S10.** Localized orbital populations of the **$E_1$-S2B(H)** BS7-346 state. $\alpha$-195$^{His}$-$N_\varepsilon$(H).

| | $d^1$ | $d^2$ | $d^3$ | $d^4$ | $d^5$ | $d^6$ |
|---|---|---|---|---|---|---|
| Mo | MO 343β: Fe5 34% & Mo 62% | MO 345β: Fe7 32% & Mo 66% | MO 346α: Fe6 28% & Mo 69% | | | |
| $Fe_1$ | MO 173α: Fe1 99% | MO 174α: Fe1 99% | MO 175α: Fe1 95% | MO 176α: Fe1 97% | MO 177α: Fe1 99% | MO 342β: Fe2 37% & Fe1 61% |
| $Fe_2$ | MO 182α: Fe2 99% | MO 183α: Fe2 99% | MO 184α: Fe2 95% | MO 188α: Fe2 96% | MO 189α: Fe2 95% | MO 342β: Fe2 37% & Fe1 61% |
| $Fe_3$ | MO 181β: Fe3 99% | MO 183β: Fe3 97% | MO 186β: Fe3 94% | MO 187β: Fe3 95% | MO 188β: Fe3 93% | |
| $Fe_4$ | MO 190β: Fe4 99% | MO 194β: Fe4 94% | MO 195β: Fe4 97% | MO 196β: Fe4 96% | MO 197β: Fe4 94% | MO 375α: 110C 18% & Fe4 68% |
| $Fe_5$ | MO 200α: Fe5 99% | MO 203α: Fe5 95% | MO 204α: Fe5 97% | MO 205α: Fe5 95% | MO 345α: Fe5 88% & Mo 9% | MO 346β: Fe7 43% & Fe5 47% |
| $Fe_6$ | MO 206β: Fe6 96% | MO 207β: Fe6 90% | MO 208β: Fe6 95% | MO 209β: Fe6 92% | MO 344β: Fe6 84% & Mo 12% | MO 210α: Fe6 96% |
| $Fe_7$ | MO 212α: Fe7 97% | MO 216α: Fe7 99% | MO 217α: Fe7 94% | MO 218α: Fe7 95% | MO 347α: Fe7 85% & Mo 12% | MO 346β: Fe7 43% & Fe5 47% |

**Table S11.** Localized orbital populations of the **$E_2$-nohyd** BS7-247 state. $\alpha$-195$^{His}$-$N_\varepsilon$(H).

| | $d^1$ | $d^2$ | $d^3$ | $d^4$ | $d^5$ | $d^6$ |
|---|---|---|---|---|---|---|
| Mo | MO 345β: Fe5 34% & Mo 64% | MO 346β: Fe6 30% & Mo 67% | MO 348α: Fe7 32% & Mo 67% | | | |
| $Fe_1$ | MO 174α: Fe1 99% | MO 175α: Fe1 96% | MO 176α: Fe1 98% | MO 179α: Fe1 99% | MO 181α: Fe1 94% | MO 344β: Fe3 13% & Fe1 84% |
| $Fe_2$ | MO 178β: Fe2 99% | MO 181β: Fe2 95% | MO 182β: Fe2 95% | MO 183β: Fe2 95% | MO 184β: Fe2 96% | MO 415α: Fe2 69% Fe4 15% 110C 12% |

| | | | | | | |
|---|---|---|---|---|---|---|
| $Fe_3$ | MO 190α: Fe3 96% | MO 191α: Fe3 94% | MO 192α: Fe3 91% | MO 193α: Fe3 99% | MO 194α: Fe3 94% | MO 189β: Fe3 95% |
| $Fe_4$ | MO 191β: Fe4 99% | MO 192β: Fe4 98% | MO 193β: Fe4 96% | MO 197β: Fe4 96% | MO 198β: Fe4 95% | |
| $Fe_5$ | MO 200α: Fe5 99% | MO 204α: Fe5 97% | MO 205α: Fe5 96% | MO 206α: Fe5 95% | MO 346α: Fe5 88% & Mo 9% | |
| $Fe_6$ | MO 210α: Fe6 98% | MO 212α: Fe6 94% | MO 213α: Fe6 95% | MO 214α: Fe6 95% | MO 347α: Fe6 85% & Mo 12% | MO 347β: Fe6 69% & Fe5 20% |
| $Fe_7$ | MO 211β: Fe7 92% | MO 212β: Fe7 93% | MO 213β: Fe7 96% | MO 214β: Fe7 95% | MO 348β: Fe7 83% & Mo 14% | MO 219α: Fe7 96% |

**Table S12.** Localized orbital populations of the **$E_2$-nohyd** BS7-235 state. α-195$^{His}$-$N_ε$(H).

| | $d^1$ | $d^2$ | $d^3$ | $d^4$ | $d^5$ | $d^6$ |
|---|---|---|---|---|---|---|
| Mo | MO 347α: Fe5 27% & Mo 70% | MO 344β: Fe6 30% & Mo 67% | MO 345β: Fe7 38% & Mo 62% | | | |
| $Fe_1$ | MO 173α: Fe1 100% | MO 174α: Fe1 98% | MO 178α: Fe1 98% | MO 179α: Fe1 92% | MO 180α: Fe1 91% | MO 176β: Fe1 96% |
| $Fe_2$ | MO 178β: Fe2 95% | MO 179β: Fe2 97% | MO 183β: Fe2 96% | MO 184β: Fe2 98% | MO 185β: Fe2 95% | |
| $Fe_3$ | MO 187β: Fe3 98% | MO 191β: Fe3 94% | MO 192β: Fe3 96% | MO 193β: Fe3 96% | MO 194β: Fe3 95% | |
| $Fe_4$ | MO 190α: Fe4 99% | MO 192α: Fe4 99% | MO 195α: Fe4 96% | MO 196α: Fe4 96% | MO 197α: Fe4 94% | MO 342β: Fe4 80% & Fe1 15% |
| $Fe_5$ | MO 203β: Fe5 95% | MO 204β: Fe5 98% | MO 205β: Fe5 93% | MO 206β: Fe5 93% | MO 343β: Fe5 85% & Mo 11% | MO 202α: Fe5 91% |
| $Fe_6$ | MO 204α: Fe6 97% | MO 208α: Fe6 99% | MO 209α: Fe6 95% | MO 210α: Fe6 95% | MO 348α: Fe6 87% & Mo 11% | MO 346β: Fe7 48% & Fe6 48% |
| $Fe_7$ | MO 212α: Fe7 99% | MO 216α: Fe7 98% | MO 217α: Fe7 96% | MO 218α: Fe7 95% | MO 349α: Fe7 86% & Mo 12% | MO 346β: Fe7 48% & Fe6 48% |

**Table S13.** Localized orbital populations of the **$E_2$-hyd** BS7-235 state. α-195$^{His}$-$N_ε$(H).

| | $d^1$ | $d^2$ | $d^3$ | $d^4$ | $d^5$ | $d^6$ |
|---|---|---|---|---|---|---|
| Mo | MO 344α: Fe5 47% & Mo 50% | MO 342β: Fe6 21% & Mo 73% | MO 343β: Fe7 33% & Mo 65% | | | |
| $Fe_1$ | MO 173α: Fe1 99% | MO 174α: Fe1 97% | MO 175α: Fe1 95% | MO 176α: Fe1 99% | MO 177α: Fe1 99% | MO 340β: Fe4 30% & Fe1 68% |
| $Fe_2$ | MO 178β: Fe2 95% | MO 179β: Fe2 97% | MO 183β: Fe2 96% | MO 184β: Fe2 98% | MO 185β: Fe2 95% | MO 343α: Fe3 63% & Fe2 27% |
| $Fe_3$ | MO 186β: Fe3 99% | MO 190β: Fe3 94% | MO 191β: Fe3 93% | MO 192β: Fe3 95% | MO 193β: Fe3 96% | MO 343α: Fe3 63% & Fe2 27% |
| $Fe_4$ | MO 190α: Fe4 99% | MO 191α: Fe4 99% | MO 192α: Fe4 96% | MO 196α: Fe4 97% | MO 197α: Fe4 93% | |
| $Fe_5$ | MO 199β: Fe5 96% | MO 202β: Fe5 99% | MO 204β: Fe5 95% | MO 205β: Fe5 94% | MO 341β: Fe5 90% & Mo 8% | |
| $Fe_6$ | MO 203α: Fe6 99% | MO 207α: Fe6 96% | MO 208α: Fe6 97% | MO 345α: Fe6 89% & Mo 8% | MO 346α: Fe6 81% & Fe2 5% | MO 416β: Fe6 69.80% Fe7 13% 110C 10% |
| $Fe_7$ | MO 210α: Fe7 97% | MO 212α: Fe7 99% | MO 215α: Fe7 93% | MO 216α: Fe7 93% | MO 347α: Fe7 84% & Mo 14% | |

| | α | β |
|---|---|---|
| H | MO 403α: H 53% & Fe2 41% | MO 410β: Fe2 22.00% Fe6 23% H 60% |

**Table S14.** Localized orbital populations of the **$E_2$-hydTS** BS7-235 state. α-195$^{His}$-$N_ε$(H).

| | $d^1$ | $d^2$ | $d^3$ | $d^4$ | $d^5$ | $d^6$ |
|---|---|---|---|---|---|---|
| Mo | MO 346α: Fe5 45% & Mo 52% | MO 342β: Fe6 28% & Mo 69% | MO 343β: Fe7 33% & Mo 66% | | | |
| $Fe_1$ | MO 173α: Fe1 99% | MO 174α: Fe1 99% | MO 175α: Fe1 97% | MO 176α: Fe1 94% | MO 177α: Fe1 99% | MO 340β: Fe4 23% & Fe1 74% |
| $Fe_2$ | MO 177β: Fe2 98% | MO 178β: Fe2 99% | MO 182β: Fe2 96% | MO 183β: Fe2 96% | MO 411β: Fe2 75% Fe4 10% | MO 345α: Fe3 12% & Fe2 80% |

| | | | | | | |
|---|---|---|---|---|---|---|
| $Fe_3$ | MO 185β: Fe3 99% | MO 188β: Fe3 98% | MO 190β: Fe3 94% | MO 191β: Fe3 95% | MO 192β: Fe3 94% | |
| $Fe_4$ | MO 190α: Fe4 99% | MO 191α: Fe4 95% | MO 192α: Fe4 99% | MO 196α: Fe4 92% | MO 197α: Fe4 95% | |
| $Fe_5$ | MO 198β: Fe5 99% | MO 201β: Fe5 96% | MO 203β: Fe5 95% | MO 204β: Fe5 94% | MO 341β: Fe5 90% & Mo 8% | |
| $Fe_6$ | MO 204α: Fe6 99% | MO 207α: Fe6 93% | MO 208α: Fe6 94% | MO 209α: Fe6 96% | MO 347α: Fe6 84% & Mo 13% | |
| $Fe_7$ | MO 214α: Fe7 99% | MO 215α: Fe7 95% | MO 216α: Fe7 94% | MO 217α: Fe7 94% | MO 348α: Fe7 83% & Mo 14% | MO 344β: Fe7 63% & Fe6 26% |

| | α | β |
|---|---|---|
| H | MO 405α: H1 57% & Fe2 37% | MO 415β: Fe2 16% Fe4 12% H2 13% H1 52% |

**Table S15.** Localized orbital populations of the **$E_2$-nohyd** BS7-346 state. α-195$^{His}$-$N_\varepsilon$(H).

| | $d^1$ | $d^2$ | $d^3$ | $d^4$ | $d^5$ | $d^6$ |
|---|---|---|---|---|---|---|
| Mo | MO 345β: Fe5 35% & Mo 62% | MO 347α: Fe6 23% & Mo 73% | MO 347β: Fe7 37% & Mo 62% | | | |
| $Fe_1$ | MO 173α: Fe1 99% | MO 174α: Fe1 99% | MO 175α: Fe1 93% | MO 176α: Fe1 96% | MO 180α: Fe1 99% | MO 344β: Fe1 86% & S(Cys275) 1% |
| $Fe_2$ | MO 185α: Fe2 99% | MO 186α: Fe2 91% | MO 187α: Fe2 93% | MO 188α: Fe2 96% | MO 189α: Fe2 95% | MO 180β: Fe2 95% |
| $Fe_3$ | MO 182β: Fe3 99% | MO 186β: Fe3 95% | MO 187β: Fe3 96% | MO 188β: Fe3 95% | MO 189β: Fe3 96% | MO 345α: Fe4 22% & Fe3 69% |
| $Fe_4$ | MO 191β: Fe4 98% | MO 192β: Fe4 96% | MO 193β: Fe4 99% | MO 197β: Fe4 96% | MO 198β: Fe4 96% | |
| $Fe_5$ | MO 199α: Fe5 99% | MO 201α: Fe5 97% | MO 204α: Fe5 95% | MO 205α: Fe5 95% | MO 346α: Fe5 88% & Mo 9% | |
| $Fe_6$ | MO 207β: Fe6 95% | MO 208β: Fe6 91% | MO 209β: Fe6 95% | MO 210β: Fe6 92% | MO 346β: Fe6 82% & Mo 13% | MO 210α: Fe6 96% |
| $Fe_7$ | MO 215α: Fe7 99% | MO 216α: Fe7 96% | MO 217α: Fe7 95% | MO 218α: Fe7 95% | MO 348α: Fe7 85% & Mo 13% | MO 348β: Fe7 72% & Fe5 18% |

**Exploration of multiple BS solutions for CPCM models : bH(2,6)-CBS(S2B), bH(4,5)-OBS(5), noH-CBS(S2B,S5A), tH(5)-CBS(S2B).**

The lowest energy models of each structural class in Figure 3 (bottom row) were chosen (with the exeption of **bH(2,6)-CBS(S2B)**) and ~28 BS solutions were calculated (full geometry optimization with ZORA-TPSSh in a CPCM continuum). BS solutions 123,124,134,234 and 567 were skipped as they were found to always involve an unfavorable Mo(III) Hund configuration resulting in an unfavorable state energy of >20 kcal/mol (see **bH(4,5)-OBS(5)-134** in Table S16 for an example). Energies and Mulliken spin populations of all states are shown in Tables S16-S19.

**Table S16.** Calculated relative energies in kcal/mol (TPSSh level of theory using the minimal cluster model) and Mulliken spin populations on the metal ions for all broken-symmetry solutions of the $E_2$ **bH(2,6)-CBS(S2B)** models shown in Fig. 3 of article.

| Model | Energy | Mo | $Fe_1$ | $Fe_2$ | $Fe_3$ | $Fe_4$ | $Fe_5$ | $Fe_6$ | $Fe_7$ |
|---|---|---|---|---|---|---|---|---|---|
| **bH(2,6)-CBS(S2B)-125** | 23.5 | -0.30 | -2.03 | -2.77 | 3.45 | 3.34 | -3.03 | 0.87 | 2.93 |
| **bH(2,6)-CBS(S2B)-126** | 6.5 | -0.62 | -3.46 | -3.13 | 2.95 | 2.74 | 3.04 | -1.87 | 3.06 |
| **bH(2,6)-CBS(S2B)-127** | 6.5 | -0.14 | -3.48 | -2.98 | 3.44 | 3.48 | 3.15 | 1.99 | -2.76 |
| **bH(2,6)-CBS(S2B)-135** | 4.2 | -0.55 | -3.42 | 2.77 | -3.21 | 3.41 | -2.51 | 3.15 | 3.21 |
| **bH(2,6)-CBS(S2B)-137** | 6.7 | -0.32 | -3.43 | 3.29 | -3.11 | 3.49 | 3.21 | 2.38 | -2.59 |
| **bH(2,6)-CBS(S2B)-145** | 9.4 | -0.56 | -3.46 | 3.31 | 3.50 | -3.10 | -2.63 | 2.61 | 3.24 |
| **bH(2,6)-CBS(S2B)-146** | 2.9 | -0.89 | -3.46 | 2.96 | 2.94 | -3.44 | 2.86 | -0.52 | 2.99 |
| **bH(2,6)-CBS(S2B)-147** | 12.0 | -0.43 | -2.14 | 3.02 | 3.35 | -3.16 | 3.07 | 2.25 | -2.94 |
| **bH(2,6)-CBS(S2B)-156** | 9.7 | 0.27 | -3.56 | 3.12 | 2.42 | 3.05 | -3.14 | -1.25 | 2.66 |

| | | | | | | | | | |
|---|---|---|---|---|---|---|---|---|---|
| **bH(2,6)-CBS(S2B)-157** | 4.9 | 0.50 | -3.25 | 3.07 | 3.31 | 3.26 | -3.03 | 2.45 | -3.04 |
| **bH(2,6)-CBS(S2B)-167** | 13.4 | 0.53 | -3.55 | 3.19 | 3.04 | 2.22 | 2.73 | -1.62 | -3.12 |
| **bH(2,6)-CBS(S2B)-236** | 5.1 | -0.27 | 3.47 | -3.06 | -3.41 | 3.20 | 1.90 | -2.42 | 2.94 |
| **bH(2,6)-CBS(S2B)-237** | 8.8 | -0.25 | 3.50 | -3.09 | -3.36 | 3.49 | 3.05 | 2.01 | -2.91 |
| **bH(2,6)-CBS(S2B)-245** | 6.5 | -0.62 | 3.47 | -3.24 | 3.38 | -3.39 | -3.04 | 2.94 | 3.10 |
| **bH(2,6)-CBS(S2B)-246** | 3.2 | -0.34 | 3.45 | -3.25 | 3.14 | -3.43 | 2.89 | -2.06 | 2.20 |
| **bH(2,6)-CBS(S2B)-247** | 0.0 | -0.52 | 3.49 | -3.15 | 3.38 | -3.26 | 2.96 | 2.83 | -3.15 |
| **bH(2,6)-CBS(S2B)-256** | 16.9 | 0.34 | 2.51 | -3.31 | 2.69 | 3.11 | -3.19 | -1.99 | 2.76 |
| **bH(2,6)-CBS(S2B)-257** | 17.9 | 0.54 | 2.50 | -3.01 | 3.27 | 3.26 | -2.90 | 1.97 | -2.94 |
| **bH(2,6)-CBS(S2B)-267** | 15.9 | 0.56 | 3.38 | -3.16 | 3.28 | 0.77 | 2.91 | -2.23 | -3.15 |
| **bH(2,6)-CBS(S2B)-345** | 3.8 | -0.63 | 3.47 | 3.16 | -3.23 | -2.78 | -2.77 | 2.33 | 3.20 |
| **bH(2,6)-CBS(S2B)-346** | 1.6 | -0.30 | 3.49 | 3.21 | -3.30 | -3.27 | 2.95 | -3.03 | 2.97 |
| **bH(2,6)-CBS(S2B)-347** | 1.0 | -0.33 | 3.49 | 3.30 | -2.97 | -3.26 | 3.19 | 2.01 | -2.77 |
| **bH(2,6)-CBS(S2B)-356** | 0.7 | -0.45 | 3.48 | -3.23 | -3.28 | 3.40 | -2.58 | 2.10 | 3.14 |
| **bH(2,6)-CBS(S2B)-357** | 5.2 | 0.48 | 3.45 | 3.10 | -3.37 | 3.45 | -3.11 | 2.14 | -3.07 |
| **bH(2,6)-CBS(S2B)-367** | 10.2 | 0.51 | 3.50 | 2.28 | -3.44 | 3.34 | 2.98 | -3.13 | -3.13 |
| **bH(2,6)-CBS(S2B)-457** | 4.6 | 0.53 | 3.45 | 3.23 | 3.41 | -3.34 | -3.09 | 1.94 | -3.03 |
| **bH(2,6)-CBS(S2B)-467** | 5.6 | 0.64 | 3.43 | 2.38 | 3.31 | -3.42 | 2.93 | -3.07 | -3.08 |

**Table S17.** Calculated relative energies in kcal/mol (TPSSh level of theory using the minimal cluster model) and Mulliken spin populations on the metal ions for all broken-symmetry solutions of the $E_2$ **bH(4,5)-OBS(5)** models shown in Fig. 3 of article.

| Model | Energy | Mo | $Fe_1$ | $Fe_2$ | $Fe_3$ | $Fe_4$ | $Fe_5$ | $Fe_6$ | $Fe_7$ |
|---|---|---|---|---|---|---|---|---|---|
| **bH(4,5)-OBS(5)-125** | 8.1 | -0.60 | -3.49 | -3.29 | 3.46 | 3.23 | -2.94 | 3.10 | 3.20 |
| **bH(4,5)-OBS(5)-126** | 11.7 | -0.32 | -3.46 | -3.22 | 3.47 | 3.29 | 2.73 | -2.66 | 3.13 |
| **bH(4,5)-OBS(5)-127** | 9.3 | -0.47 | -3.44 | -3.13 | 3.34 | 3.40 | 3.14 | 3.14 | -3.00 |
| **bH(4,5)-OBS(5)-134** | 23.2 | -1.32 | -2.55 | 3.42 | -3.22 | -2.90 | 2.72 | 3.16 | 3.21 |
| **bH(4,5)-OBS(5)-136** | 7.9 | -0.44 | -3.42 | 3.31 | -3.17 | 3.42 | 3.12 | -3.02 | 3.11 |
| **bH(4,5)-OBS(5)-137** | 11.6 | -0.37 | -3.44 | 3.42 | -3.21 | 3.25 | 2.80 | 3.12 | -2.62 |
| **bH(4,5)-OBS(5)-145** | 13.3 | -0.51 | -3.49 | 3.36 | 3.36 | -3.28 | -3.08 | 3.09 | 3.10 |
| **bH(4,5)-OBS(5)-146** | 8.7 | -0.45 | -3.48 | 3.33 | 3.49 | -3.25 | 3.08 | -3.02 | 3.17 |
| **bH(4,5)-OBS(5)-147** | 20.6 | -0.21 | -3.39 | 3.20 | 3.24 | -1.65 | 2.86 | 2.28 | -3.13 |
| **bH(4,5)-OBS(5)-156** | 6.8 | 0.48 | -3.58 | 3.16 | 3.20 | 3.10 | -2.92 | -2.90 | 2.79 |
| **bH(4,5)-OBS(5)-157** | 6.6 | 0.42 | -3.58 | 3.16 | 3.12 | 3.08 | -2.83 | 2.78 | -2.85 |
| **bH(4,5)-OBS(5)-167** | 5.6 | 0.61 | -3.60 | 3.29 | 3.27 | 2.90 | 2.98 | -2.90 | -2.95 |
| **bH(4,5)-OBS(5)-235** | 12.3 | 0.71 | -3.27 | 3.38 | 3.22 | 3.02 | 2.50 | -3.10 | -3.14 |
| **bH(4,5)-OBS(5)-236** | 7.8 | -0.33 | 3.50 | -3.32 | -3.34 | 3.38 | 2.83 | -2.93 | 3.06 |
| **bH(4,5)-OBS(5)-237** | 9.6 | -0.40 | 3.50 | -3.32 | -3.37 | 3.32 | 3.11 | 3.07 | -2.99 |
| **bH(4,5)-OBS(5)-245** | 8.4 | -0.61 | 3.45 | -3.34 | 3.29 | -3.15 | -3.01 | 3.03 | 2.73 |
| **bH(4,5)-OBS(5)-246** | 6.3 | -0.33 | 3.47 | -3.37 | 3.39 | -3.27 | 2.89 | -3.01 | 2.95 |
| **bH(4,5)-OBS(5)-247** | 0.0 | -0.39 | 3.48 | -3.26 | 3.36 | -3.23 | 2.94 | 2.94 | -3.11 |
| **bH(4,5)-OBS(5)-256** | 20.7 | 0.47 | 3.46 | -3.40 | 2.98 | 3.01 | -3.13 | -2.99 | 2.57 |
| **bH(4,5)-OBS(5)-257** | 15.7 | 0.44 | 2.48 | -3.36 | 3.22 | 3.02 | -2.98 | 3.07 | -2.96 |
| **bH(4,5)-OBS(5)-267** | 14.5 | 0.79 | 3.58 | -3.44 | 3.38 | 3.13 | 2.05 | -3.14 | -3.15 |
| **bH(4,5)-OBS(5)-345** | 9.0 | -0.62 | 3.47 | 3.27 | -3.37 | -3.12 | -2.97 | 2.72 | 3.02 |
| **bH(4,5)-OBS(5)-346** | 0.4 | -0.39 | 3.49 | 3.34 | -3.28 | -3.23 | 2.94 | -3.12 | 2.95 |
| **bH(4,5)-OBS(5)-347** | 7.3 | -0.38 | 3.48 | 3.37 | -3.39 | -3.26 | 2.93 | 2.96 | -3.01 |
| **bH(4,5)-OBS(5)-356** | 15.8 | 0.45 | 2.56 | 3.18 | -3.39 | 3.03 | -2.99 | -2.96 | 3.07 |
| **bH(4,5)-OBS(5)-357** | 21.2 | 0.36 | 2.54 | 3.25 | -3.39 | 3.13 | -2.99 | 2.99 | -2.98 |
| **bH(4,5)-OBS(5)-367** | 13.8 | 0.79 | 3.45 | 3.36 | -3.43 | 3.25 | 2.01 | -3.07 | -3.17 |
| **bH(4,5)-OBS(5)-456** | 21.1 | 0.51 | 3.43 | 3.12 | 2.70 | -3.36 | -3.20 | -3.17 | 2.80 |
| **bH(4,5)-OBS(5)-457** | 21.9 | 0.39 | 2.46 | 3.21 | 3.21 | -3.29 | -3.10 | 2.93 | -3.07 |
| **bH(4,5)-OBS(5)-467** | 16.2 | 0.58 | 3.62 | 3.12 | 3.25 | -3.34 | 2.48 | -3.24 | -3.24 |

**Table S18.** Calculated relative energies in kcal/mol (TPSSh level of theory using the minimal cluster model) and Mulliken spin populations on the metal ions for all broken-symmetry solutions of the $E_2$ **noH-CBS(S2B,S5A)** models shown in Fig. 3 of article.

| Model | Energy | Mo | $Fe_1$ | $Fe_2$ | $Fe_3$ | $Fe_4$ | $Fe_5$ | $Fe_6$ | $Fe_7$ |
|---|---|---|---|---|---|---|---|---|---|
| **noH-CBS(S2B,S5A)-125** | 11.0 | -0.70 | -3.44 | -2.94 | 3.47 | 3.37 | -2.87 | 2.80 | 3.14 |

| | | | | | | | | | |
|---|---|---|---|---|---|---|---|---|---|
| **noH-CBS(S2B,S5A)-126** | 8.4 | -0.43 | -3.48 | -3.14 | 3.25 | 3.41 | 3.15 | -2.77 | 2.86 |
| **noH-CBS(S2B,S5A)-127** | 10.5 | -0.34 | -3.50 | -3.26 | 3.29 | 3.33 | 3.13 | 2.92 | -2.60 |
| **noH-CBS(S2B,S5A)-136** | 9.2 | -0.36 | -3.49 | 3.26 | -3.30 | 3.30 | 3.16 | -2.51 | 2.92 |
| **noH-CBS(S2B,S5A)-137** | 7.2 | -0.60 | -3.47 | 3.25 | -3.16 | 3.38 | 3.13 | 2.99 | -2.71 |
| **noH-CBS(S2B,S5A)-145** | 10.7 | -0.56 | -3.47 | 3.24 | 3.42 | -3.19 | -2.65 | 3.01 | 3.14 |
| **noH-CBS(S2B,S5A)-146** | 7.5 | -0.36 | -3.47 | 3.31 | 3.32 | -3.34 | 3.05 | -2.50 | 3.01 |
| **noH-CBS(S2B,S5A)-147** | 25.4 | 0.39 | -1.84 | 3.31 | 3.36 | -2.90 | 3.12 | -1.92 | -1.39 |
| **noH-CBS(S2B,S5A)-156** | 7.5 | 0.30 | -3.58 | 3.20 | 3.30 | 3.30 | -3.00 | -2.85 | 2.73 |
| **noH-CBS(S2B,S5A)-157** | 5.2 | 0.41 | -3.27 | 3.28 | 3.22 | 3.26 | -3.11 | 2.33 | -3.05 |
| **noH-CBS(S2B,S5A)-167** | 11.7 | 0.54 | -3.27 | 3.11 | 3.16 | 2.61 | 2.83 | -3.03 | -3.04 |
| **noH-CBS(S2B,S5A)-235** | 5.2 | -0.59 | 3.47 | -3.22 | -3.29 | 3.41 | -2.69 | 2.59 | 2.93 |
| **noH-CBS(S2B,S5A)-236** | 9.1 | -0.48 | 3.47 | -3.07 | -3.38 | 3.35 | 3.10 | -3.01 | 2.68 |
| **noH-CBS(S2B,S5A)-237** | 10.7 | -0.36 | 3.45 | -3.41 | -3.39 | 3.25 | 2.90 | 2.99 | -2.66 |
| **noH-CBS(S2B,S5A)-245** | 9.0 | -0.56 | 3.49 | -3.14 | 3.32 | -3.42 | -2.94 | 3.09 | 2.85 |
| **noH-CBS(S2B,S5A)-246** | 5.3 | -0.26 | 3.47 | -3.37 | 3.25 | -3.46 | 3.04 | -2.64 | 2.77 |
| **noH-CBS(S2B,S5A)-247** | 0.7 | -0.40 | 3.44 | -3.29 | 3.05 | -3.36 | 3.06 | 2.95 | -2.61 |
| **noH-CBS(S2B,S5A)-256** | 13.5 | 0.51 | 3.44 | -3.33 | 2.72 | 3.21 | -3.21 | -3.03 | 2.82 |
| **noH-CBS(S2B,S5A)-257** | 6.5 | 0.44 | 3.39 | -3.38 | 3.16 | 3.23 | -3.11 | 2.38 | -3.05 |
| **noH-CBS(S2B,S5A)-267** | 15.1 | 0.56 | 3.42 | -3.39 | 3.05 | 3.15 | 2.45 | -3.11 | -3.19 |
| **noH-CBS(S2B,S5A)-345** | 7.9 | -0.57 | 3.50 | 3.33 | -3.13 | -3.38 | -2.91 | 2.75 | 3.09 |
| **noH-CBS(S2B,S5A)-346** | 0.0 | -0.32 | 3.47 | 3.06 | -3.34 | -3.35 | 3.01 | -2.64 | 3.00 |
| **noH-CBS(S2B,S5A)-347** | 7.9 | -0.36 | 3.46 | 3.03 | -3.42 | -3.43 | 3.11 | 3.03 | -2.58 |
| **noH-CBS(S2B,S5A)-356** | 6.4 | 0.45 | 3.37 | 3.14 | -3.40 | 3.22 | -3.10 | -3.05 | 2.43 |
| **noH-CBS(S2B,S5A)-357** | 8.9 | 0.37 | 3.40 | 3.24 | -3.37 | 3.29 | -3.17 | 2.35 | -3.13 |
| **noH-CBS(S2B,S5A)-367** | 14.3 | 0.55 | 3.41 | 3.06 | -3.40 | 3.09 | 2.45 | -3.20 | -3.07 |
| **noH-CBS(S2B,S5A)-457** | 6.6 | 0.36 | 3.46 | 3.28 | 3.24 | -3.46 | -3.15 | 2.47 | -3.15 |
| **noH-CBS(S2B,S5A)-467** | 3.1 | 0.49 | 3.44 | 3.11 | 3.13 | -3.48 | 2.58 | -3.09 | -3.10 |

**Table S19.** Calculated relative energies in kcal/mol (TPSSh level of theory using the minimal cluster model) and Mulliken spin populations on the metal ions for all broken-symmetry solutions of the $E_2$ **tH(5)-CBS(S2B)** models shown in Fig. 3 of article.

| Model | Energy | Mo | $Fe_1$ | $Fe_2$ | $Fe_3$ | $Fe_4$ | $Fe_5$ | $Fe_6$ | $Fe_7$ |
|---|---|---|---|---|---|---|---|---|---|
| **tH(5)-CBS(S2B)-125** | 13.0 | -0.82 | -3.49 | -3.16 | 3.45 | 3.35 | -2.51 | 2.60 | 3.23 |
| **tH(5)-CBS(S2B)-126** | 3.5 | -0.26 | -3.48 | -3.13 | 3.43 | 3.38 | 2.44 | -2.74 | 3.07 |
| **tH(5)-CBS(S2B)-127** | 5.8 | -0.21 | -3.48 | -2.91 | 3.35 | 3.45 | 2.49 | 3.04 | -2.86 |
| **tH(5)-CBS(S2B)-135** | 7.7 | -0.69 | -3.45 | 3.23 | -3.30 | 3.31 | -2.05 | 2.77 | 3.02 |
| **tH(5)-CBS(S2B)-136** | 1.9 | -0.26 | -3.46 | 3.29 | -3.32 | 3.44 | 2.47 | -2.36 | 3.07 |
| **tH(5)-CBS(S2B)-137** | 6.6 | -0.36 | -3.44 | 3.39 | -3.15 | 3.43 | 2.45 | 3.08 | -2.56 |
| **tH(5)-CBS(S2B)-145** | 8.8 | -0.60 | -3.46 | 2.93 | 3.23 | -3.19 | -2.09 | 3.00 | 3.12 |
| **tH(5)-CBS(S2B)-146** | 5.9 | -0.31 | -3.48 | 3.30 | 3.45 | -3.17 | 2.38 | -2.57 | 3.22 |
| **tH(5)-CBS(S2B)-147** | 5.7 | -0.27 | -3.49 | 3.41 | 3.41 | -3.06 | 2.43 | 3.11 | -2.72 |
| **tH(5)-CBS(S2B)-156** | 14.0 | 0.36 | -3.56 | 3.01 | 2.85 | 3.11 | -2.34 | -2.96 | 2.87 |
| **tH(5)-CBS(S2B)-157** | 7.3 | 0.19 | -3.57 | 3.20 | 3.20 | 3.17 | -2.32 | 2.52 | -2.98 |
| **tH(5)-CBS(S2B)-167** | 5.7 | 0.59 | -3.57 | 3.11 | 3.25 | 3.32 | 2.59 | -2.85 | -2.94 |
| **tH(5)-CBS(S2B)-235** | 7.7 | -0.61 | 3.45 | -3.20 | -3.29 | 2.92 | -2.59 | 2.99 | 3.01 |
| **tH(5)-CBS(S2B)-236** | 2.6 | -0.28 | 3.46 | -3.10 | -3.34 | 3.33 | 2.39 | -2.97 | 3.09 |
| **tH(5)-CBS(S2B)-237** | 5.4 | -0.25 | 3.46 | -3.27 | -3.19 | 3.33 | 2.44 | 3.06 | -2.88 |
| **tH(5)-CBS(S2B)-245** | 8.7 | -0.73 | 3.46 | -3.29 | 3.39 | -3.34 | -2.59 | 2.55 | 3.12 |
| **tH(5)-CBS(S2B)-246** | 7.0 | -0.20 | 3.45 | -3.34 | 3.33 | -3.40 | 2.37 | -2.60 | 3.09 |
| **tH(5)-CBS(S2B)-247** | 2.6 | -0.26 | 3.45 | -3.24 | 3.37 | -3.27 | 2.44 | 2.99 | -2.73 |
| **tH(5)-CBS(S2B)-256** | 20.7 | 0.44 | 3.39 | -3.22 | 1.25 | 3.25 | -2.50 | -3.05 | 2.94 |
| **tH(5)-CBS(S2B)-257** | 11.4 | 0.45 | 3.38 | -3.30 | 3.11 | 2.57 | -2.33 | 2.42 | -3.11 |
| **tH(5)-CBS(S2B)-267** | 14.6 | 0.64 | 3.40 | -3.31 | 3.32 | 3.11 | 2.27 | -3.13 | -3.17 |
| **tH(5)-CBS(S2B)-345** | 5.4 | -0.64 | 3.49 | 3.27 | -3.42 | -3.35 | -2.46 | 2.77 | 3.05 |
| **tH(5)-CBS(S2B)-346** | 0.0 | -0.34 | 3.50 | 3.25 | -3.29 | -3.27 | 2.31 | -2.60 | 3.08 |
| **tH(5)-CBS(S2B)-347** | 10.2 | -0.28 | 3.50 | 3.27 | -3.30 | -3.24 | 2.50 | 3.04 | -2.71 |
| **tH(5)-CBS(S2B)-356** | 8.9 | 0.26 | 3.45 | 2.94 | -3.44 | 2.63 | -2.40 | -3.05 | 2.61 |
| **tH(5)-CBS(S2B)-357** | 13.0 | 0.24 | 3.46 | 3.10 | -3.45 | 2.82 | -2.46 | 2.53 | -3.10 |
| **tH(5)-CBS(S2B)-367** | 11.5 | 0.55 | 3.45 | 3.27 | -3.46 | 3.21 | 2.39 | -3.12 | -3.14 |
| **tH(5)-CBS(S2B)-456** | 12.1 | 0.33 | 3.42 | 3.07 | 2.46 | -3.41 | -2.39 | -3.13 | 2.60 |
| **tH(5)-CBS(S2B)-457** | 15.6 | 0.27 | 2.50 | 3.06 | 3.18 | -3.34 | -2.38 | 2.92 | -3.11 |
| **tH(5)-CBS(S2B)-467** | 11.1 | 0.65 | 3.41 | 3.07 | 3.18 | -3.47 | 2.53 | -3.05 | -3.02 |
